# Synthesis, biological activity and multiscale molecular modeling studies for coumaryl-carboxamide derivatives as selective carbonic anhydrase IX inhibitors

**DOI:** 10.1080/14756366.2017.1354857

**Published:** 2017-08-04

**Authors:** Belma Zengin Kurt, Fatih Sonmez, Serdar Durdagi, Busecan Aksoydan, Ramin Ekhteiari Salmas, Andrea Angeli, Mustafa Kucukislamoglu, Claudiu T. Supuran

**Affiliations:** a Faculty of Pharmacy, Department of Pharmaceutical Chemistry, Bezmialem Vakıf University, Istanbul, Turkey;; b Faculty of Arts and Science, Department of Chemistry, Sakarya University, Sakarya, Turkey;; c Computational Biology and Molecular Simulations Laboratory, Department of Biophysics, School of Medicine, Bahcesehir University, Istanbul, Turkey;; d Dipartimento Neurofarba, Sezione di ScienzeFarmaceutiche e Nutraceutiche, Università degli Studi di Firenze, Florence, Italy

**Keywords:** Coumarin, carboxamid, thiourea, carbonic anhydrase, molecular docking, induced fit docking, quantum polarised ligand docking

## Abstract

New coumaryl-carboxamide derivatives with the thiourea moiety as a linker between the alkyl chains and/or the heterocycle nucleus were synthesized and their inhibitory activity against the human carbonic anhydrase (hCA) isoforms hCA I, II, VII and IX were evaluated. While the hCA I, II and VII isoforms were not inhibited by the investigated compounds, the tumour-associated isoform hCA IX was inhibited in the high nanomolar range. 2-Oxo-*N*-((2-(pyrrolidin-1-yl)ethyl)carbamothioyl)-*2H*-chromene-3-carboxamide (**e11**) exhibited a selective inhibitory action against hCA IX with the *K*_i_ of 107.9 nM. In order to better understand the inhibitory profiles of studied molecules, multiscale molecular modeling approaches were used. Different molecular docking algorithms were used to investigate binding poses and predicted binding energies of studied compounds at the active sites of the CA I, II, VII and IX isoforms.

## 1. Introduction

The carbonic anhydrases (CAs; EC 4.2.1.1) are a superfamily of metalloenzymes that present in all organisms and consist of metallic core of Zn^2+^ ion at their active center^1–4^. CA, explored in the beef erythrocytes for the first time, reversibly catalyses the reactions of hydration of CO_2_ and dehydration^5^ of HCO_3_^-^. Many CA isozymes involved in these processes are important therapeutic targets with the potential to be inhibited/activated for the treatment of a range of disorders such as oedema, glaucoma, obesity, cancer, epilepsy, amyloid beta, leukaemia and osteoporosis^6–10^. However, the physiologically relevant reaction that CAs catalyse, using as substrates CO_2_, COS, CS_2_, cyanamide, carboxylic, phosphoric and thiocarboxylicesters^11–14^. Sixteen different α-CA isoforms were isolated from mammals, where they play crucial physiological roles. Some of them are cytosolic (CA I, CA II, CA III, CA VII, CA XIII), others are membrane-bound (CA IV, CA IX, CA XII, CA XIV and CA XV), CA VA and CA VB are mitochondrial, and CA VI is secreted in saliva and milk^11^. Recent studies suggested that the necrosis formed around a tumour depends on both the excessively expressing of CA IX enzymes increased at such domain and the controlling of pH^15^^,^^16^. Especially, hCA IX is expressed in a restricted number of normal tissues, whereas it is over expressed in many solid tumours and considered involved in important processes connected with cancer progression. The over expression of hCA IX induces the pH imbalance of tumour tissue contributing significantly to the extracellular acidification of solid tumour; thereby hCA IX inhibitors could specifically bind hypoxic tumour cells expressing this isoform^17–24^. Therefore, it has been considered that the CA inhibitors are crucial molecules for the synthesis of new-generation anticancer drugs^25^.

Coumarin is one of the most known class of CA inhibitors (CAIs) and shows their effect as 2-hydroxycinnamic acid hydrolysis, unlike other inhibitors^26^^,^^27^. Coumarin derivatives show high selectivity to inhibit isoforms, especially in pharmacological applications, such as the tumour-associated ones (hCA IX and XII, which are targets for antitumour/antimetastatic drugs) or the mitochondrial ones (CA VA and VB, which are targets for antiobesity agents)^28^^,^^29^. Thiocoumarin, thioxocoumarin and sulphocoumarin derivatives showed high affinity for CA IX-XII even at low concentrations^30–32^.

Urea and thiourea compounds work as building block in the synthesis of heterocyclic compounds. These compounds, thanks to their pharmacological properties, make a significant contribution in the field of medicinal chemistry. Urea and thiourea derivatives exhibit many biological activities such as analgesic, anti-inflammatory, antimicrobial and anticancer. Thiourea derivatives are valuable building blocks for the synthesis of amides, guanidines and varieties of heterocycles^33^^,^^34^. It has been reported that compounds containing urea or thiourea as well as sulphonamide groups highly inhibit the enzyme carbonic anhydrase^35–37^.

Continuing our interest in coumarin CAIs, in this work, we report the synthesis of novel thiourea-substituted coumaryl-carboxamid derivatives and their effects on the inhibitory activity of human carbonic anhydrase hCA I, hCA II, hCA VII and hCA IX.

## 2. Experimental

### 2.1. Material and method

Melting points were taken on a Barnstead Electrothermal 9200. IR spectra were measured on a Shimadzu Prestige-21 (200 VCE) spectrometer. ^1^H and ^13^C NMR spectra were measured on a Varian Infinity Plus spectrometer at 300 and at 75 Hz, respectively. ^1^H and ^13^C chemical shifts are referenced to the internal deuterated solvent. Mass spectra were obtained using MICROMASS Quattro LC-MS-MS spectrometer. The elemental analyses were carried out with a Leco CHNS-932 instrument. Spectrophotometric analyses were performed by a BioTek Power Wave XS (Winooski, VT). The chemicals and solvents were purchased from Fluka Chemie (Taufkirchen, Germany), Merck (Taufkirchen, Germany), Alfa Aesar (Taufkirchen, Germany) and Sigma-Aldrich (Taufkirchen, Germany).

### 2.2. General procedures of synthesis and spectral data

#### 2.2.1. 2-oxo-2H-chromene-3-carboxylic acid (c)

A mixture of benzaldehyde (**a**) (3 mmol), meldrum’s acid (**b**) (4.5 mmol) was stirred at reflux for 10 h. The mixture was cooled, filtered and recrystallised from methanol to get product (**c**). Spectral data of this compound were matched with the literature, white solid, 91% yield; mp. 145–147 °C^38^.

#### 2.2.2. 2-oxo-2H-chromene-3-carbonyl chloride (d)

A 2-oxo-2H-chromene-3-carboxylic acid (**c**) (0.01 mol) and SOCl_2_ (0.05 mol) were taken in round bottom flask and it was stirred for 4 h at 80 °C temperature. After the excess SOCl_2_ was evaporated, the crude product was purified by ether. Spectral data of this compound was matched with the literature^50^.

White solid, 99% yield; IR: 3057, 1737, 1676, 1605, 1557, 1417, 1225, 1179, 1040, 761 cm^−1^; ^1^H NMR (DMSO-d_6_, 300 MHz) *δ*/ppm: 7.36–7.43 (2H, *m*), 7.69 (1H, td, *J* = 1.7, 7.3 Hz), 7.87 (1H, dd, *J* = 1.4, 7.6 Hz), 8.71 (1H, *s*); ^13^C NMR (DMSO-d_6_, 75 MHz) *δ*/ppm:, 116.7, 118.6, 118.9, 125.4, 130.8, 134.9, 149.1, 155.1, 157.3, 164.5.

#### 2.2.3. N-(R-carbamothioyl)-2-oxo-2H-chromene-3-carboxamide (e1–20)

A mixture of 2-oxo-2H-chromene-3-carbonyl chloride (1 mmol) and KSCN (1,2 mmol) in CH_3_CN (30 ml) was heated under reflux for 30 min. Then, 1.2 mmol amine derivatives were added in the mixture and the solution was refluxed for 4 h. The solution was evaporated and the residue was extracted with water/CH_2_Cl_2_. The organic phase was washed by water for three times and dried over Na_2_SO_4_. After the organic solvent was evaporated, the crude product was recrystallised from methanol to get pure crystalline **e1**–**20** in 25–70% yields.

#### 2.2.4. N-(methylcarbamothioyl)-2-oxo-2H-chromene-3-carboxamide (e1)

Yellow powder, 52% yield; mp. 146–148 °C; IR: 3363, 3052, 2978, 1698, 1655, 1606, 1562, 1510, 1449, 1243, 1159, 982, 754 cm^−1^; ^1^H NMR (CDCl_3_, 300 MHz) *δ*/ppm: 3.03 (CH_3_NH-, 3H, d, *J* = 4.9 Hz), 7.36–7.43 (2H, *m*), 7.64–7.76 (2H, *m*), 8.68 (1H, *s*, NH), 8.93 (1H, s); ^13^C NMR (DMSO-d_6_, 75 MHz) *δ*/ppm: 27.0, 116.7, 119.1, 119.6, 125.7, 130.9, 134.7, 147.9, 148.5, 154.5, 160.9, 162.2. LC-MS (*m/z)*: 300.1 [M^+^]. Anal. Calcd. for C_12_H_10_N_2_O_3_S; C, 54.95; H, 3.84; N, 10.68; found: C, 54.90; H, 3.86; N, 10.67.

#### 2.2.5. 2-oxo-N-(propylcarbamothioyl)-2H-chromene-3-carboxamide (e2)

Yellow powder, 58% yield; mp. 123 °C; IR: 3339, 3053, 2964, 1703, 1655, 1607, 1518, 1450, 1362, 1158, 755 cm^−1^; ^1^H NMR (CDCl_3_, 300 MHz) *δ*/ppm: 1.00 (3H, *t*, *J* = 7.6 Hz), 1.60–1.72 (2H, *m*), 3.40–4.47 (2H, *m*), 7.35–7.42 (2H, *m*), 7.64–7.71 (2H, *m*), 8.83 (1H, *s*, NH), 8.92 (1H, *s*); ^13^C NMR (CDCl_3_, 75 MHz) *δ*/ppm: 11.7, 22.9, 41.8, 116.8, 118.7, 118.9, 125.5, 130.0, 134.1, 148.4, 154.6, 161.6, 161.7. LC-MS (*m*/*z*): 321.2 [M^+^]. Anal. Calcd. for C_14_H_14_N_2_O_3_S; C, 57.92; H, 4.86; N, 9.65; found: C, 57.90; H, 4.85; N, 9.67.

#### 2.2.6. N-(diethylcarbamothioyl)-2-oxo-2H-chromene-3-carboxamide (e3)

Yellow powder, 40% yield; mp. 143 °C; IR: 3300, 3060, 2978, 1709, 1609, 1567, 1437, 1219, 1198, 1016, 763, 641 cm^−1^; ^1^H NMR (CDCl_3_, 300 MHz) *δ*/ppm: 1.35 (5H, *s*, br), 1.64 (1H, *s*), 3.63 (2H, *s*, br), 4.01 (2H, *s*, br), 7.27–7.46 (2H, *m*), 7.70–7.75 (2H, *m*), 8.96 (1H, *s*), 10.97 (N=C–SH, 1H, *s*, SH); ^13^C NMR (CDCl_3_, 75 MHz) *δ*/ppm: 11.5, 47.7, 117.0, 118.0, 118.6, 123.4, 125.9, 130.4, 135.2, 150.5, 154.8, 158.5, 161.7, 177.7. LC-MS (*m*/*z*): 321.1 [M^+^]. Anal. Calcd. for C_15_H_16_N_2_O_3_S; C, 59.19; H, 5.30; N, 9.20; found: C, 59.17; H, 5.31; N, 9.22.

#### 2.2.7. N-(diisopropylcarbamothioyl)-2-oxo-2H-chromene-3-carboxamide (e4)

Yellow powder, 70% yield; mp. 150–151 °C; IR: 3231, 3047, 2978, 1713, 1672, 1608, 1499, 1333, 1200, 1107, 757, 663 cm^−1^; ^1^H NMR (CDCl_3_, 300 MHz) *δ*/ppm: 1.23–1.58 (14H, *m*), 7.21–7.45 (2H, *m*), 7.71–7.75 (2H, *m*), 8.98 (1H, *s*), 10.68 (N=C-SH, 1H, *s*, SH); ^13^C NMR (CDCl_3_, 75 MHz) *δ*/ppm:, 21.0, 46.4, 51.8, 117.0, 118.1, 118.7, 125.0, 125.9, 130.4, 135.1, 150.5, 154.8, 161.7, 164.1. LC-MS (*m*/*z*): 333.3 [M^+^]. Anal. Calcd. for C_17_H_20_N_2_O_3_S; C, 61.42; H, 6.06; N, 8.43; found: C, 61.40; H, 6.07; N, 8.41.

#### 2.2.8. N-(cyclohexylcarbamothioyl)-2-oxo-2H-chromene-3-carboxamide (e5)

Yellow powder, 53% yield; mp. 164–166 °C; IR: 3321, 3051, 2925, 1704, 1665, 1609, 1523, 1452, 1366, 1163, 761, 610 cm^−1^; ^1^H NMR (CDCl_3_, 300 MHz) *δ*/ppm: 1.24–1.49 (4H, *s*), 1.61–1.65 (2H, *m*), 1.73–1.78 (2H, *m*), 1.97–2.00 (2H, *m*), 3.94–4.00 (1H, *m*), 7.35–7.42 (2H, *m*), 7.63–7.71 (2H, *m*), 8.76 (1H, d, *J* = 6.7 Hz, NH), 8.9 (1H, *s*); ^13^C NMR (CDCl_3_, 75 MHz) *δ*/ppm:, 24.9, 25.8, 32.9, 48.7, 116.8, 118.9, 125.4, 129.9, 134.1, 148.3, 150.9, 154.6, 160.6, 161.7. LC-MS (*m*/*z*): 330.2 [M^+^]. Anal. Calcd. for C_17_H_18_N_2_O_3_S; C, 61.80; H, 5.49; N, 8.48; found: C, 61.81; H, 5.47; N, 8.49.

#### 2.2.9. 2-oxo-N-(pyrrolidine-1-carbonothioyl)-2H-chromene-3-carboxamide (e6)

Yellow powder, 32% yield; mp. 162–163 °C; IR: 3321, 3051, 2925, 1704, 1665, 1609, 1523, 1452, 1366, 1163, 761, 610 cm^−1^; ^1^H NMR (CDCl_3_, 300 MHz) *δ*/ppm: 1.91–2.00 (4H, m), 3.46 (2H, t, J = 6.4 Hz), 3.64 (2H, t, J = 7.0 Hz), 7.27–7.38 (2H, m), 7.53–7.62 (2H, m), 7.97 (1H, s); ^13^C NMR (CDCl_3_, 75 MHz) *δ*/ppm:, 24.5, 26.2, 46.4, 47.7, 116.9, 118.5, 123.4, 125.1, 126.4, 128.8, 133.0, 143.3, 154.3, 158.0, 163.4. LC-MS (m/z): 303.3 [M^+^]. Anal. Calcd. for C_15_H_14_N_2_O_3_S; C, 59.59; H, 4.67; N, 9.27; found: C, 59.55; H, 4.68; N, 9.29.

#### 2.2.10. 2-oxo-N-(piperidine-1-carbonothioyl)-2H-chromene-3-carboxamide (e7)

Yellow powder, 45% yield; mp. 175–176 °C; IR: 3040, 2918, 1710, 1607, 1559, 1438, 1251, 1121, 1041, 757, 610 cm^−1^; ^1^H NMR (CDCl_3_, 300 MHz) *δ*/ppm: 1.64 (6H, *s*, br), 3.34 (2H, *s*, br), 3.71 (2H, *s*, br), 7.29–7.36 (2H, *m*), 7.52–7.61 (2H, *m*), 7.87 (1H, *s*); ^13^C NMR (CDCl_3_, 75 MHz) *δ*/ppm:, 24.5, 25.6, 26.4, 43.2, 48.5, 116.9, 118.6, 125.0, 126.0, 128.6, 132.8, 142.4, 154.1, 158.3, 163.5. LC-MS (*m*/*z*): 317.3 [M^+^]. Anal. Calcd. for C_16_H_16_N_2_O_3_S; C, 60.74; H, 5.10; N, 8.85; found: C, 60.76; H, 5.12; N, 8.81.

#### 2.2.11. N-(4-methylpiperazine-1-carbonothioyl)-2-oxo-2H-chromene-3-carboxamide (e8)

Yellow powder, 35% yield; mp.162–164 °C; IR: 3205, 3040, 2942, 1692, 1661, 1607, 1506, 1199, 1122, 792, 551 cm^−1^; ^1^H NMR (CDCl_3_, 300 MHz) *δ*/ppm: 2.36 (3H, *s*), 2.59 (4H, d, *J* = 8.2 Hz), 3.69 (2H, *s*, br), 4.27 (2H, *s*, br), 7.40–7.46 (2H, *m*), 7.71–7.77 (2H, *m*), 8.93 (1H, *s*), 11.05 (N=C-SH, 1H, *s*, SH); ^13^C NMR (CDCl_3_, 75 MHz) *δ*/ppm:, 36.3, 45.9, 51.8, 117.1, 117.5, 118.5, 125.9, 130.3, 135.4, 150.6, 155.0, 157.5, 161.3, 178.1. LC-MS (*m*/*z*): 354.2 [M^+^]. Anal. Calcd. for C_16_H_17_N_3_O_3_S; C, 57.99; H, 5.17; N, 12.68; found: C, 57.96; H, 5.19; N, 12.65.

#### 2.2.12. N-(morpholine-4-carbonothioyl)-2-oxo-2H-chromene-3-carboxamide (e9)

Light yellow powder, 50% yield; mp. 123–125 °C; IR: 3035, 2991, 1714, 1607, 1571, 1428, 1240, 1107, 991, 747, 564 cm^−1^; ^1^H NMR (CDCl_3_, 300 MHz) *δ*/ppm: 3.41 (2H, *t*, *J* = 4.9 Hz), 3.72 (2H, *t*, *J* = 4.3 Hz), 3.79 (4H, *s*), 7.28–7.88 (2H, *m*), 7.54–7.64 (2H, *m*), 7.97 (1H, *s*); ^13^C NMR (CDCl_3_, 75 MHz) *δ*/ppm:, 42.7, 47.8, 66.8, 66.9, 117.0, 118.4, 124.9, 125.2, 128.8, 133.3, 144.0, 154.3, 158.2, 163.7. LC-MS (*m*/*z*): 319.3 [M^+^]. Anal. Calcd. for C_15_H_14_N_2_O_4_S; C, 56.59; H, 4.43; N, 8.80; found: C, 56.55; H, 4.42; N, 8.83.

#### 2.2.13. N-((2,3-dihydro-1H-inden-2-yl)carbamothioyl)-2-oxo-2H-chromene-3- carboxamide (e10)

Yellow powder, 61% yield; mp. 181–183 °C; IR: 3300, 3048, 2953, 1703, 1655, 1606, 1524, 1363, 1201, 797, 742, 632 cm^−1^; ^1^H NMR (CDCl_3_, 300 MHz) *δ*/ppm: 2.97 (Ar-CH_2_, 2H, dd, *J* = 6.1, 16.1 Hz), 3.43 (Ar-CH_2_, 2H, dd, *J* = 7.6, 16.1 Hz), 4.86–4.94 (1H, *m*), 7.17–7.38 (4H, *m*), 7.41–7.46 (2H, *m*), 7.63–7.71 (2H, *m*), 8.92 (1H, *s*), 9.05 (1H, d, *J=* 7.0 Hz, NH); ^13^C NMR (CDCl_3_, 75 MHz) *δ*/ppm:, 40.1, 51.2, 116.8, 118.6, 118.8, 124.9, 125.0, 125.5, 127.0, 127.1, 130.0, 134.3, 141.0, 148.5, 154.6, 161.5, 161.6. LC-MS (*m*/*z*): 385.1 [M^+^]. Anal. Calcd. for C_20_H_16_N_2_O_3_S; C, 65.92; H, 4.43; N, 7.69; found: C, 65.90; H, 4.44; N, 7.68.

#### 2.2.14. 2-oxo-N-((2-(pyrrolidin-1-yl)ethyl)carbamothioyl)-2H-chromene-3-carboxamide (e11)

Cream powder, 42% yield; mp. 131–132 °C; IR: 3326, 3044, 2928, 1698, 1665, 1609, 1542, 1425, 1241, 1147, 995, 760, 642 cm^−1^; ^1^H NMR (CDCl_3_, 300 MHz) *δ*/ppm: 1.80 (4H, *s*, br), 2.59 (4H, *s*, br), 2.73 (-NH-CH_2_CH_2_N-, 3H, *t*, *J* = 6.4 Hz), 3.60–3.64 (-NH-CH_2_CH_2_N-, 2H, *q*, *J* = 6.4 Hz), 7.27–7.41 (2H, *m*), 7.63–7.70 (2H, *m*), 8.90 (1H, *s*), 9.03 (1H, *s*, NH); ^13^C NMR (CDCl_3_, 75 MHz) *δ*/ppm:, 23.8, 39.2, 54.3, 54.8, 116.8, 118.8, 125.4, 129.9, 134.1, 148.3, 154.6, 161.5, 161.7. LC-MS (*m*/*z*): 385.1 [M^+^]. Anal. Calcd. for C_17_H_19_N_3_O_3_S; C, 59.11; H, 5.54; N, 12.17; found: C, 59.10; H, 5.53; N, 12.19.

#### 2.2.15. N-((2-morpholinoethyl)carbamothioyl)-2-oxo-2H-chromene-3-carboxamide (e12)

Cream powder, 35% yield; mp. 136–138 °C; IR: 3324, 3039, 2916, 1695, 1605, 1542, 1444, 1200, 1113, 765, 644 cm^−1^; ^1^H NMR (CDCl_3_, 300 MHz) *δ*/ppm: 2.52–2.69 (6H, *m*), 3.56–3.62 (NHCH_2_-, 2H, *m*), 3.74–3.83 (-O(CH_2_)_2_, 4H, *m*), 7.36–7.47 (2H, *m*), 7.64–7.77 (2H, *m*), 8.91 (1H, *s*), 9.17 (1H, *s*, NH); ^13^C NMR (CDCl_3_, 75 MHz) *δ*/ppm: 36.8, 53.6, 56.9, 67.2, 116.8, 118.7, 118.8, 125.4, 130.0, 134.2, 148.3, 154.6, 161.5, 161.6. LC-MS (*m*/*z*): 362.3 [M^+^]. Anal. Calcd. for C_17_H_19_N_3_O_4_S; C, 56.50; H, 5.30; N, 11.63; found: C, 56.52; H, 5.32; N, 11.61.

#### 2.2.16. N-((2-(cyclohex-1-en-1-yl)ethyl)carbamothioyl)-2-oxo-2H-chromene-3-carboxamide (e13)

Yellow powder, 51% yield; mp. 142–143 °C; IR: 3349, 3049, 2926, 1703, 1650, 1610, 1527, 1452, 1243, 1160, 981, 755, 638 cm^−1^; ^1^H NMR (CDCl_3_, 300 MHz) *δ*/ppm: 1.55–1.68 (4H, *m*), 1.98–2.18 (4H, *m*), 2.25 (-NHCH_2_CH_2_-, 2H, *t*, *J* = 6.7 Hz), 3.51–3.58 (-NHCH_2_CH_2_-, 2H, *m*), 5.56 (-C = CH, 1H, *s*), 7.35–7.44 (2H, *m*), 7.63–7.73 (2H, *m*), 8.82 (1H, *s*, NH), 8.91 (1H, *s*); ^13^C NMR (CDCl_3_, 75 MHz) *δ*/ppm: 22.5, 23.0, 25.4, 28.0, 37.5, 38.2, 116.8, 118.7, 118.8, 124.3, 125.4, 129.9, 134.1, 134.4, 148.3, 154.5, 161.4, 161.5. LC-MS (*m*/*z*): 355.3 [M^+^]. Anal. Calcd. for C_19_H_20_N_2_O_3_S; C, 64.02; H, 5.66; N, 7.86; found: C, 64.05; H, 5.61; N, 7.87.

#### 2.2.17. N-((3,4-dimethoxyphenethyl)carbamothioyl)-2-oxo-2H-chromene-3-carboxamide (e14)

Yellow powder, 60% yield; mp. 148–149 °C; IR: 3342, 3051, 2934, 1703, 1655, 1607, 1513, 1451, 1234, 1157, 1029, 747, 641 cm^−1^; ^1^H NMR (CDCl_3_, 300 MHz) *δ*/ppm: 2.89 (-NHCH_2_CH_2_-, 2H, *t*, *J* = 7.3 Hz), 3.67–3.74 (-NHCH_2_CH_2_-, 2H, *m*), 3.86 (-OCH_3_, 3H, *s*), 3.90 (-OCH3, 3H, *s*), 6.79–6.82 (3H, *m*), 7.36–7.42 (2H, *m*), 7.64–7.71 (2H, *m*), 8.89 (1H, *s*, NH), 8.91 (1H, *s*); ^13^C NMR (CDCl_3_, 75 MHz) *δ*/ppm: 35.4, 41.7, 56.0, 111.4, 112.0, 116.8, 118.5, 118.8, 120.9, 125.5, 130.0, 131.5, 134.2, 147.8, 148.4, 149.1, 154.5, 161.5, 161.6. LC-MS (*m*/*z*): 411.3 [M^+^]. Anal. Calcd. for C_21_H_20_N_2_O_5_S; C, 61.15; H, 4.89; N, 6.79; found: C, 61.12; H, 4.90; N, 6.76.

#### 2.2.18. N-((benzo[d][1,3]dioxol-5-ylmethyl)carbamothioyl)-2-oxo-2H-chromene-3-carboxamide (e15)

Yellow powder, 66% yield; mp. 188–190 °C; IR: 3352, 3051, 2904, 1706, 1659, 1608, 1498, 1441, 1281, 1237, 1038, 925, 758, 645 cm^−1^; ^1^H NMR (CDCl_3_, 300 MHz) *δ*/ppm: 4.56 (-NHCH_2_-, 2H, d, *J* = 5.8 Hz), 5.95 (-OCH_2_O-, 2H, *s*), 6.76–6.85 (3H, *m*), 7.36–7.43 (2H, *m*), 7.65–7.72 (2H, *m*), 8.95 (1H, *s*), 9.12 (1H, *s*, NH); ^13^C NMR (CDCl_3_, 75 MHz) *δ*/ppm: 43.9, 101.3, 108.5, 108.6, 116.8, 118.5, 118.8, 121.3, 123.4, 125.5, 130.0, 131.9, 134.3, 147.1, 148.1, 148.8, 154.6, 161.6. LC-MS (*m*/*z*): 381.2 [M^+^]. Anal. Calcd. for C_19_H_14_N_2_O_5_S; C, 59.68; H, 3.69; N, 7.33; found: C, 59.65; H, 3.66; N, 7.35.

#### 2.2.19. N-(morpholinocarbamothioyl)-2-oxo-2H-chromene-3-carboxamide (e16)

Yellow powder, 25% yield; mp. 180–182 °C; IR: 3293, 3233, 3039, 2987, 1709, 1607, 1528, 1453, 1231, 1107, 865, 761 cm^−1^; ^1^H NMR (CDCl_3_, 300 MHz) *δ*/ppm: 2.99–3.08 (-N(CH_2_)_2_, 4H, *m*), 3.80–3.91 (-O(CH_2_)_2_, 4H, *m*), 7.38–7.48 (2H, *m*), 7.67–7.78 (2H, *m*), 8.96 (1H, *s*), 9.66 (-N=C-SH, 1H, *s*, SH); ^13^C NMR (CDCl_3_, 75 MHz) *δ*/ppm: 55.0, 56.0, 66.3, 66.5, 116.5, 118.3, 126.0, 130.0, 131.9, 134.5, 149.3, 154.5, 159.3, 161.1, 177.1. LC-MS (*m*/*z*): 332.2 [M^+^]. Anal. Calcd. for C_15_H_15_N_3_O_4_S; C, 54.04; H, 4.54; N, 12.60; found: C, 54.07; H, 4.52; N, 12.62.

#### 2.2.20. N-((4-methylpiperazin-1-yl)carbamothioyl)-2-oxo-2H-chromene-3-carboxamide (e17)

Yellow powder, 38% yield; mp. 204 °C; IR:; IR: 3205, 3040, 2942, 1692, 1661, 1607, 1506, 1199, 1122, 792, 551 cm^−1^; ^1^H NMR (CDCl_3_, 300 MHz) *δ*/ppm: 2.37 (-N-CH_3_, 3H, *s*), 2.65 (4H, *s*, br), 3.10 (4H, *s*, br), 7.43–7.48 (2H, *m*), 7.74–7.81 (2H, *m*), 8.94 (1H, *s*), 11.38 (1H, *s*, NH), 11.66 (-N=C-SH, 1H, *s*, SH); ^13^C NMR (CDCl_3_, 75 MHz) *δ*/ppm: 46.0, 53.9, 54.3, 116.5, 117.3, 118.4, 126.0, 130.5, 136.0, 151.2, 155.1, 160.5, 161.0, 177.0. LC-MS (*m*/*z*): 347.3 [M^+^]. Anal. Calcd. for C_16_H_18_N_4_O_3_S; C, 55.48; H, 5.24; N, 16.17; found: C, 55.45; H, 5.22; N, 16.19.

#### 2.2.21. 2-oxo-N-(piperidin-1-ylcarbamothioyl)-2H-chromene-3-carboxamide (e18)

Orange powder, 50% yield; mp. 206 °C; IR:; IR: 3133, 3047, 2945, 1694, 1607, 1480, 1228, 1190, 1034, 758, 641 cm^−1^; ^1^H NMR (CDCl_3_, 300 MHz) *δ*/ppm: 1.50–1.52 (2H, *m*), 1.74–1.81 (4H, *m*), 2.99 (4H, *s*, br), 7.41–7.47 (2H, *m*), 7.72–7.78 (2H, *m*), 8.91 (1H, *s*), 11.32 (1H, *s*, NH), 11.61 (-N=C-SH, 1H, *s*, SH); ^13^C NMR (CDCl_3_, 75 MHz) *δ*/ppm: 23.4, 25.6, 55.1, 55.8, 116.8, 119.0, 119.6, 126.2, 131.5, 135.5, 150.4, 154.8, 159.8, 161.1, 176.4. LC-MS (*m*/*z*): 330.3 [M^+^]. Anal. Calcd. for C_16_H_17_N_3_O_3_S; C, 57.99; H, 5.17; N, 12.68; found: C, 57.97; H, 5.19; N, 12.65.

#### 2.2.22. 2-oxo-N-((2-(piperazin-1-yl)ethyl)carbamothioyl)-2H-chromene-3-carboxamide (e19)

Yellow powder, 32% yield; mp.163–165 °C; IR:; IR: 3205, 3036, 2928, 1695, 1607, 1449, 1225, 1174, 1034, 790, 632 cm^−1^; ^1^H NMR (CDCl_3_, 300 MHz) *δ*/ppm: 2.58–2.78 (6H, *m*), 3.46 (2H, *t*, *J* = 4.6 Hz), 3.57–3.62 (2H, *q*, *J* = 5.8 Hz), 3.85 (2H, *s*, br), 7.24–7.42 (2H, *m*), 7.54–7.71 (2H, *m*), 7.91 (1H, *s*), 8.90 (1H, *s*, NH), 9.18 (1H, *s*, NH); ^13^C NMR (CDCl_3_, 75 MHz) *δ*/ppm: 37.0, 42.4, 47.5, 52.4, 53.0, 56.3, 116.8, 118.5, 125.1, 128.7, 130.0, 134.2, 143.2, 148.4, 154.3, 161.5, 163.5. LC-MS (*m/z*): 361.3 [M^+^]. Anal. Calcd. for C_17_H_20_N_4_O_3_S; C, 56.65; H, 5.59; N, 15.54; found: C, 56.63; H, 5.57; N, 15.55.

#### 2.2.23. N-((3-(dimethylamino)propyl)carbamothioyl)-2-oxo-2H-chromene-3-carboxamide (e20)

Yellow powder, 42% yield; mp. 100 °C; IR:; IR: 3341, 3054, 2974, 1704, 1657, 1612, 1451, 1244, 1080, 966, 757, 637 cm^−1^; ^1^H NMR (CDCl_3_, 300 MHz) *δ*/ppm: 1.77–1.84 (2H, *m*), 2.25 (-N(CH_3_)_2_, 6H, *s*), 2.34–2.41 (2H, *m*), 3.50–3.56 (2H, *m*), 7.35–7.41 (2H, *m*), 7.63–7.71 (2H, *m*), 8.90 (1H, *s*), 9.08 (1H, *s*, NH); ^13^C NMR (CDCl_3_, 75 MHz) *δ*/ppm: 27.3, 38.7, 45.6, 57.6, 116.8, 116.8, 118.8X2, 125.4, 129.9, 134.1, 148.3, 154.6, 161.5, 161.6. LC-MS (*m*/*z*): 335.3 [M^+^]. Anal. Calcd. for C_16_H_19_N_3_O_3_S; C, 57.64; H, 5.74; N, 12.60; found: C, 57.61; H, 5.76; N, 12.61.

### 2.3. CA inhibition assays

An SX.18 MV-R Applied Photophysics (Oxford, UK) stopped-flow instrument has been used to assay the catalytic/inhibition of various CA isozymes^51^. Phenol Red (at a concentration of 0.2 mM) has been used as indicator, working at the absorbance maximum of 557 nm, with 10 mM Hepes (pH 7.4) as buffer, 0.1 M Na_2_SO_4_ or NaClO_4_ (for maintaining constant the ionic strength; these anions are not inhibitory in the used concentration)^26^, following the CA-catalysed CO_2_ hydration reaction for a period of 5–10 s. Saturated CO_2_ solutions in water at 25 °C were used as substrate. Stock solutions of inhibitors were prepared at a concentration of 10 mM (in DMSO-water 1:1, v/v) and dilutions up to 1 nM done with the assay buffer mentioned above. At least seven different inhibitor concentrations have been used for measuring the inhibition constant. Inhibitor and enzyme solutions were pre-incubated together for 6 h at 4 °C prior to assay, in order to allow for the formation of the E-I complex. Triplicate experiments were done for each inhibitor concentration, and the values reported throughout the paper are the mean of such results. The inhibition constants were obtained by non-linear least-squares methods using the Cheng–Prusoff equation, as reported earlier^39^ and represent the mean from at least three different determinations. All CA isozymes used here were recombinant proteins obtained as reported earlier by our group^27^^,^^32^^,^^40^^,^^52^.

### 2.4. Molecular modeling

Molecular modeling approaches such as molecular docking simulations were performed in terms of examination and comprehension of the details in the inhibitory profiles of these molecules. Binding poses of studied compounds at the binding pockets of the proteins were determined via molecular docking processes. The 3D crystal structures of the hCA I, II, VII and IX were obtained from Protein Data Bank with the corresponding IDs of 2FW4, 5AML, 3MDZ and 3IAI, respectively. Ligand molecules were two dimensionally sketched in Maestro package of Schrodinger Small-Molecule Drug Discovery Suite^53^ and were prepared via LigPrep^54^ module of Maestro to establish the conformations with the lowest energy in physiological pH 7.4. The three-dimensional structures of the proteins are also prepared for docking via Protein Preparation Wizard module of Maestro. Grid map generation and flexible molecular docking simulations of ligands to these four proteins were implemented using Glide module^55^ and Glide/HTVS (high-throughput virtual screening), Glide/SP (standard precision), Glide/XP (extra precision), QPLD (Quantum Mechanics-Polarised Ligand Docking) and IFD (Induced Fit Docking) protocols of Maestro as well as CCDC GOLD^56^ Docking program. As the charge polarisation that induced by the active site of the protein environment is considered, quantum mechanics (QM) modeling may give the highest level of docking accuracy. For these reasons, QPLD is also considered which uses *ab initio* charge calculations. Initially, Glide/SP docking was carried out to generate five poses per docked compound. These poses were submitted to QM charge calculations, which uses the 6–31 G*/LACVP* basis set, B3LYP density functional, and “Ultrafine” SCF accuracy level. In GOLD algorithm, consensus docking protocol was used to generate protein–ligand complexes with GOLD 5.3.0 software. In this respect, two docking scoring functions were combined: GoldScore and ChemScore. In this study, default genetic algorithm parameters were used and 20 poses were generated for each ligand. Search efficiency was set to its maximum value (200%) in order to increase the reliability of the docking results. Flexible amino acid side chains/rotatable groups involved in binding pocket were selected separately for all isoforms according to their protein–ligand interaction maps available in PDB. Ligand molecules were also set as flexible during all molecular docking calculations.

## 3. Result and discussion

### 3.1. Chemistry

**Scheme 1.**
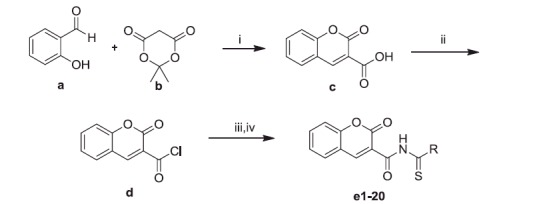
Synthesis of new thiourea substituted coumaryl-carboxamid derivatives. Reaction conditions: (i) H_2_O, reflux, 10 h; (ii) SOCl_2_, 80 °C, 4 h; (iii) KSCN, CH_3_CN, 70 °C, 30 min.; (iv) RNH_2_, 70 °C, 4 h.

The syntheses of the target compounds **e1**–**e20** are depicted in Scheme 1. 3-Coumarin carboxylic acid (**c**) was synthesized from salicylaldehyde (**a**) according to literature procedures^38^ and it was converted to the acyl chloride by using SOCl_2_. To obtain thiourea-substituted coumaryl-carboxamid derivatives (**e1**–**e20**), 2-oxo-2H-chromene-3-carbonyl chloride (**d**) was reacted with KSCN and various amines in CH_3_CN, respectively.

All the new compounds were characterized by ^1^H NMR, ^13^C NMR, IR, MS and elemental analysis. In the IR spectra of the synthesized compounds, it was possible to observe the absorptions about 3300 cm^−1^ relating to NH stretch of thiourea groups, about 1650 cm^−1^ relating to C=O stretch for thiourea, absorptions in about 1710 cm^−1^ from coumarin carbonyl moiety stretch. From the ^1^H NMR spectra, the signals for aromatic hydrogens were observed between 7.17 and 7.77 ppm, the signal of NH proton at thiourea was detected at about 8.90 ppm and signals observed about 11.3 ppm for SH proton at the resonance due to thiourea groups (N=C–SH). In addition, the signals of aliphatic hydrogen atoms were determined between 1.00–4.50 ppm. From the ^13^C NMR spectra, the signals can be seen about 177 and 163 ppm for C–SH and carbonyl of thiourea groups, respectively. The signals of the aliphatic and aromatic carbons were observed at 20–50 ppm and 110–158 ppm, respectively.

^1^H NMR, ^13^C NMR and MS spectra of the synthesized compounds are given in supplementary materials.

### 3.2. CA inhibition

The inhibition constants (*K*_i_) of the synthesized compounds **e1**–**e20** against hCA I, hCA II, hCA VII and hCA IX isoforms are given in Table 1. The hCA I, II and VII isoforms for all compounds were investigated here in the micromolar range. On the other hand, the tumour-associated isoform hCA IX was selectively inhibited by all investigated compounds with inhibition constants ranging between 107.9 and 2589.4 nM. Compound **e11** showed the strongest inhibition against hCA IX with a *K*_i_ of 107.9 nM. Furthermore, the hCA IX inhibitory activity of **e5**, **e8** and **e10** are close to that of **e11** (*K*_i_=115.1 nM, 128.1 nM and 130.3 nM, respectively).

**Table 1.** Carbonic anhydrase inhibitions of synthesized thiourea substituted coumaryl-carboxamid derivatives.
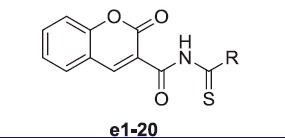

		*K*_i_ (nM)*			
Compound	*R*	hCA I	hCA II	hCA VII	hCA IX
**e1**		>10,000	>10,000	>10,000	322.9
**e2**		>10,000	>10,000	>10,000	286.4
**e3**		>10,000	>10,000	>10,000	376.2
**e4**		>10,000	>10,000	>10,000	351.4
**e5**		>10,000	>10,000	>10,000	115.1
**e6**		>10,000	>10,000	>10,000	297.5
**e7**		>10,000	>10,000	>10,000	201.8
**e8**		>10,000	>10,000	>10,000	128.1
**e9**		>10,000	>10,000	>10,000	136.5
**e10**		>10,000	>10,000	>10,000	130.3
**e11**		>10,000	>10,000	>10,000	107.9
**e12**		>10,000	>10,000	>10,000	223.8
**e13**		>10,000	>10,000	>10,000	179.8
**e14**		>10,000	>10,000	>10,000	196.4
**e15**		>10,000	>10,000	>10,000	184.5
**e16**		>10,000	>10,000	>10,000	2589.4
**e17**		>10,000	>10,000	>10,000	258.9
**e18**		>10,000	>10,000	>10,000	387.5
**e19**		>10,000	>10,000	>10,000	249.6
**e20**		>10,000	>10,000	>10,000	182.2
**AAZ**		250	12.1	6	25.8

* Mean from three different assays, by a stopped flow technique (errors were in the range of ±5–10% of the reported values).

The following structure–activity relationship (SAR) observations can be drawn from data of Table 1: (i) Replacing the methyl group on the NH of thiourea moiety (**e1**, *K*_i_=322.9 nM) by a propyl (**e2**, *K*_i_=286.4 nM), a cyclohexyl (**e5**, *K*_i_=115.1 nM) and a 2,3-dihydro-indenyl ring (**e10**, *K*_i_=130.3 nM) led to an increase in the inhibitory activity against hCA IX; on the other hand, the binding of a second alkyl group to -*N* atom (*N*,*N*-diethyl (**e3**, *K*_i_ = 376.2 nM) and *N*,*N*-diisopropyl (**e4**, *K*_i_ = 351.4 nM)) diminished the inhibitory activity against hCA IX. (ii) The expansion of the pyrrolidine ring of compound **e6** (*K*_i_ = 297.5 nM against hCA IX) to a piperidine (compound **e7**, *K*_i_ = 201.8 nM against hCA IX) increased the inhibitory activity against hCA IX. Additionally, incorporated N and O atoms into the piperidine ring (R  =  4-methylpiperazine (**e8**) and R  =  morpholine (**e9**), *K*_i_ = 128.1 nM and 136.5 nM, respectively, against hCA IX) caused a greater increase in the inhibitory activity against hCA IX. (iii) The presence of an ethyleneamine group as a spacer between the thionyl moiety and the pyrrolidine ring positively affected the inhibitory activity against hCA IX (comparing **e6** (*K*_i_ = 297.5 nM) with **e11** (*K*_i_ = 107.9 nM)) and the presence of a propyleneamine group between the thionyl and the *N,N*-dialkyl moieties did likewise (**e20**, *K*_i_ = 182.2 nM). On the contrary, the ethyleneamine group between the thionyl moiety and both the piperazine and morpholine rings decreased the inhibitory activity against hCA IX (comparing **e8** (*K*_i_ = 128.1 nM) with **e19** (*K*_i_ = 249.6 nm) and comparing **e9** (*K*_i_ = 136.5 nm) with **e12** (*K*_i_ = 223.8 nM)). (iv) Similarly, the presence of an amine group (-NH-) between the thionyl moiety and the piperidine, piperazine or morpholine ring led to a major decline the inhibitory activity against hCA IX (comparing **e7** (*K*_i_ = 201.8 nM) with **e18** (*K*_i_ = 387.5 nM), comparing **e8** (*K*_i_ = 128.1 nM) with **e17** (*K*_i_ = 258.9 nM) and comparing **e9** (*K*_i_ = 136.5 nM) with **e16** (*K*_i_ = 2589.4 nM)). (v) The replacement of the ethyleneamine group by a methyleneamine between the thionyl moiety and the aromatic ring and the cyclisation of the dimethoxy group at the phenyl ring to the dioxolane ring did not cause significant changes in the hCA IX inhibitory activity (comparing **e14** (*K*_i_ = 196.4 nM) with **e15** (*K*_i_ = 184.5 nM)).

**Figure 1.**
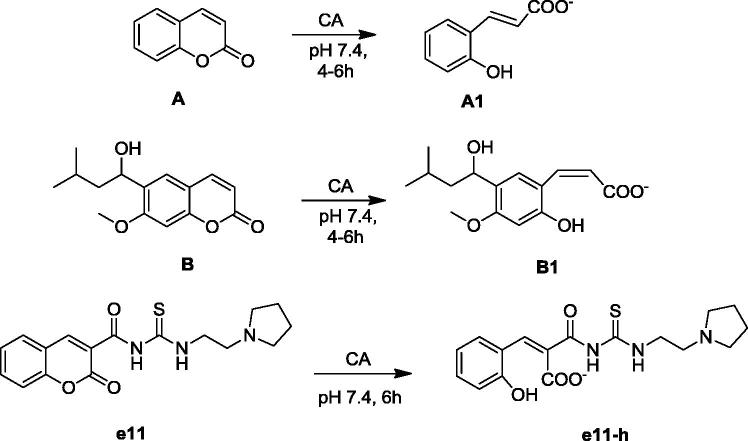
Formation of 2-hydroxy-cinnamic acids A1, B1 and **e11-h** by the CA-mediated hydrolysis of coumarin A, B and **e11**.

According to X-ray crystallographic studies, coumarins are mechanism-based inhibitors, which undergo hydrolysis under the influence of the zinc hydroxide, nucleophilically active species of the enzyme, with the generation of substituted-2-hydroxycinnamic acids (Figure 1)^26^^,^^39–41^. It was reported that coumarin/sulphocoumarin inhibitors and enzyme solutions were pre-incubated together for ∼6 h prior to assay in order to allow for the formation of the E-I complex or for the eventual active site-mediated hydrolysis of the inhibitor^42^. Based on the above consideration, we estimate that the coumarin ring should undergo ring opening by hydrolysing coumarinic moiety to cinnamic acid derivative during pre-incubation on enzyme and inhibitor (Figure 1).

### 3.3. Molecular modeling

**Figure 2.**
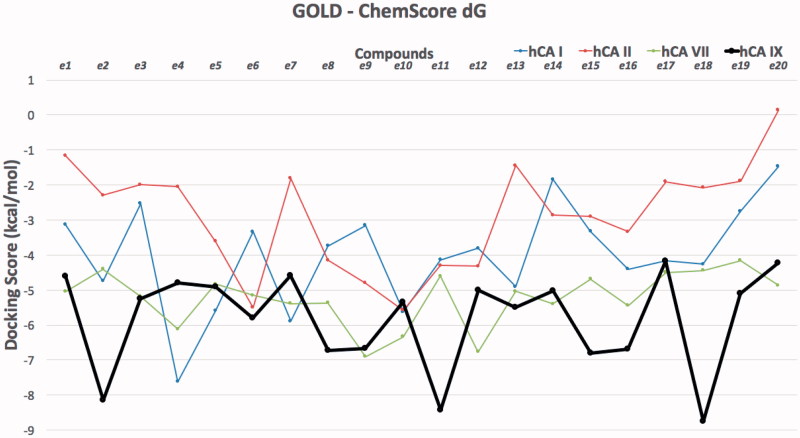
Docking scores (GOLD ChemScore dG) of studied compounds (nonhydrolyzed forms) at the hCA I, II, VII and IX isoforms.

**Figure 3.**
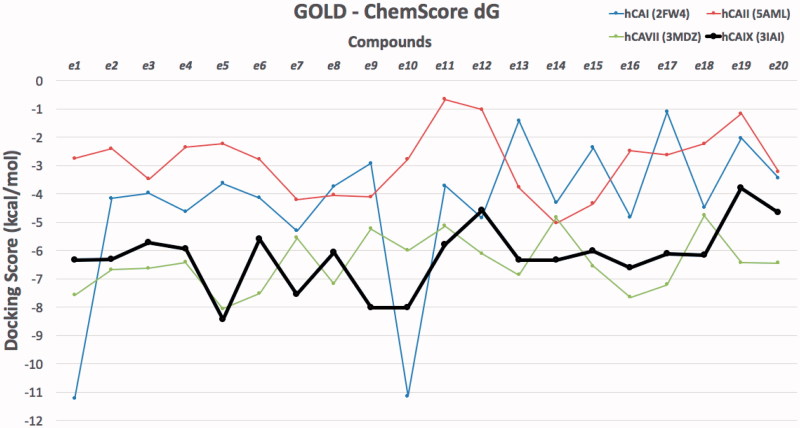
Docking scores (GOLD ChemScore dG) of studied compounds (hydrolyzed forms) at the hCA I, II, VII and IX isoforms.

Molecular modeling approaches, such as molecular docking calculations, are generally used techniques to qualify and quantify the important information about the ligand–receptor interaction analysis on atomistic level. There are many studies using these approaches integrated to human carbonic anhydrases (hCA) in order to clarify the molecular mechanism of action and bioactive conformation of proposed compounds at the binding site of the protein^43–49^. Since there is no experimental study yet on stable state (hydrolyzed/nonhydrolyzed) of studied compounds from lactone moiety at the binding pocket of the CAs, both hydrolyzed and nonhydrolyzed forms were considered at the docking. Molecular docking results were evaluated for their docking scores in the binding pocket of the hCA I, II, VII and IX isoforms. Therefore, the protein–ligand complexes with the top-docking scores were selected for further analyses. Docking results of compounds (**e1–e20**) at the binding pockets of hCA isoforms with Glide/HTVS (high-throughput virtual screening), Glide/SP (standard precision), Glide/XP (extra precision), IFD (induced fit docking), QPLD (quantum polarised ligand docking) and GOLD protocols were compared. Since GOLD docking program gave more successful results GOLD docking results were considered in further analysis (Tables 2 and 3). Docking scores of studied compounds (with hydrolyzed and nonhydrolyzed forms) at the hCA I, II, VII and IX isoforms were compared at the Figures 2 and 3.

**Table 2.** Top-docking scores of compounds **e1–e20** (non-hydrolyzed forms) at the hCAI, hCAII, hCA VII and hCA IX isoforms.

Compounds	hCA I (2FW4)	hCA II (5AML)	hCA VII (3MDZ)	hCA IX (3IAI)
**e1**	−3.128	−1.163	−5.049	−4.605
**e2**	−4.747	−2.292	−4.400	−8.142
**e3**	−2.534	−1.996	−5.177	−5.259
**e4**	−7.622	−2.049	−6.114	−4.788
**e5**	−5.600	−3.613	−4.832	−4.903
**e6**	−3.339	−5.502	−5.150	−5.796
**e7**	−5.885	−1.811	−5.383	−4.591
**e8**	−3.745	−4.148	−5.370	−6.723
**e9**	−3.159	−4.798	−6.899	−6.678
**e10**	−5.625	−5.580	−6.330	−5.345
**e11**	−4.137	−4.298	−4.604	−8.429
**e12**	−3.805	−4.322	−6.770	−4.996
**e13**	−4.907	−1.443	−5.040	−5.491
**e14**	−1.843	−2.858	−5.397	−5.027
**e15**	−3.328	−2.898	−4.689	−6.798
**e16**	−4.407	−3.338	−5.441	−6.687
**e17**	−4.172	−1.909	−4.497	−4.167
**e18**	−4.258	−2.075	−4.446	−8.750
**e19**	−2.744	−1.885	−4.158	−5.103
**e20**	−1.473	0.138	−4.866	−4.226

Used protein data bank (PDB) IDs of proteins were also highlighted at the table. Docking scores are in kcal/mol.

**Table 3.** Top-docking scores of compounds **e1**–**e20** (hydrolyzed forms) at the hCAI, hCAII, hCA VII and hCA IX isoforms.

Compounds	hCAI (2FW4)	hCAII (5AML)	hCAVII (3MDZ)	hCAIX (3IAI)
**e1**	−4.422	−5.837	−5.658	−7.025
**e2**	−4.980	−6.555	−5.468	−6.878
**e3**	−4.884	−4.292	−6.166	−7.064
**e4**	−5.270	−7.194	−4.855	−1.879
**e5**	−5.617	−5.991	−6.191	−8.287
**e6**	−4.802	−6.698	−6.690	−7.655
**e7**	−4.715	−6.375	−5.463	−6.833
**e8**	−4.441	−3.632	−5.703	−4.146
**e9**	−5.343	−6.699	−6.724	−7.310
**e10**	−4.726	−7.553	−7.104	−8.819
**e11**	−4.587	−5.816	−5.597	−9.006
**e12**	−5.377	−5.121	−7.164	−8.769
**e13**	−5.366	−6.667	−5.690	−9.481
**e14**	−5.554	−5.967	−6.518	−9.238
**e15**	−4.51	−5.879	−6.036	−9.445
**e16**	−4.929	−6.322	−6.652	−4.213
**e17**	−4.642	−5.634	−6.219	−4.123
**e18**	−5.149	−6.855	−3.487	−2.713
**e19**	−5.82	−6.421	−7.123	−9.301
**e20**	−4.507	−5.738	−6.672	−9.096

Used protein data bank (PDB) IDs of proteins were also highlighted at the table. Docking scores are in kcal/mol.

**Figure 4.**
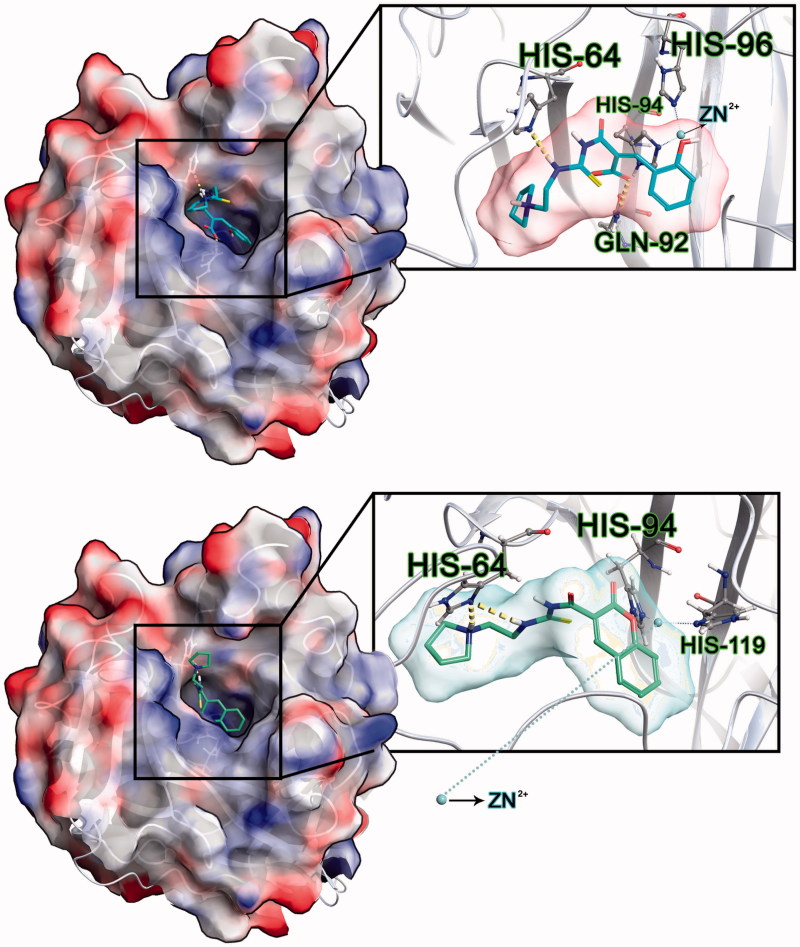
2D and 3D ligand interaction diagrams of active compound **e11** at the binding cavity of hCA IX. (top) hydrolyzed form; (bottom) nonhydrolyzed form.

The most active compound **e11** at the hCA IX showed high docking scores compared to its predicted binding energies at hCA I, II and VII isoforms in both hydrolyzed and non-hydrolyzed forms. In addition, the compounds **e2** and **e18** in their non-hydrolyzed forms were found to have high selectivity to hCA IX. Hydrolyzed forms of compounds showed higher docking scores against hCA IX (i.e. **e10**–**e15**, **e19** and **e20**). Figure 4 shows 2D and 3D ligand interaction diagrams of active compound **e11** at the binding cavity of hCA IX.

## 4. Conclusions

A series of 20 novel thiourea-substituted coumaryl-carboxamide derivatives (**e1**–**20**) were synthesized as CA inhibitors and they were evaluated for the inhibition of hCA I, II, IV and IX isoforms. All synthesized compounds exhibited selective inhibitory activity in the high nanomolar range against the tumour-associated isoform hCA IX. On the other hand, the hCA I, II and VII isoforms of were not inhibited by the investigated compounds. Multiscale molecular modeling approaches and different molecular docking algorithms were used to investigate inhibitory profiles, binding poses and predicted binding energies of studied compounds (both hydrolyzed and non-hydrolyzed forms) at the active sites of the CA I, II, VII and IX isoforms. The docking studies showed that hydrolyzed form of **e11**, which is the most active compound against hCA IX, interacted with His64, His94, His96 and Gln92 and non-hydrolyzed form of **e11** interacted with His64, His94 and His119 in the active side of hCA IX.

^1^H and ^13^C NMR and MS spectra of the synthesized compounds are given in the Supplementary Materials.

## Supplementary Material

IENZ_Supplementary_Material.pdf
