# Supplementary material for: Synthesis, biological activity and multiscale molecular modeling studies for coumaryl-carboxamide derivatives as selective carbonic anhydrase IX inhibitors
Source: J Enzyme Inhib Med Chem. 2017 Aug 4;32(1):1042–52. doi: 10.1080/14756366.2017.1354857 (PMC6009903; doi:10.1080/14756366.2017.1354857)
Supplement: IENZ_Supplementary_Material.pdf [file IENZ_A_1354857_SM1543.pdf]

Synthesis, Biological Activity and Multiscale Molecular Modeling Studies for Coumaryl-carboxamid Derivatives as Selective Carbonic Anhydrase IX Inhibitors

SUPPLEMENTARY MATERIALS

^{1}H and ^{13}C NMR and MS spectra of **c, d,** and **e1-e20**................................ 2-34

S1: ^{1} H NMR ($CDCl_3$) and ^{13}C NMR (DMSO-d_6) spectra of **c**

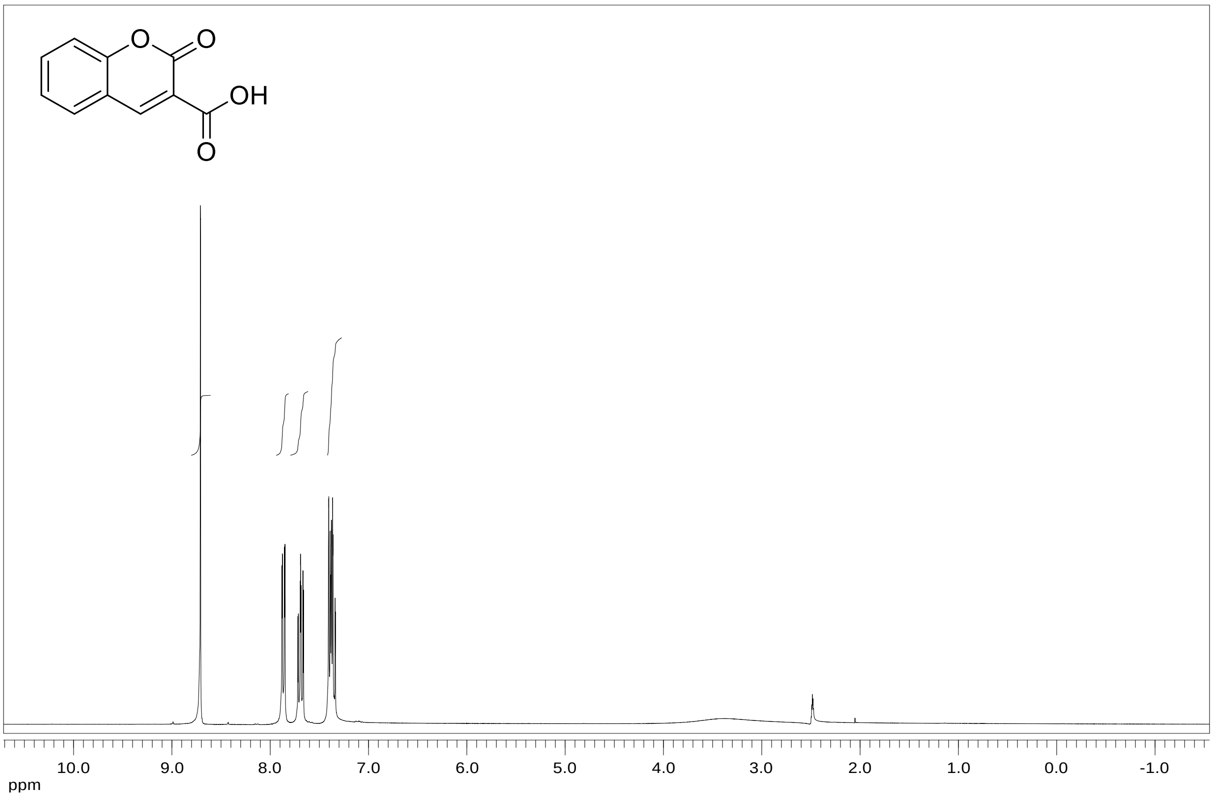

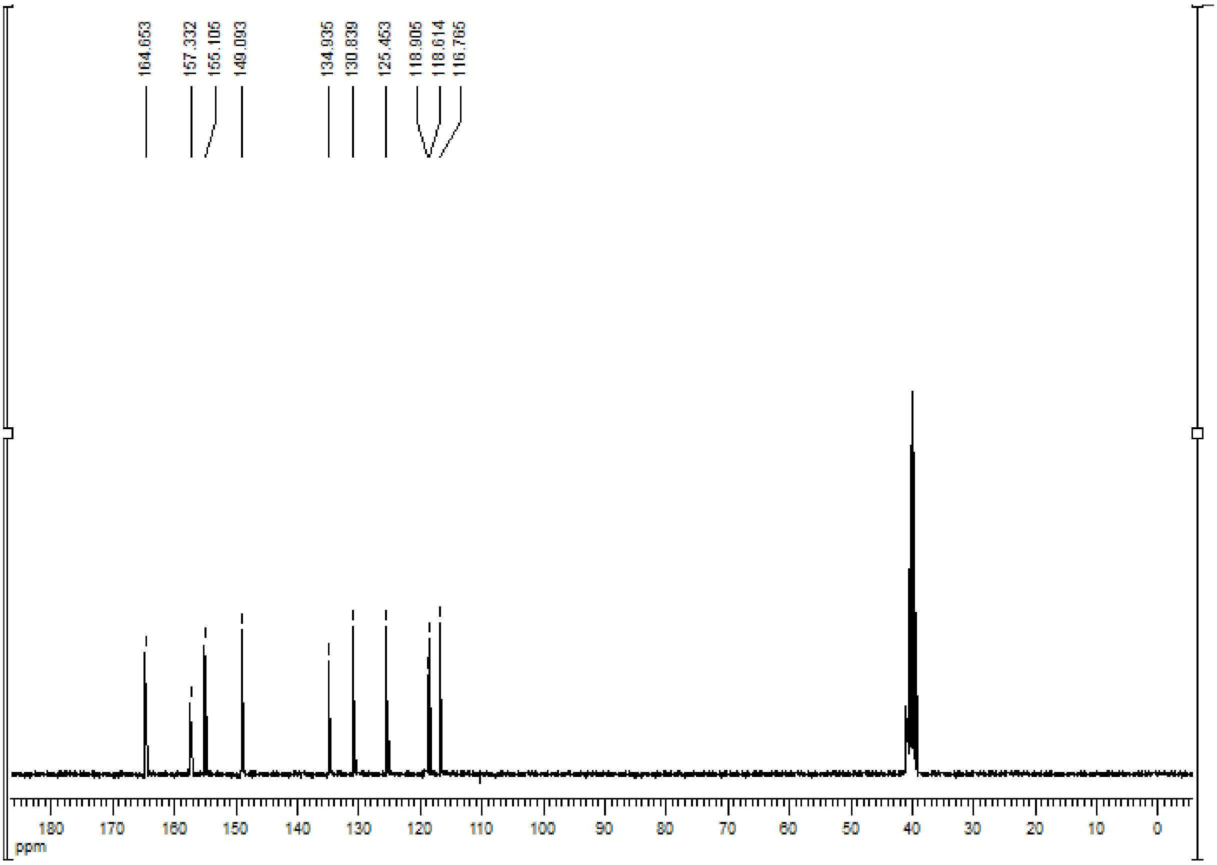

S2: [1] H NMR ($CDCl_3$) and ^{13}C NMR (DMSO-d_6) spectra of **d**

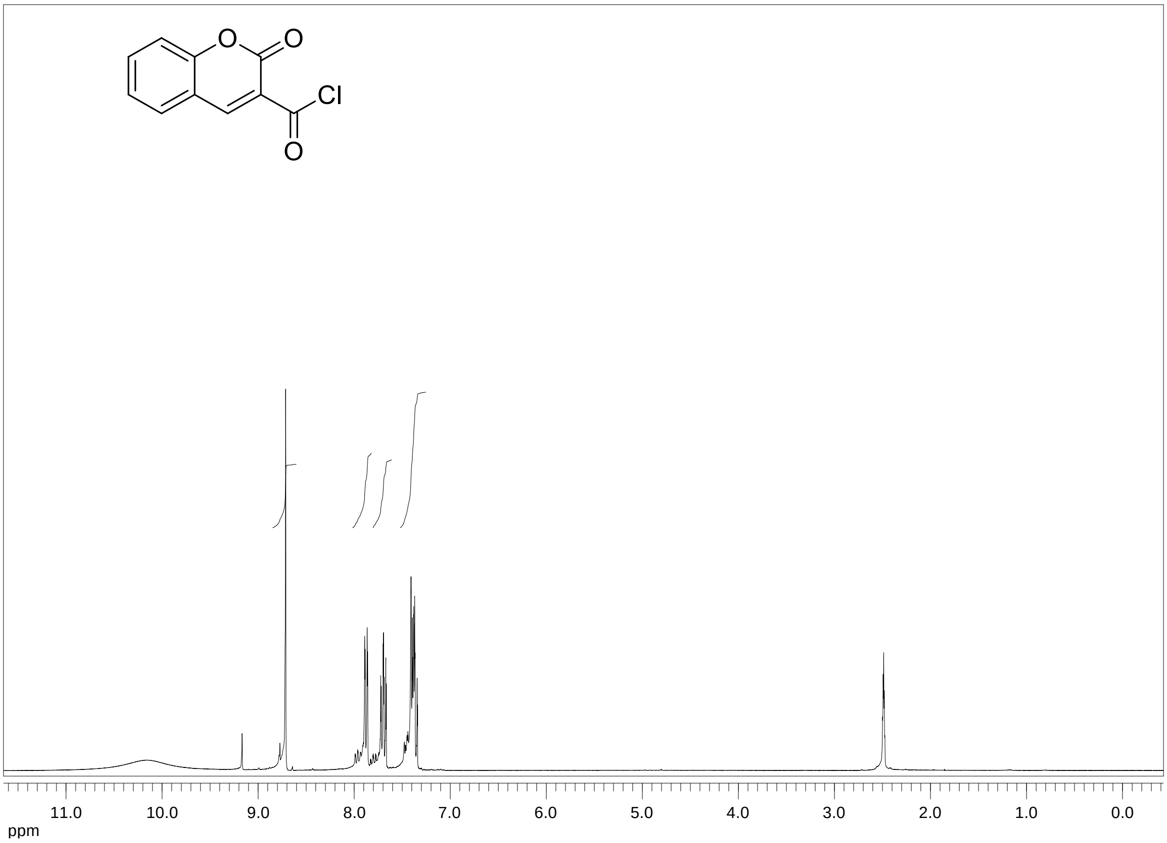

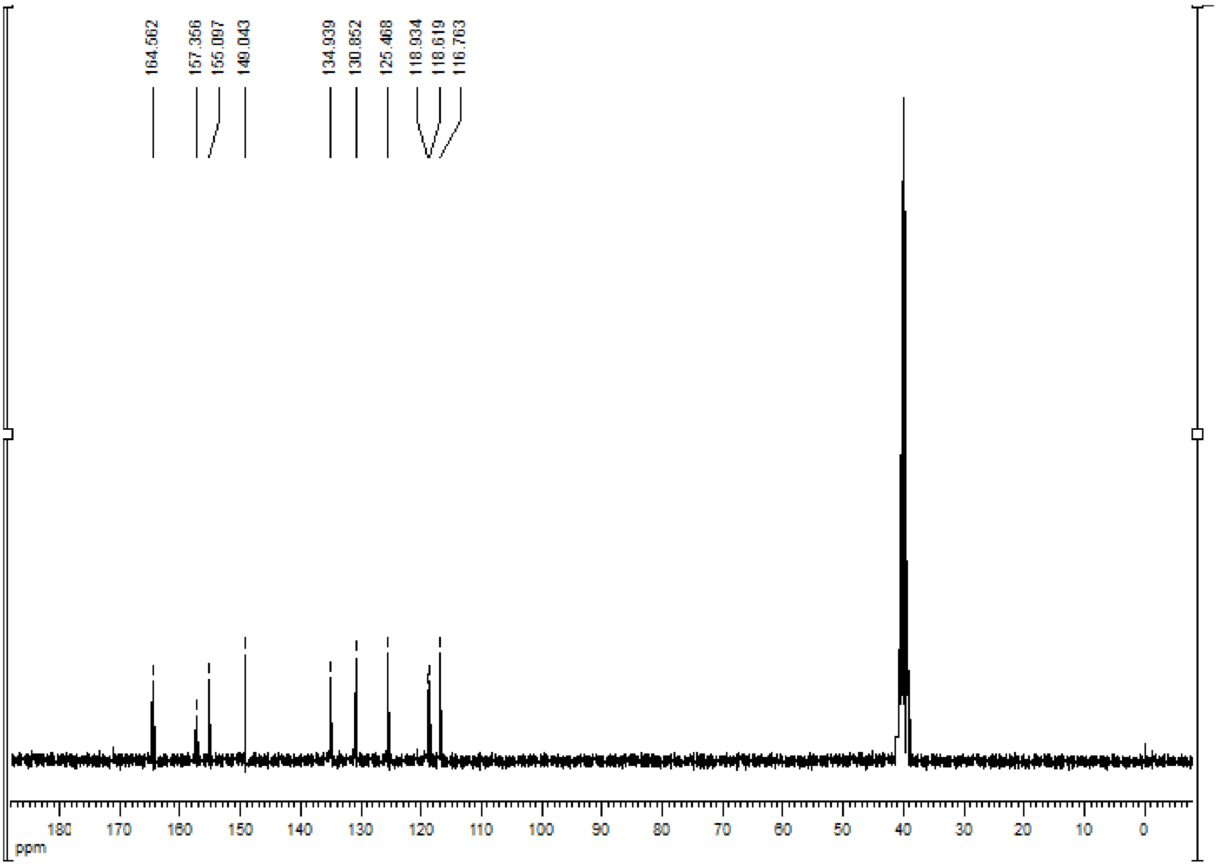

S3: [1] H NMR ($CDCl_3$), ^{13}C NMR (DMSO-d_6) and MS spectra of **e1**

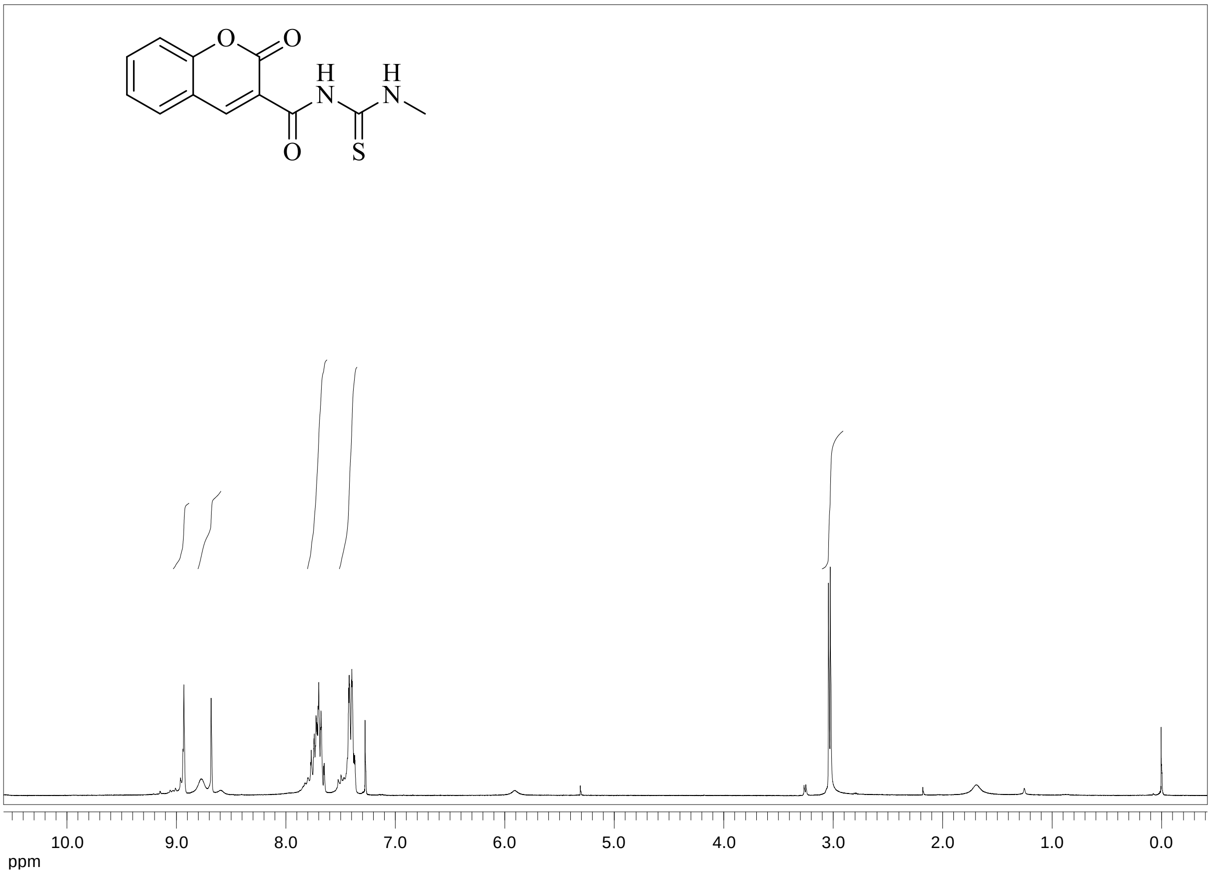
O
O
H
N
H
N
O
S
10.0
9.0
8.0
7.0
6.0
5.0
4.0
3.0
2.0
1.0
0.0
ppm

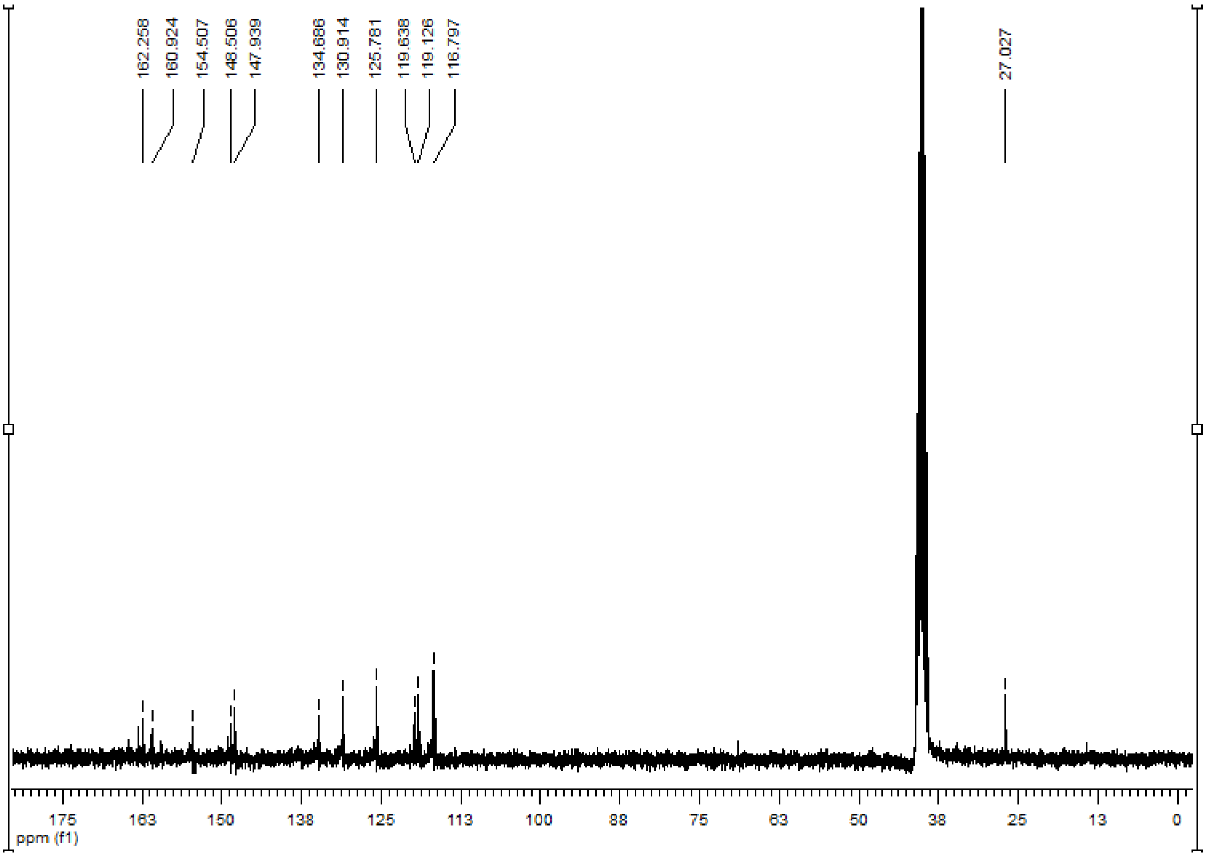
162.258
160.924
154.507
148.506
147.939
134.686
130.914
125.781
119.638
119.126
116.797
27.027
175
163
150
138
125
113
100
88
75
63
50
38
25
13
0
ppm (f1)

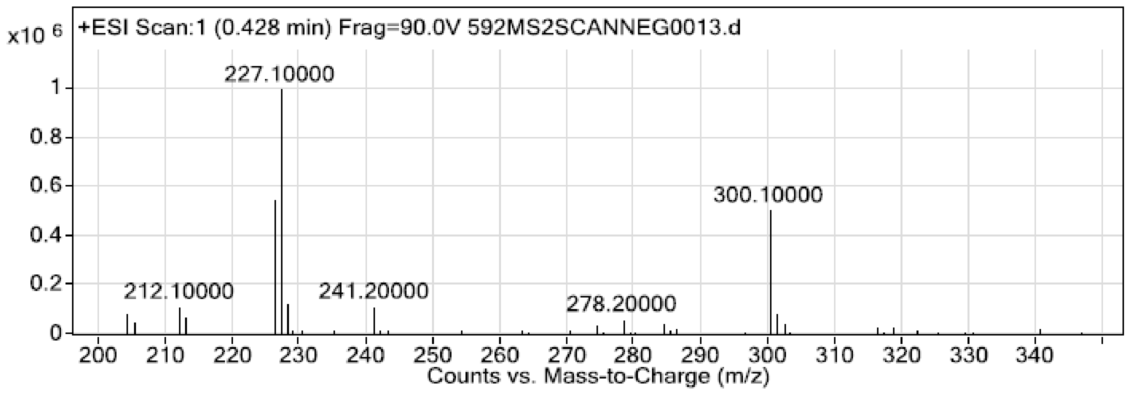

S4: ^{1}H NMR ($CDCl_3$), ^{13}C NMR ($CDCl_3$) and MS spectra of **e2**

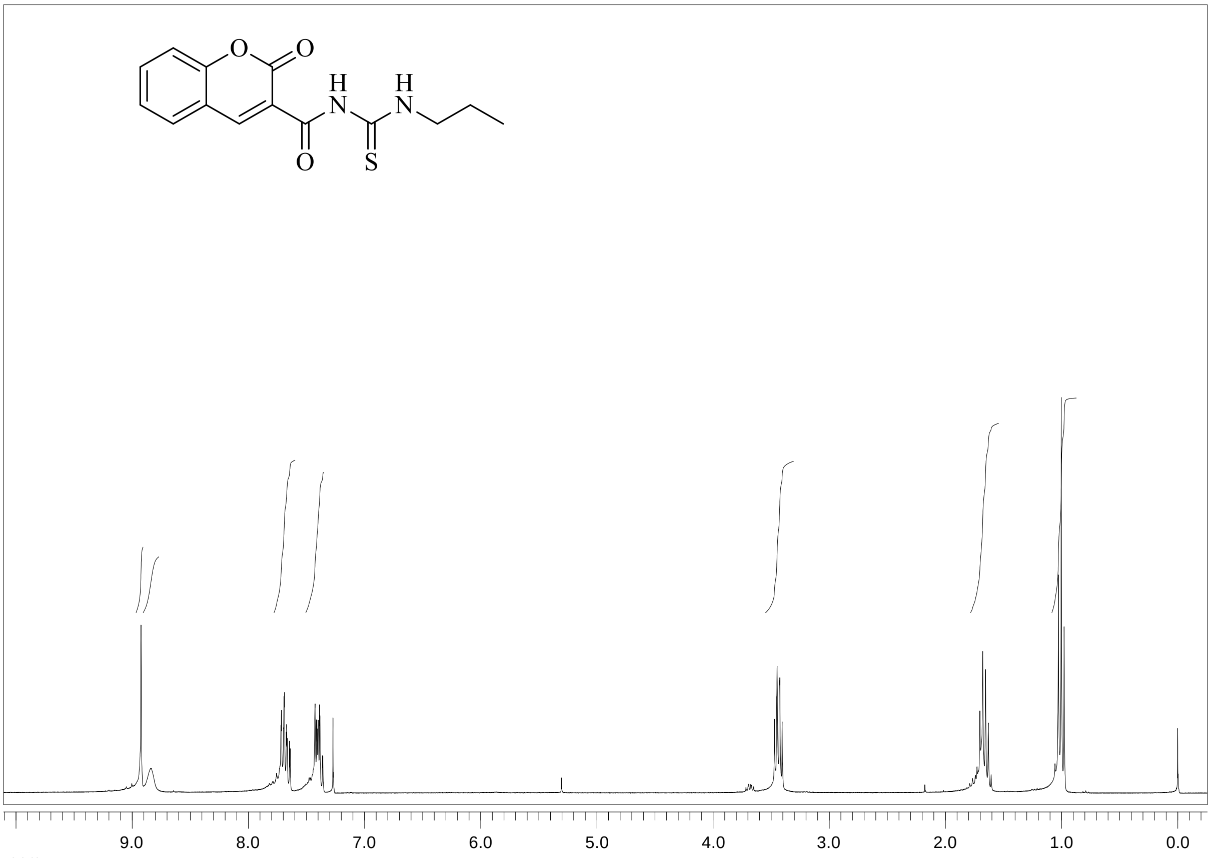

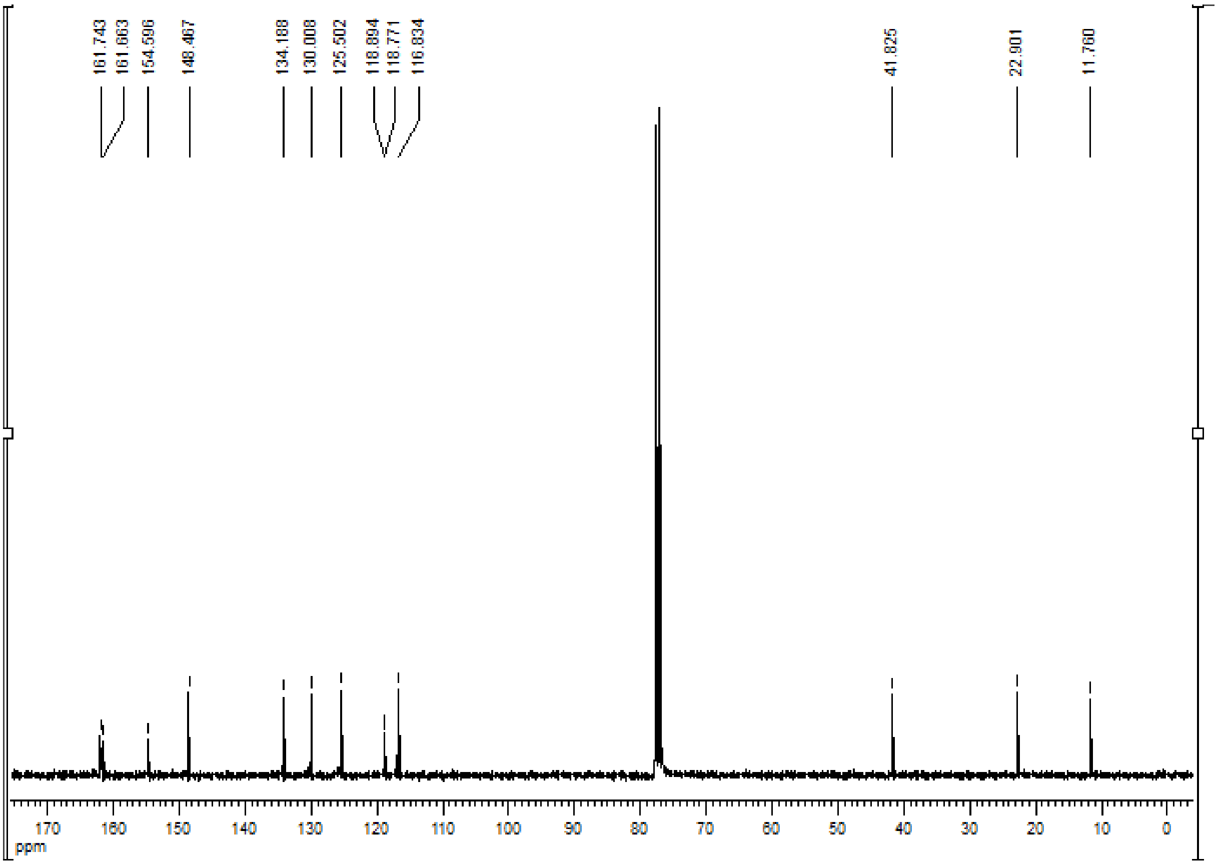
161.743
161.663
154.596
148.467
134.188
130.008
125.502
118.894
118.771
116.834
41.825
22.901
11.760
ppm

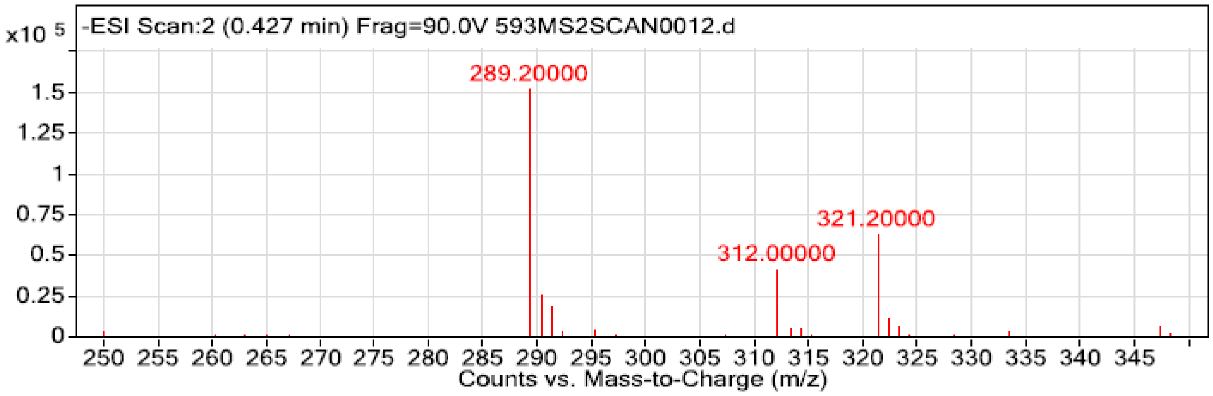
-ESI Scan:2 (0.427 min) Frag=90.0V 593MS2SCAN0012.d
x10 5
289.20000
312.00000
321.20000
Counts vs. Mass-to-Charge (m/z)

S5: [1] H NMR ($CDCl_3$), ^{13}C NMR ($CDCl_3$) and MS spectra of **e3**

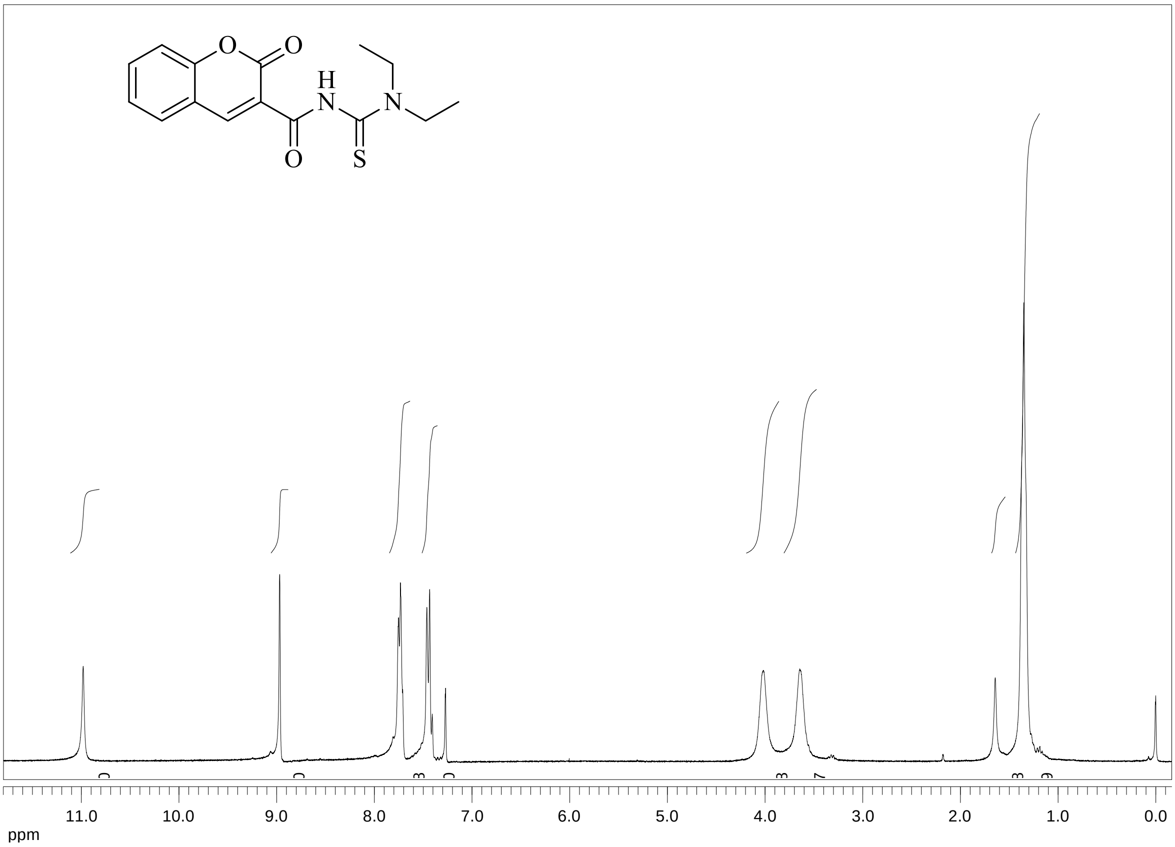

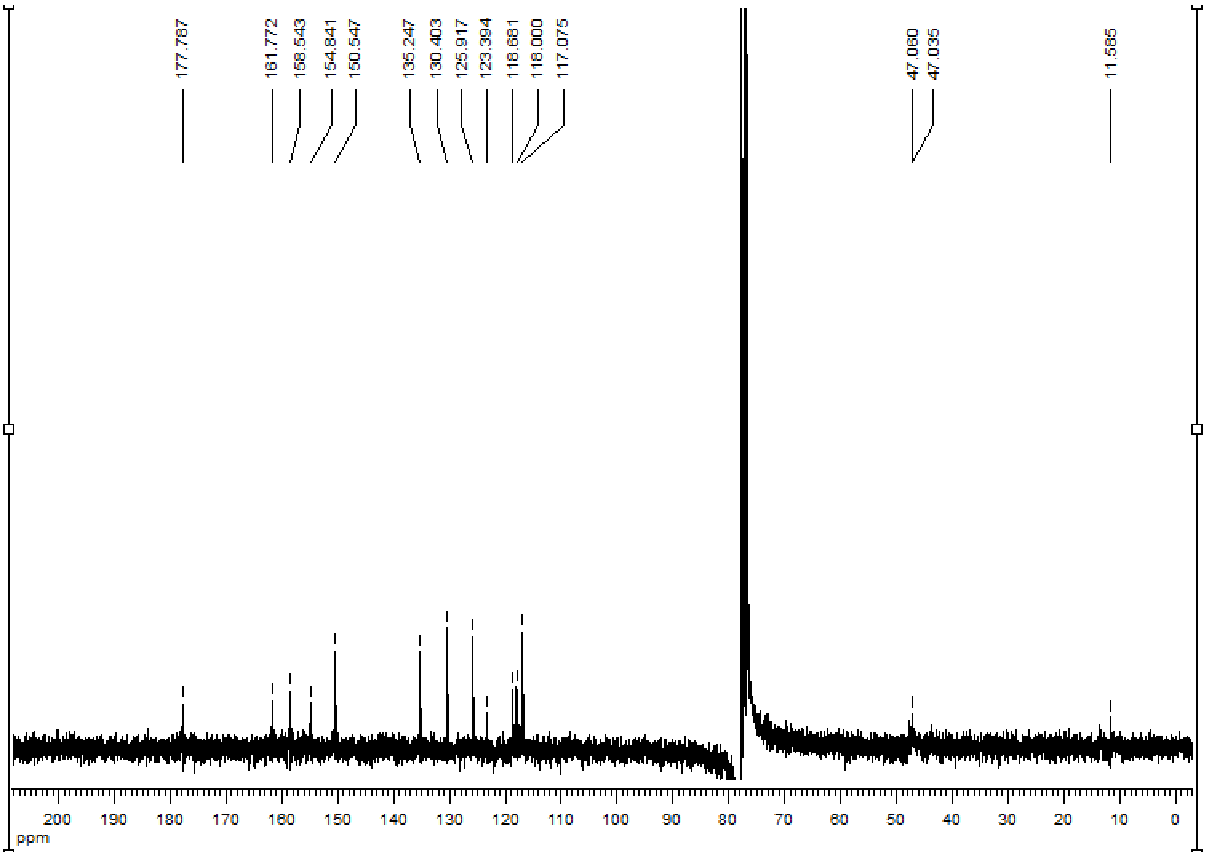

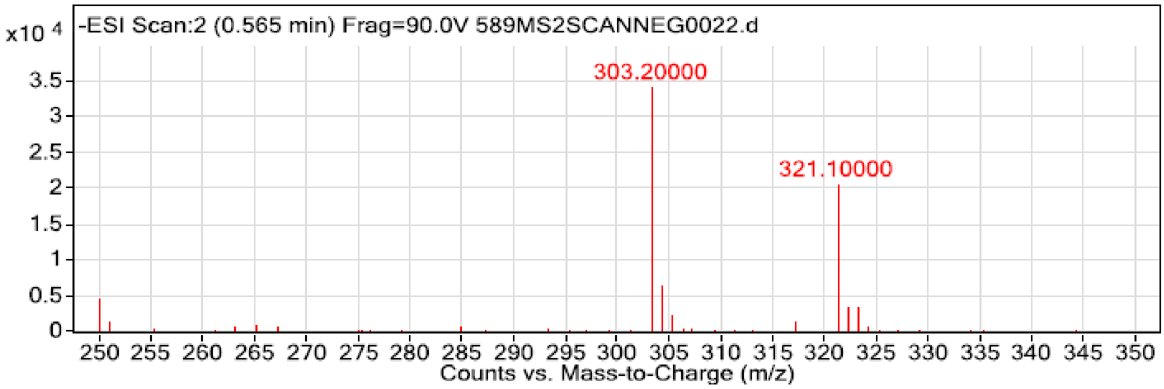

S6: ^{1}H NMR ($CDCl_3$), ^{13}C NMR ($CDCl_3$) and MS spectra of **e4**

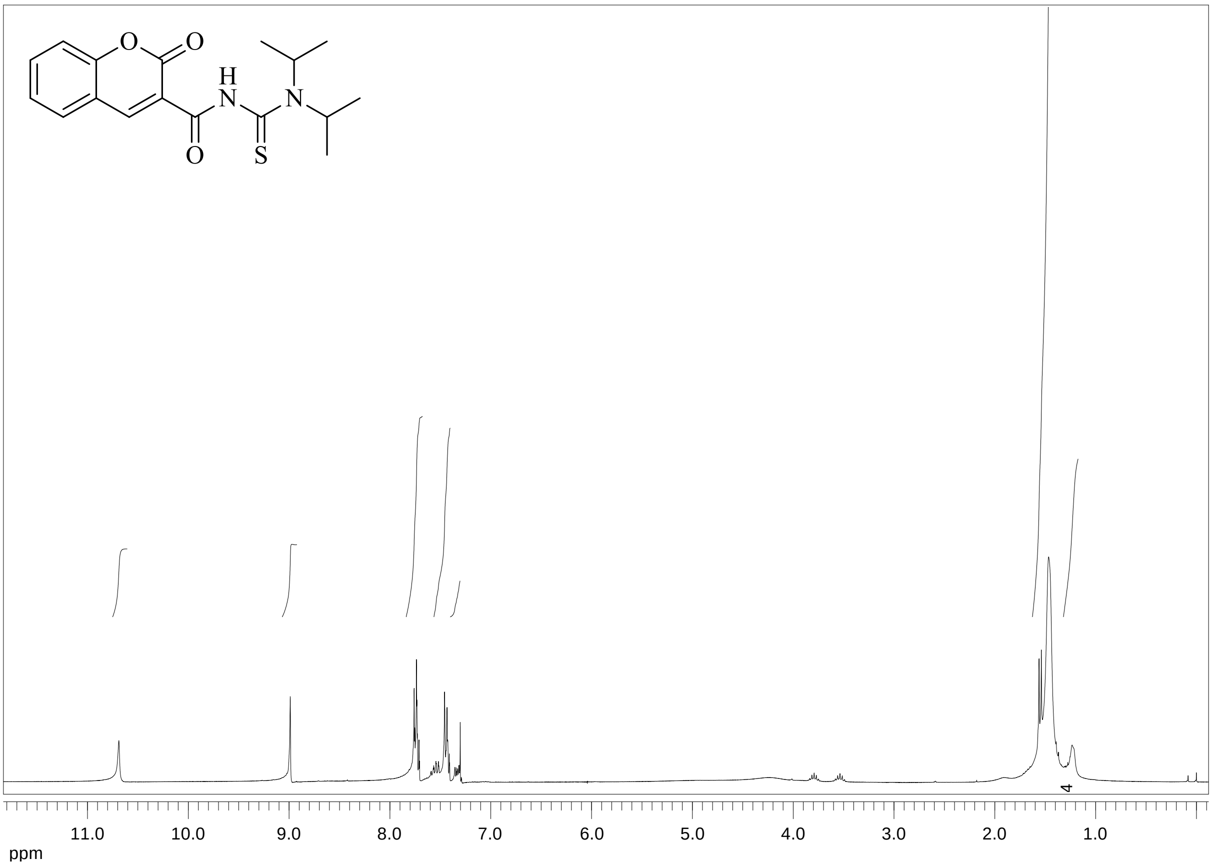

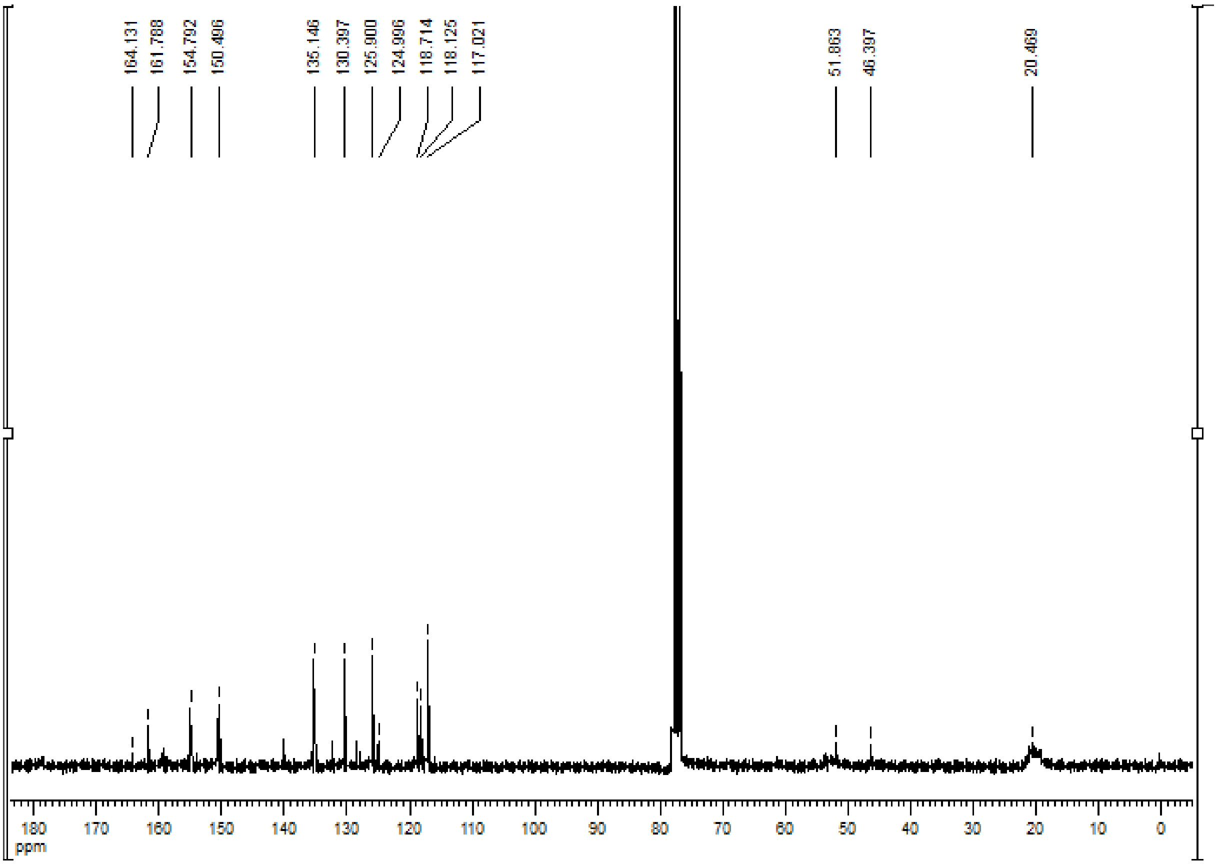
164.131
161.788
154.792
150.496
135.146
130.397
125.900
124.996
118.714
118.125
117.021
51.863
46.397
20.469
180 170 160 150 140 130 120 110 100 90 80 70 60 50 40 30 20 10 0
ppm

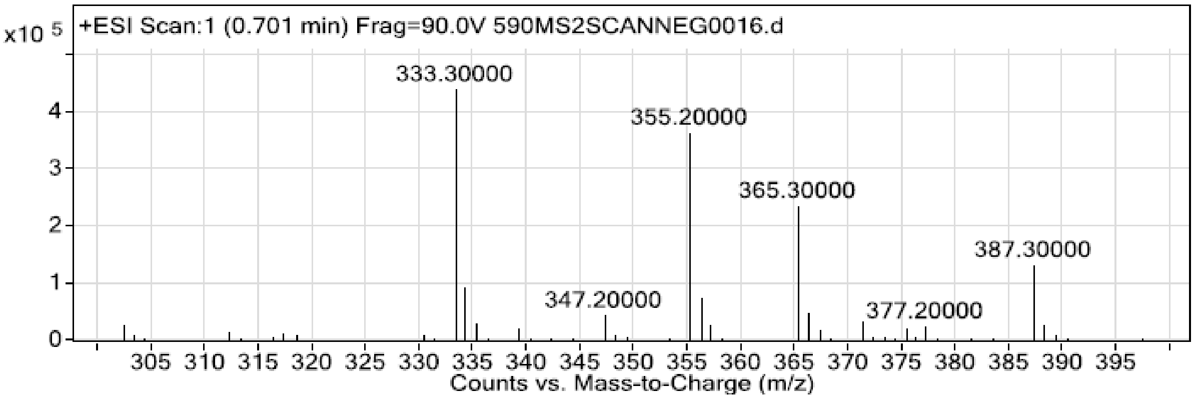
x10 5
+ESI Scan:1 (0.701 min) Frag=90.0V 590MS2SCANNEG0016.d
333.30000
355.20000
365.30000
387.30000
347.20000
377.20000
0 1 2 3 4
305 310 315 320 325 330 335 340 345 350 355 360 365 370 375 380 385 390 395
Counts vs. Mass-to-Charge (m/z)

S7: [1] H NMR ($CDCl_3$), ^{13}C NMR ($CDCl_3$) and MS spectra of **e5**

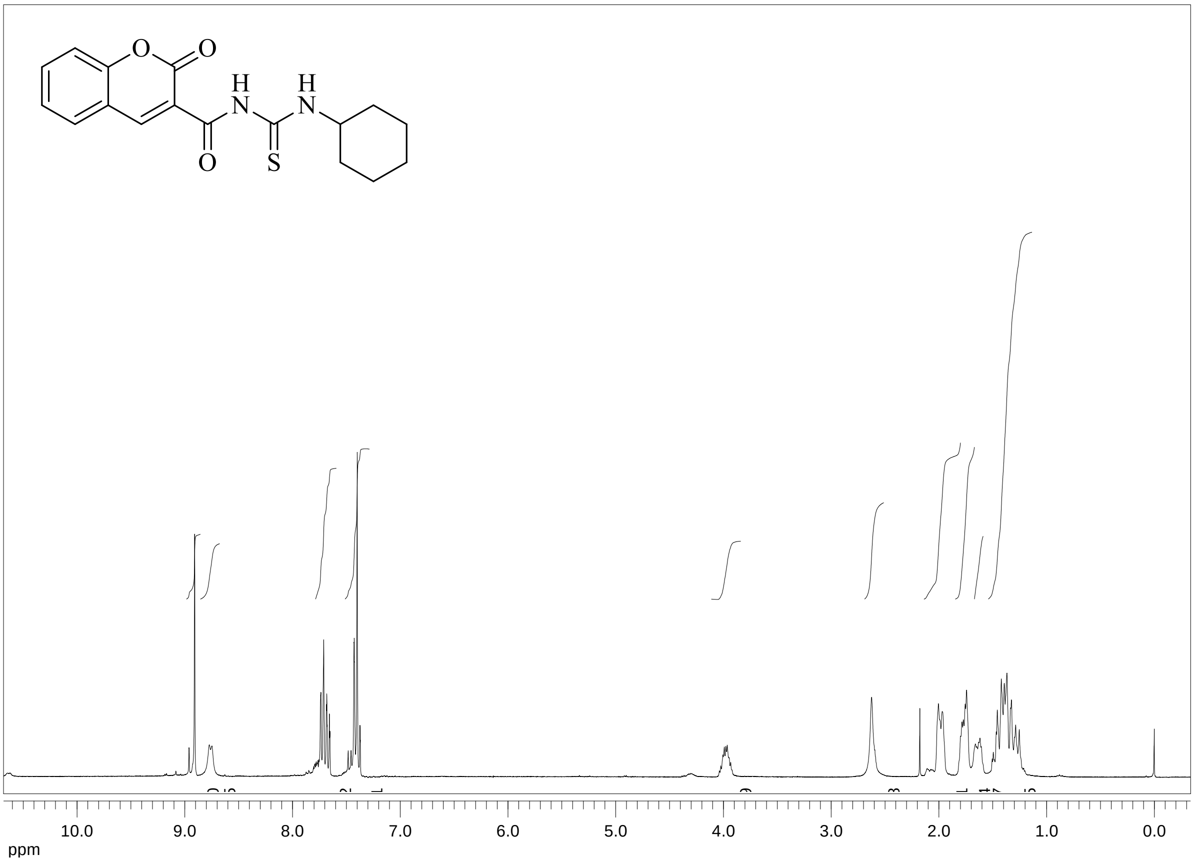

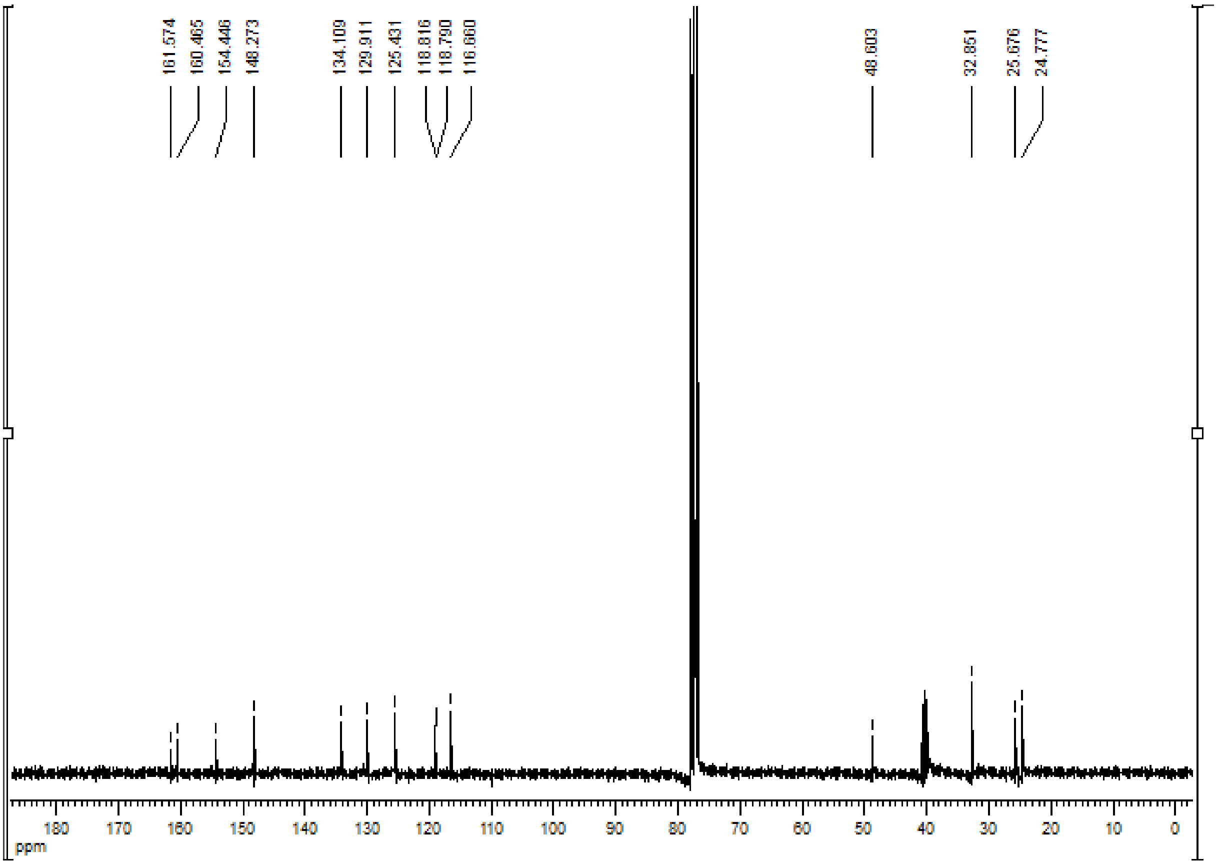

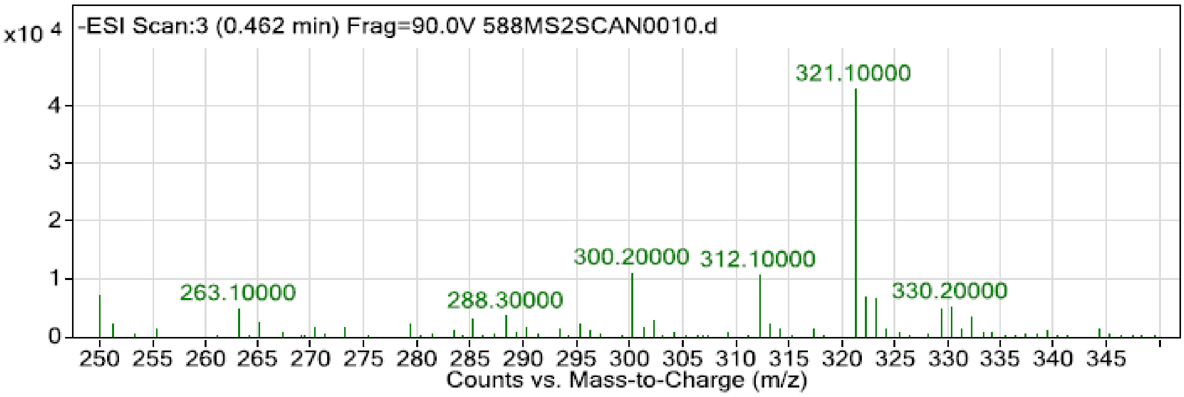

S8: [1] H NMR ($CDCl_3$), ^{13}C NMR ($CDCl_3$) and MS spectra of **e6**

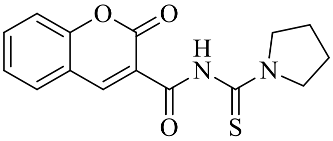

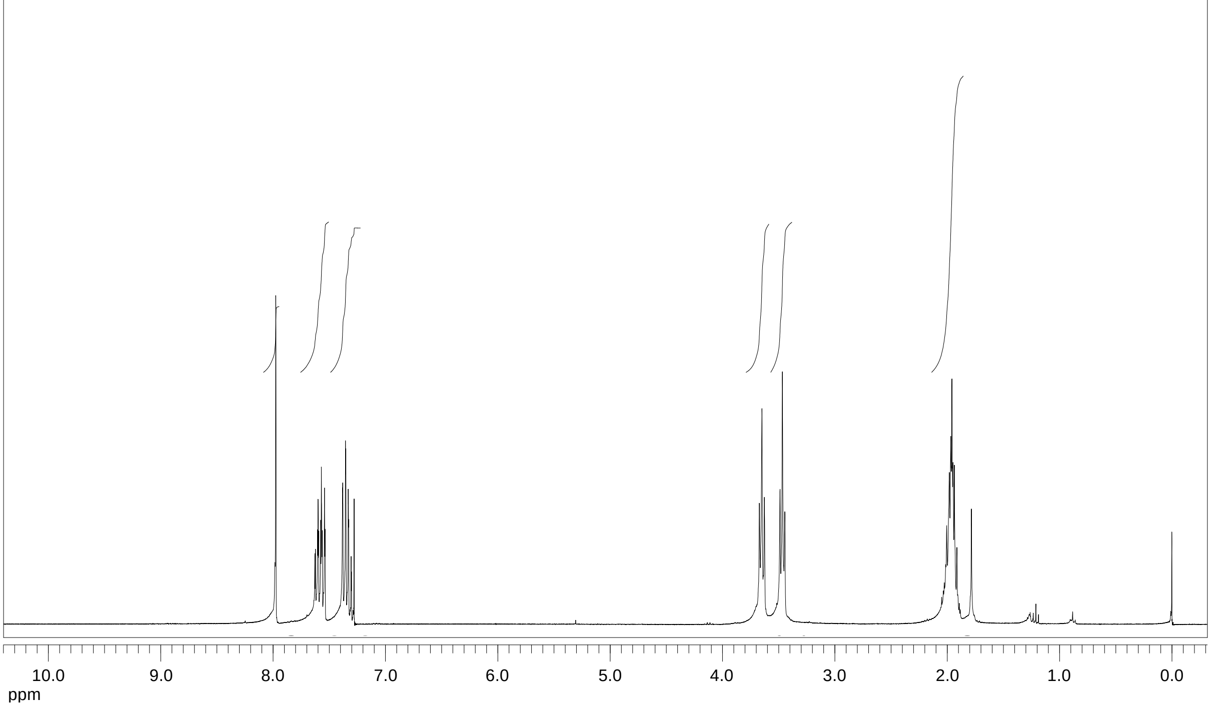

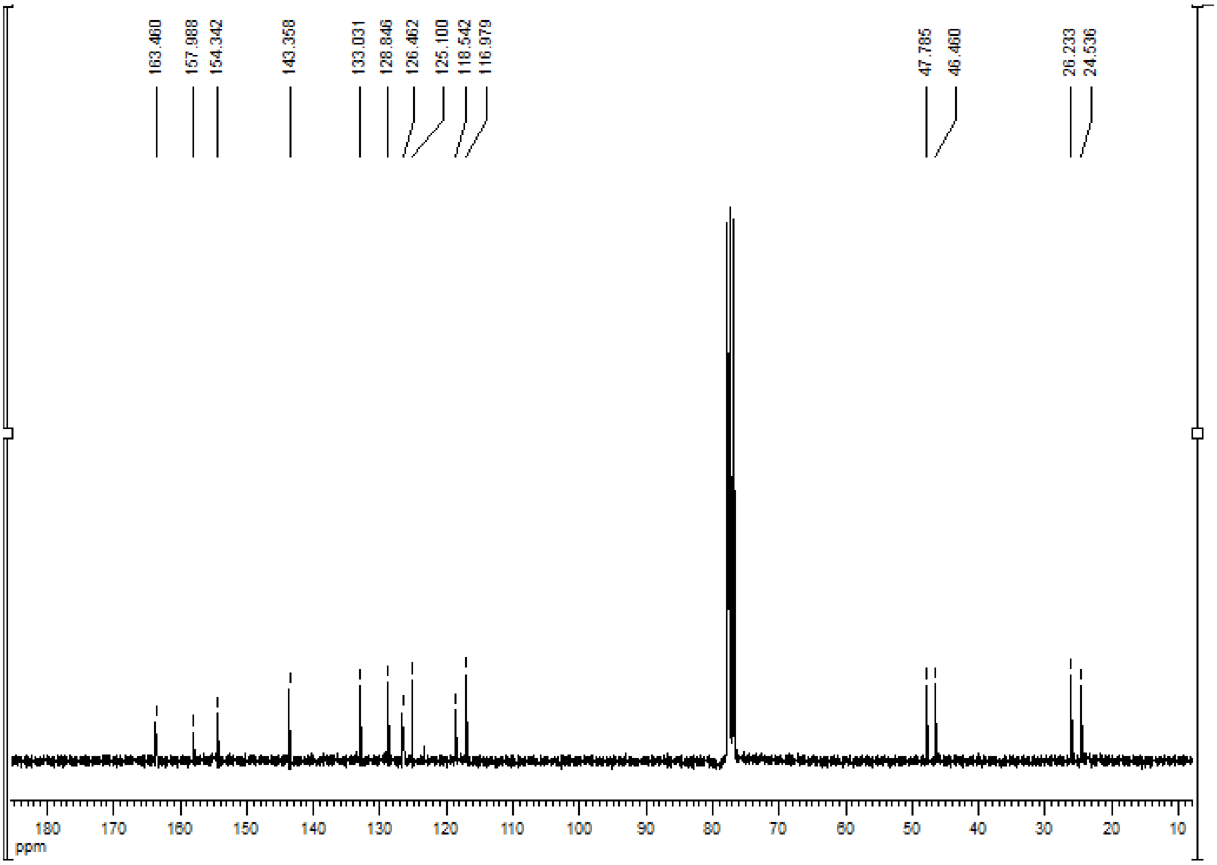

163.460
157.988
154.342
143.358
133.031
128.846
126.462
125.100
118.542
116.979
47.785
46.460
26.233
24.536
180
170
160
150
140
130
120
110
100
90
80
70
60
50
40
30
20
10
ppm

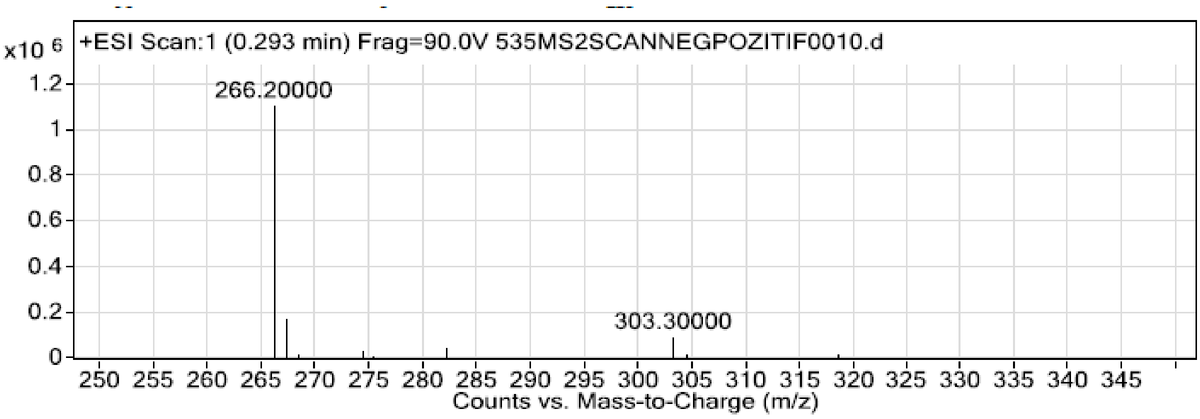

+ESI Scan:1 (0.293 min) Frag=90.0V 535MS2SCANNEGPOZITIF0010.d
x10 6
1.2
1
0.8
0.6
0.4
0.2
0
266.20000
303.30000
250 255 260 265 270 275 280 285 290 295 300 305 310 315 320 325 330 335 340 345
Counts vs. Mass-to-Charge (m/z)

S9: [1] H NMR ($CDCl_3$), ^{13}C NMR ($CDCl_3$) and MS spectra of **e7**

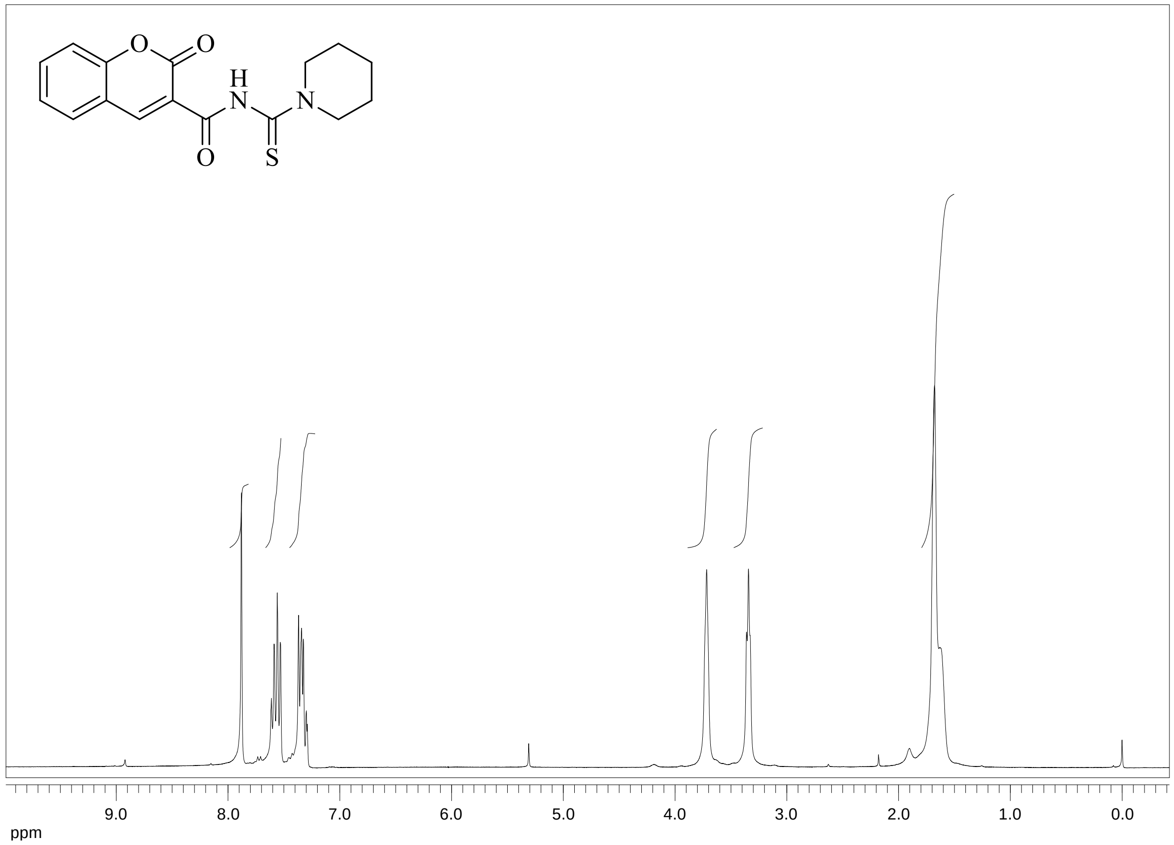

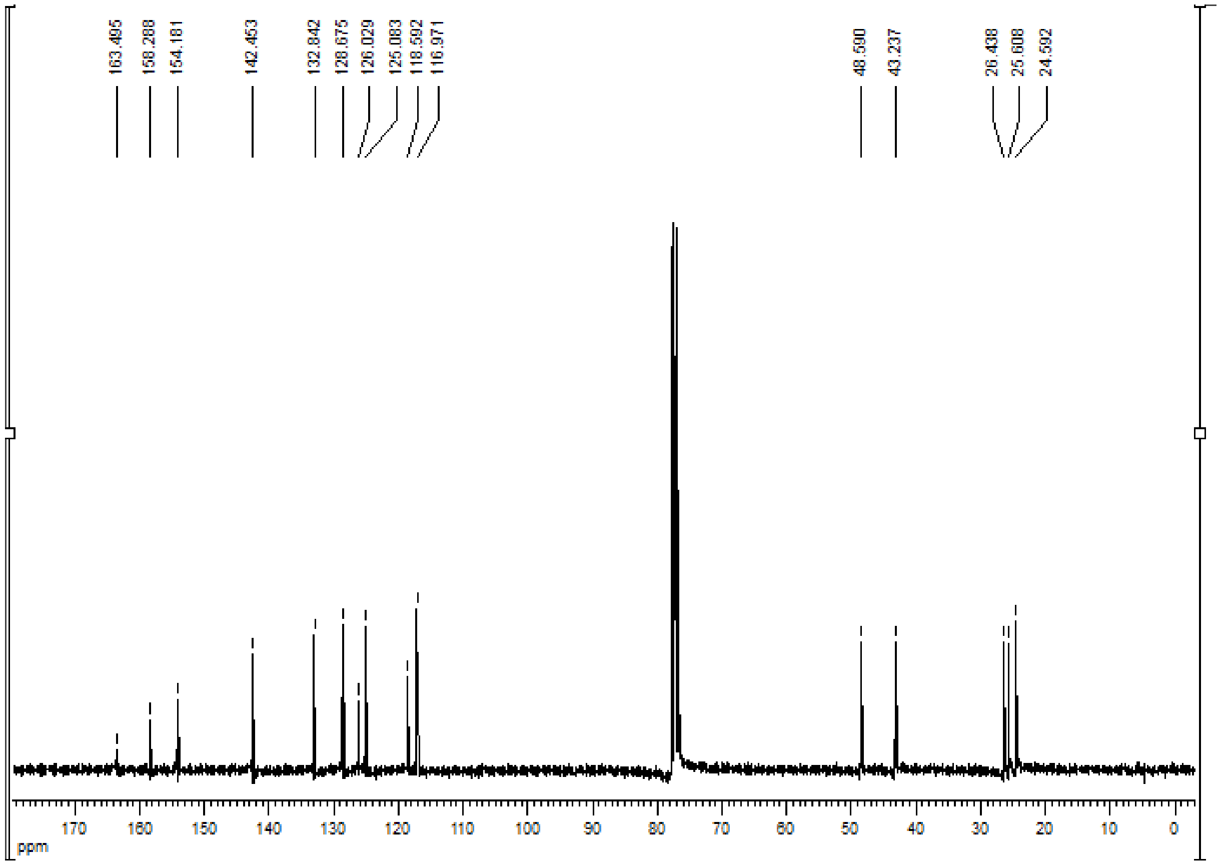

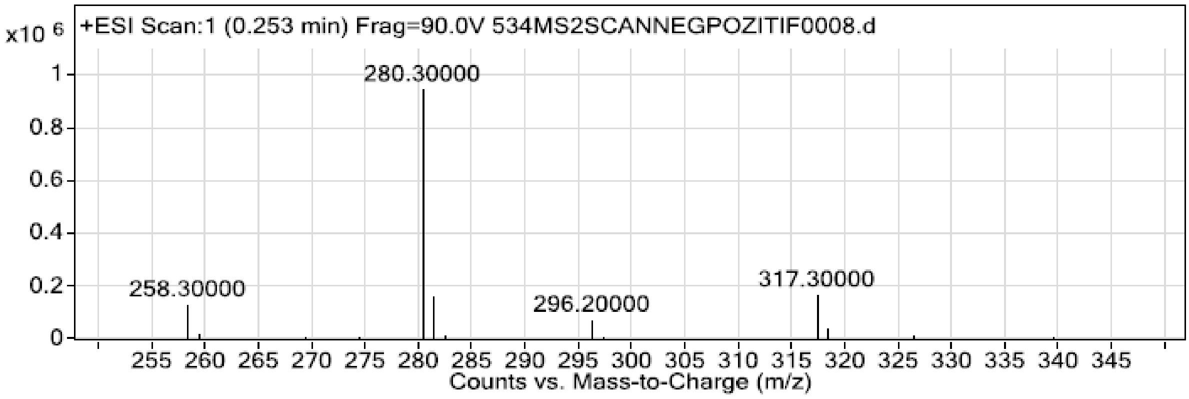

S10: [1] H NMR ($CDCl_3$), ^{13}C NMR ($CDCl_3$) and MS spectra of **e8**

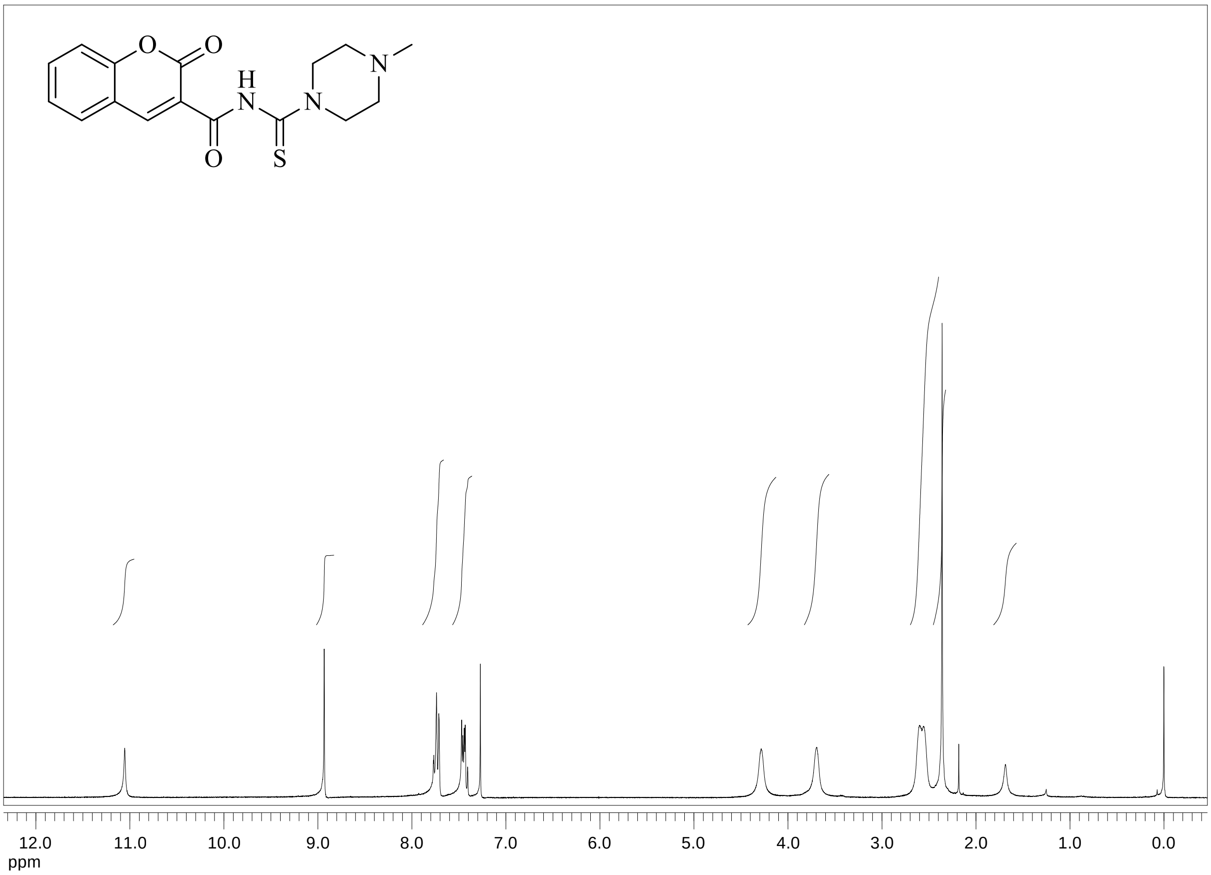

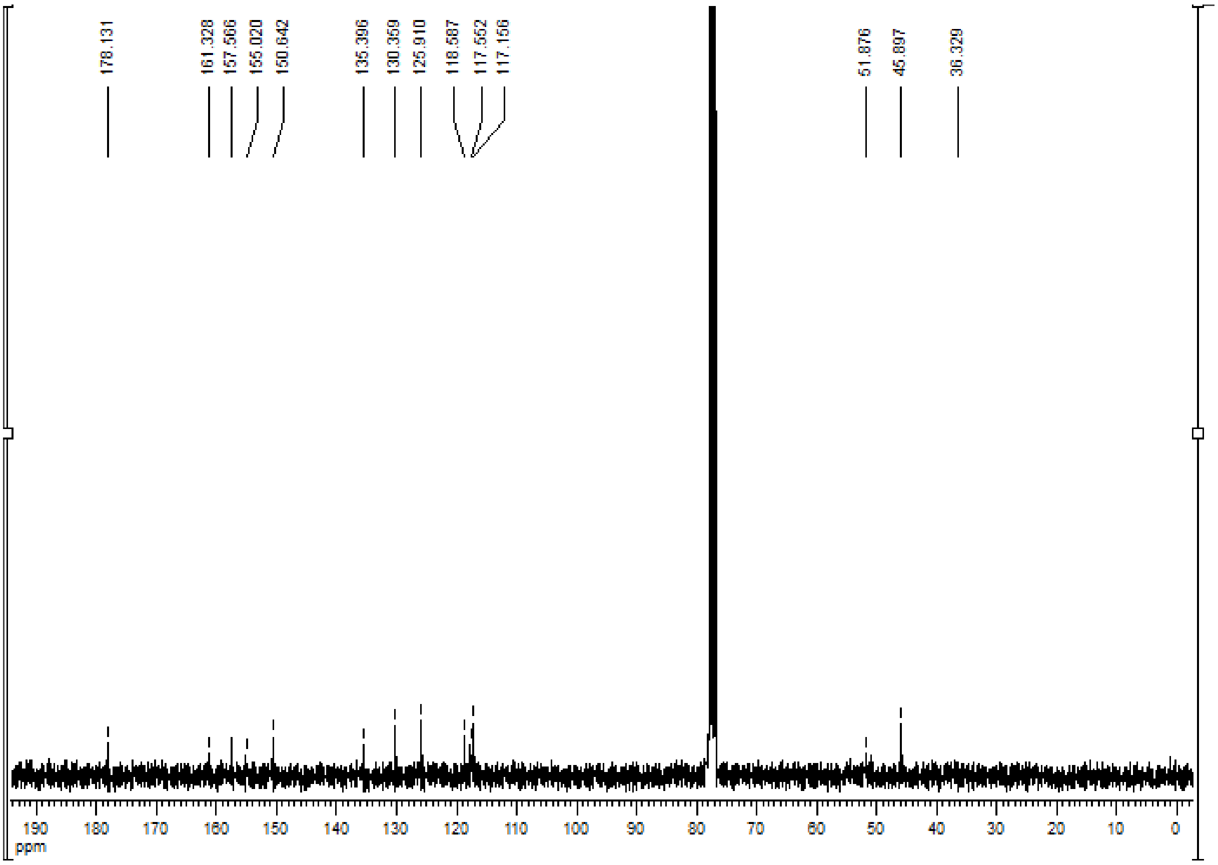
178.131
161.328
157.566
155.020
150.642
135.396
130.359
125.910
118.587
117.552
117.156
51.876
45.897
36.329
190 180 170 160 150 140 130 120 110 100 90 80 70 60 50 40 30 20 10 0
ppm

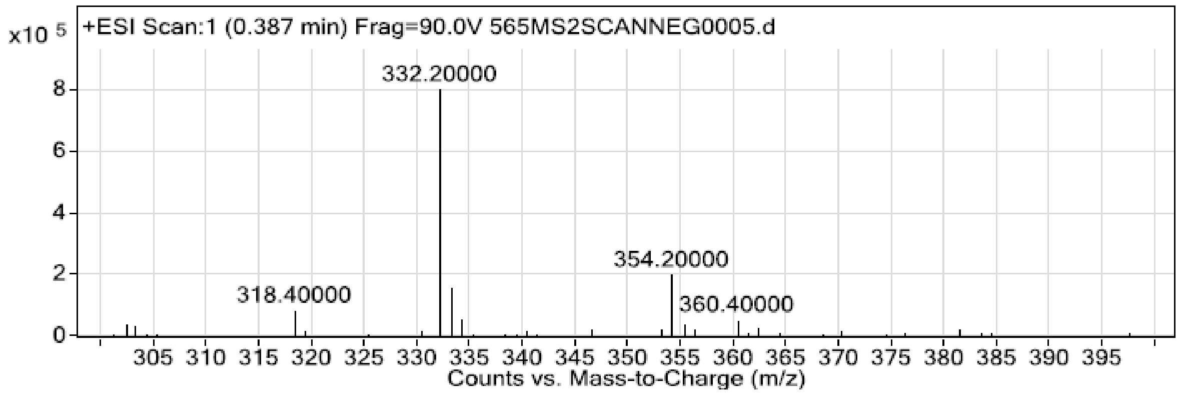
x10 5
+ESI Scan:1 (0.387 min) Frag=90.0V 565MS2SCANNEG0005.d
332.20000
354.20000
318.40000
360.40000
Counts vs. Mass-to-Charge (m/z)

S11: ^{1}H NMR ($CDCl_3$), ^{13}C NMR ($CDCl_3$) and MS spectra of **e9**

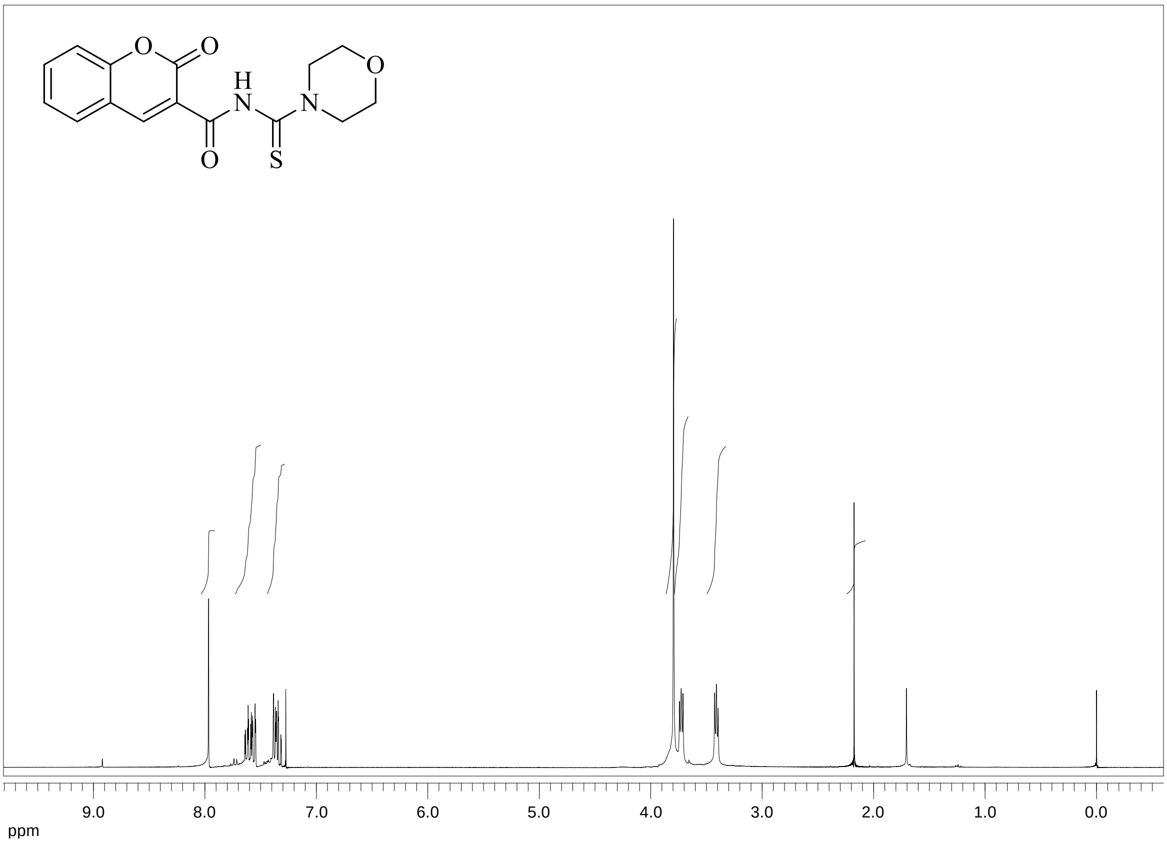

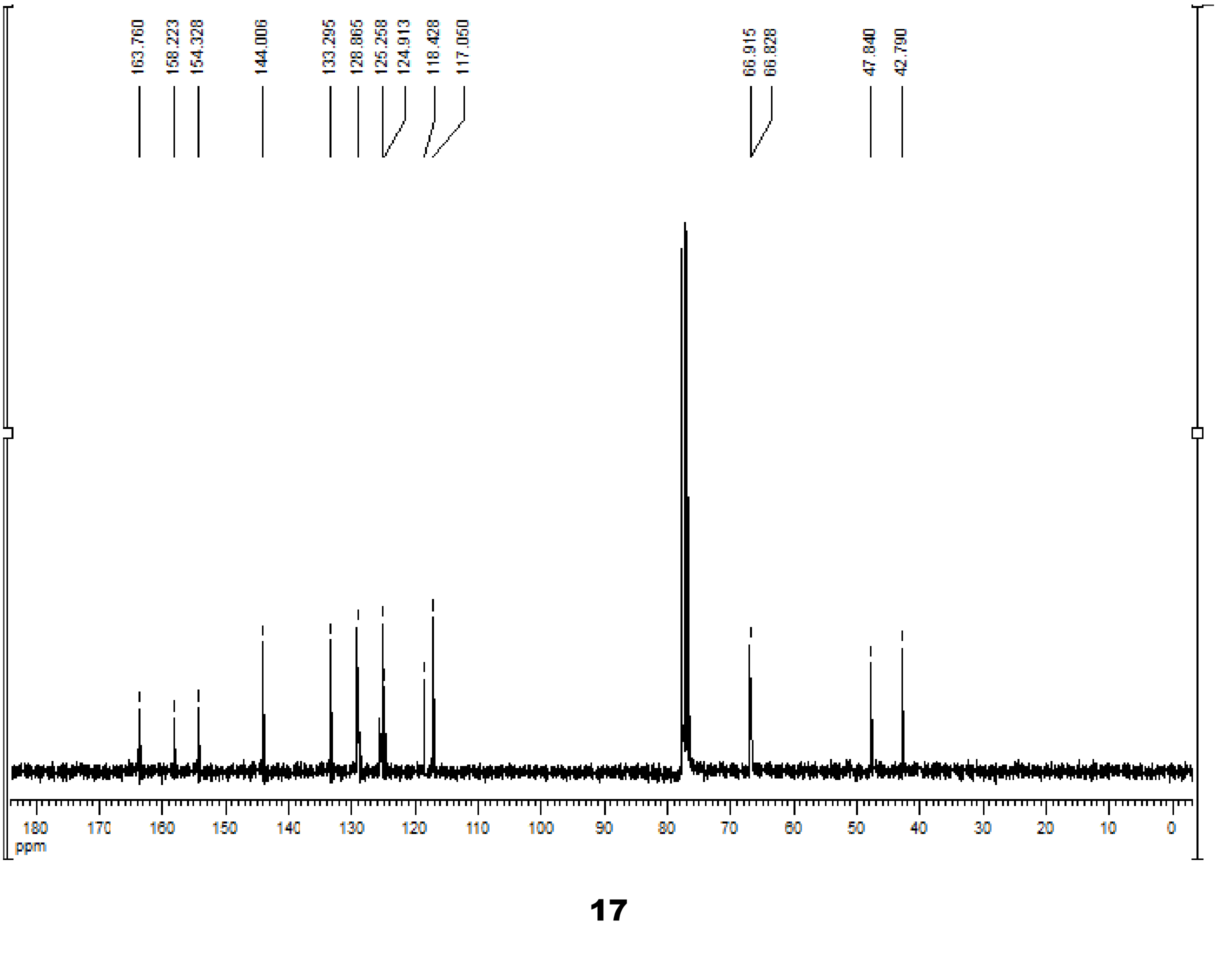

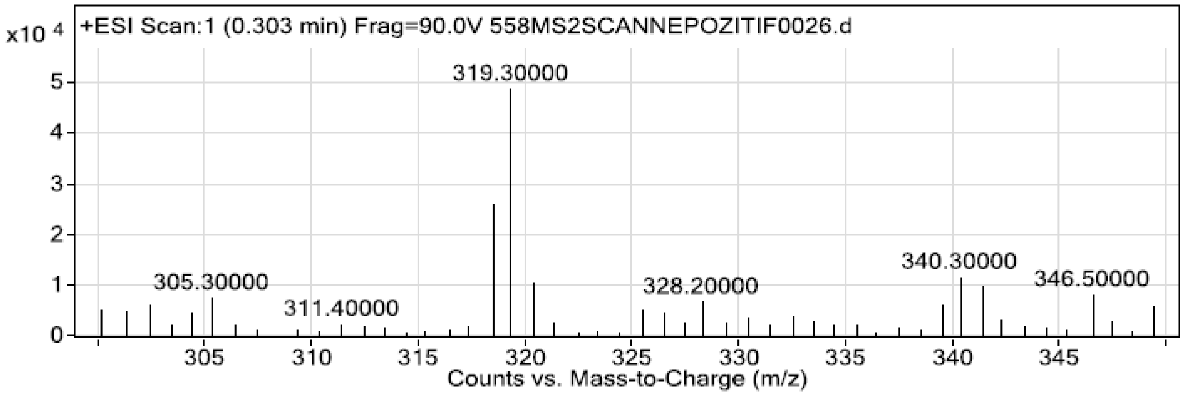

S12: ^{1} H NMR ($CDCl_3$), ^{13}C NMR ($CDCl_3$) and MS spectra of **e10**

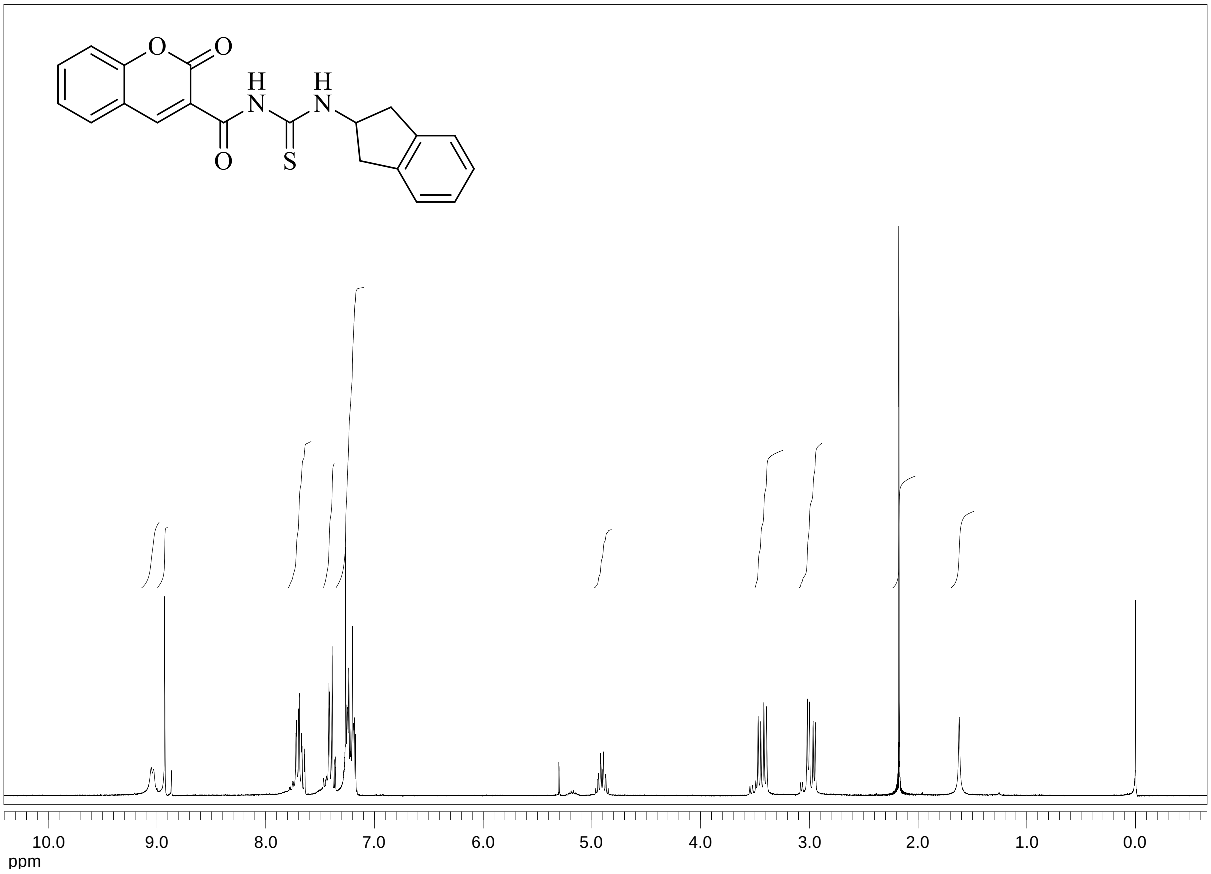

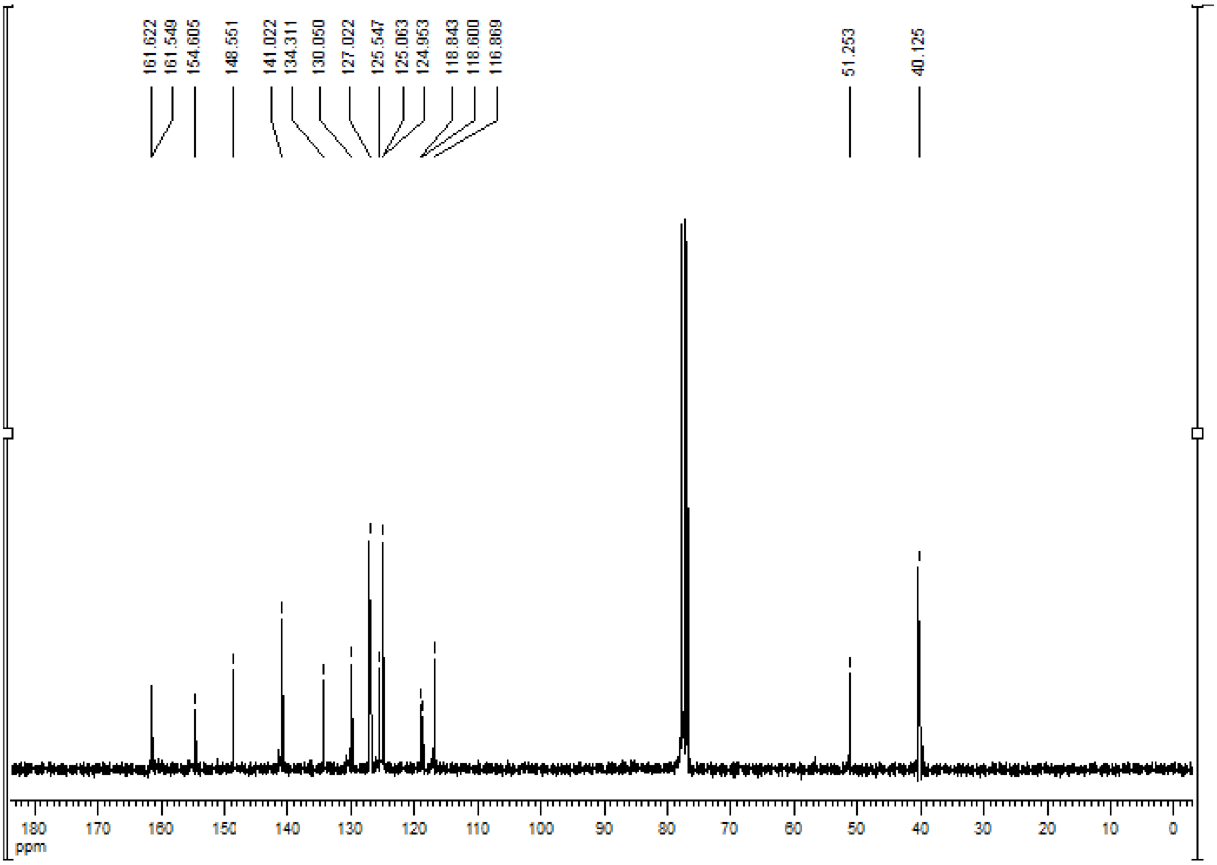
161.622
161.549
154.605
148.551
141.022
134.311
130.050
127.022
125.547
125.063
124.953
118.843
118.600
116.869
51.253
40.125
180
170
160
150
140
130
120
110
100
90
80
70
60
50
40
30
20
10
0
ppm

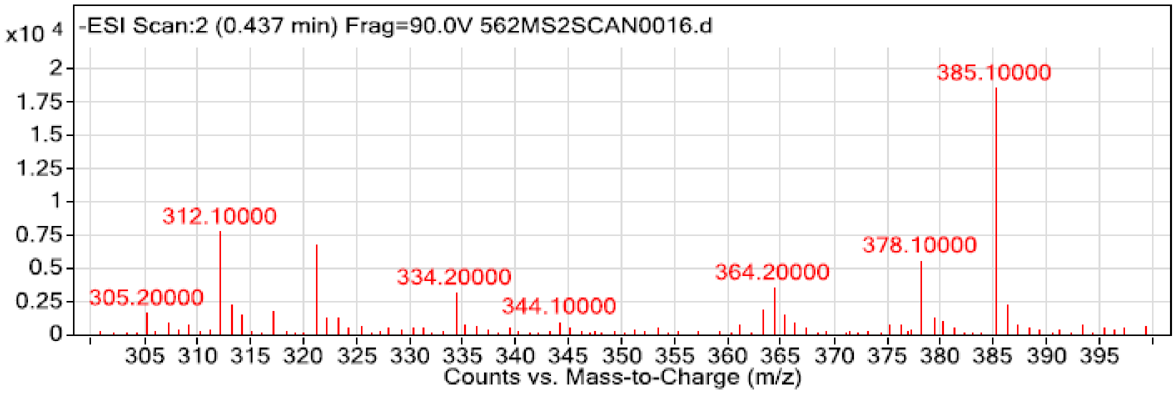
-ESI Scan:2 (0.437 min) Frag=90.0V 562MS2SCAN0016.d
x10 4
2
1.75
1.5
1.25
1
0.75
0.5
0.25
0
385.10000
312.10000
378.10000
334.20000
364.20000
305.20000
344.10000
305 310 315 320 325 330 335 340 345 350 355 360 365 370 375 380 385 390 395
Counts vs. Mass-to-Charge (m/z)

S13: [1] H NMR ($CDCl_3$), ^{13}C NMR ($CDCl_3$) and MS spectra of **e11**

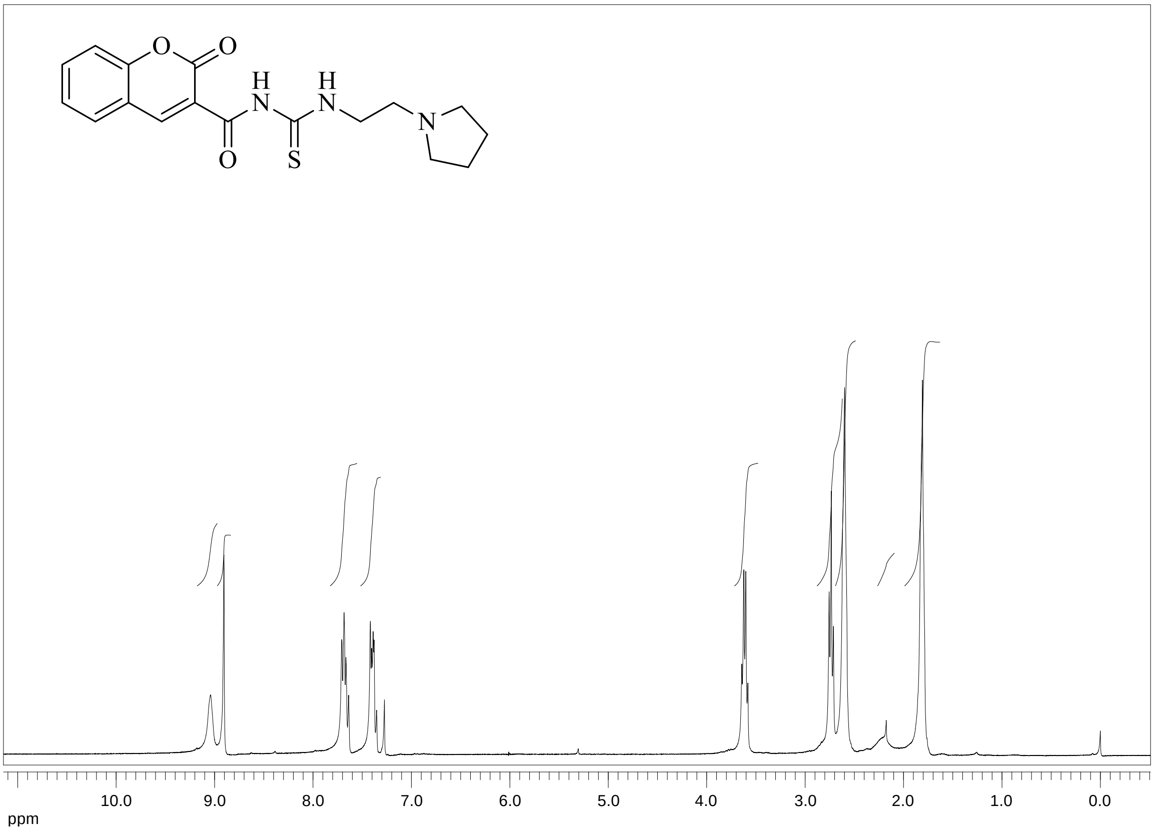

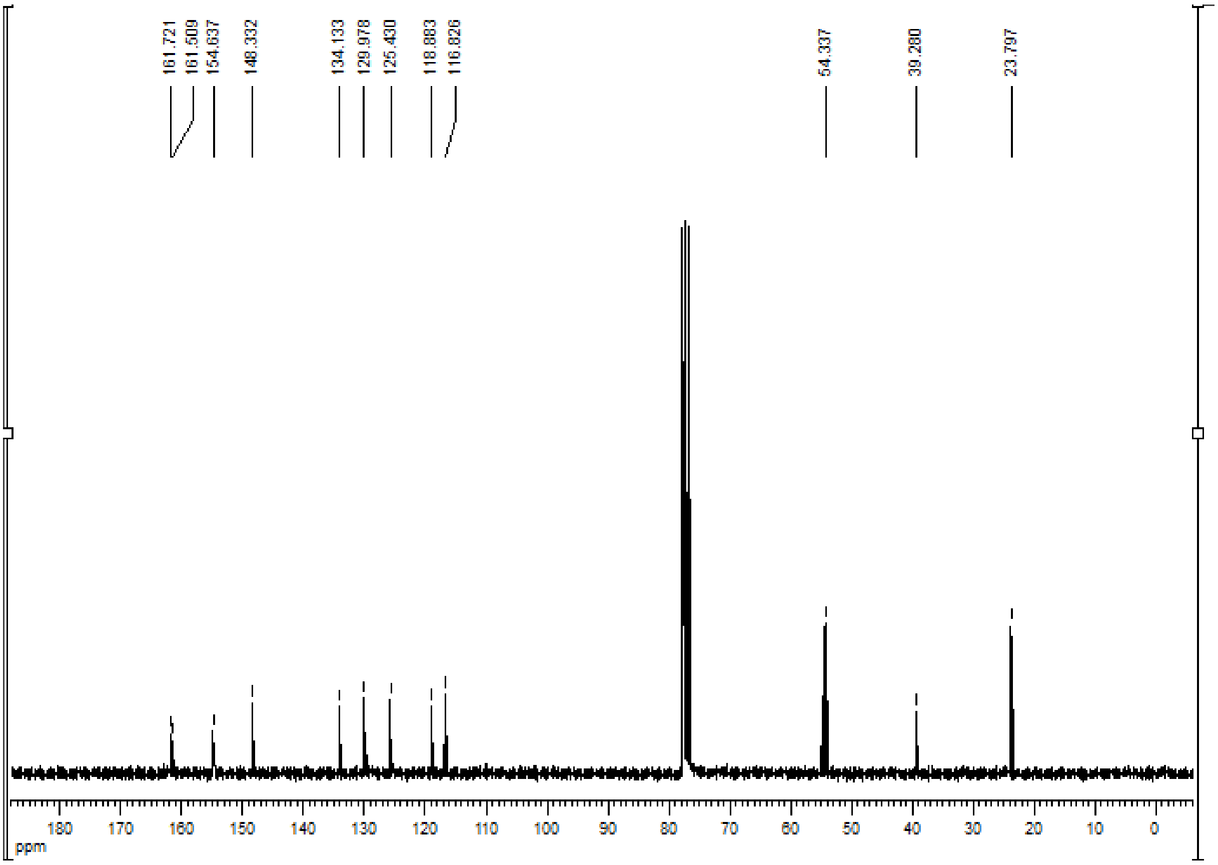

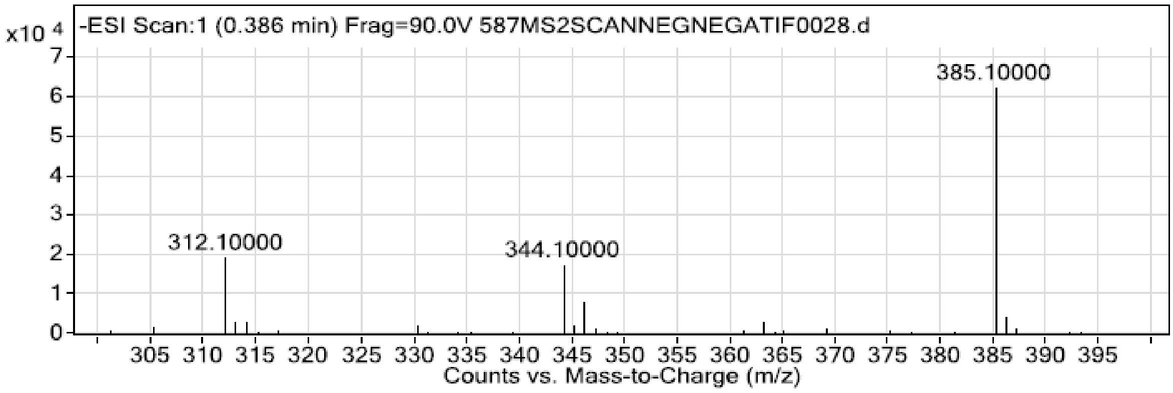

S14: ^{1} H NMR ($CDCl_3$), ^{13}C NMR ($CDCl_3$) and MS spectra of **e12**

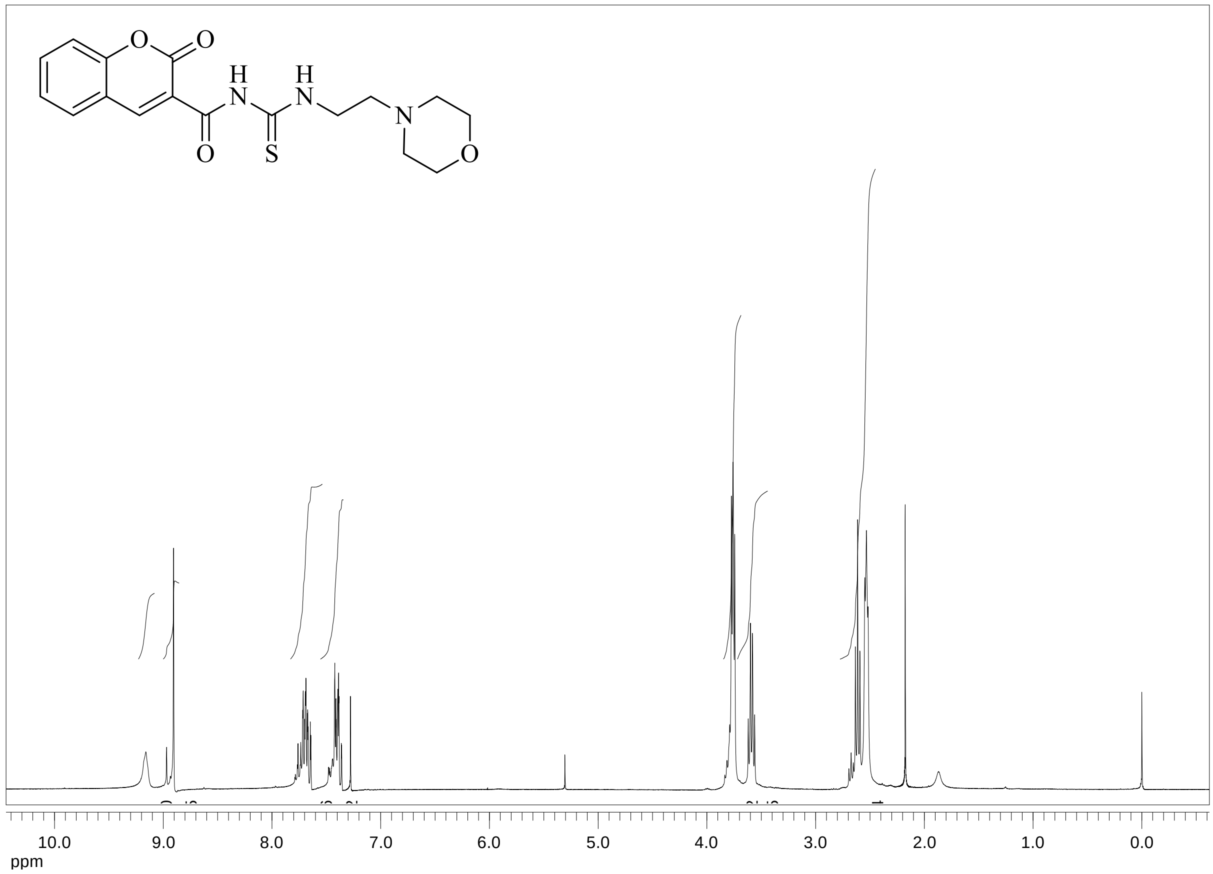

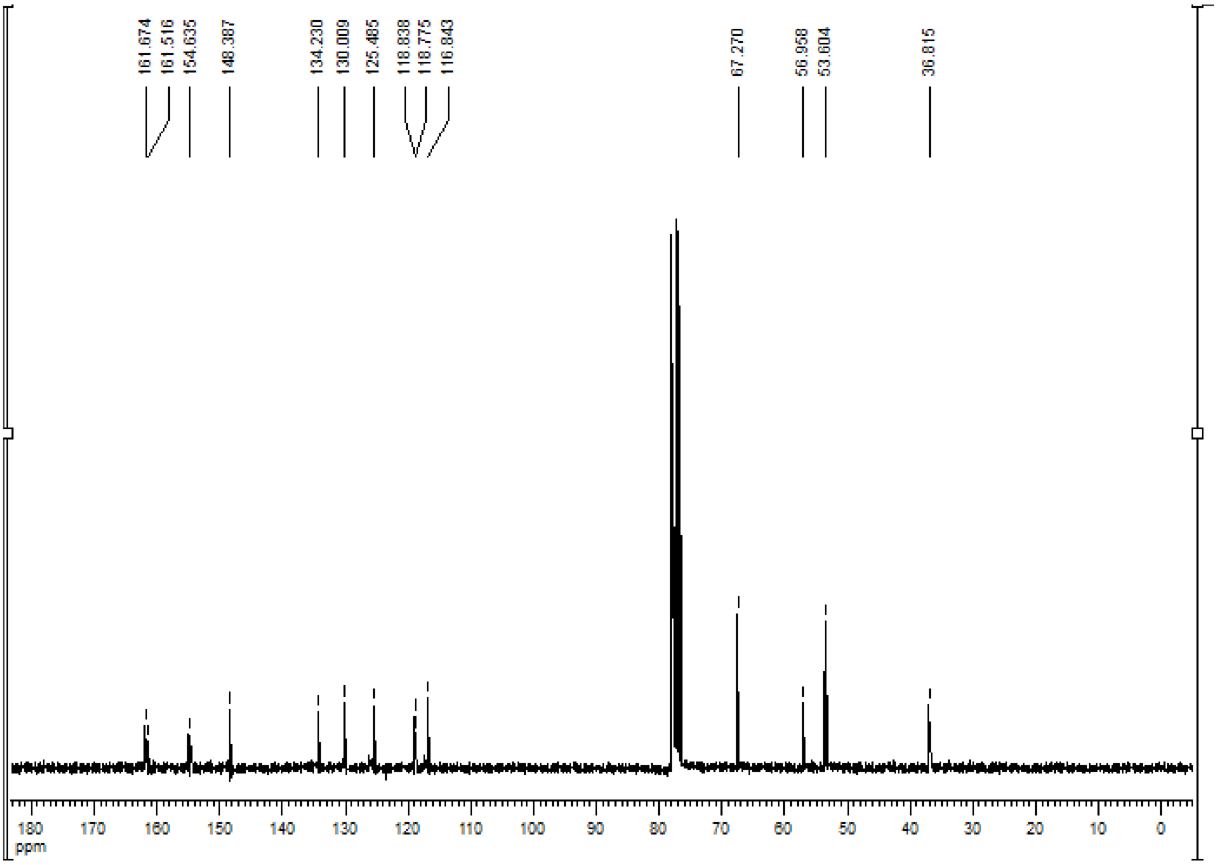

161.674
161.516
154.635
148.387
134.230
130.009
125.485
118.838
118.775
116.843
67.270
56.958
53.604
36.815
180 170 160 150 140 130 120 110 100 90 80 70 60 50 40 30 20 10 0
ppm

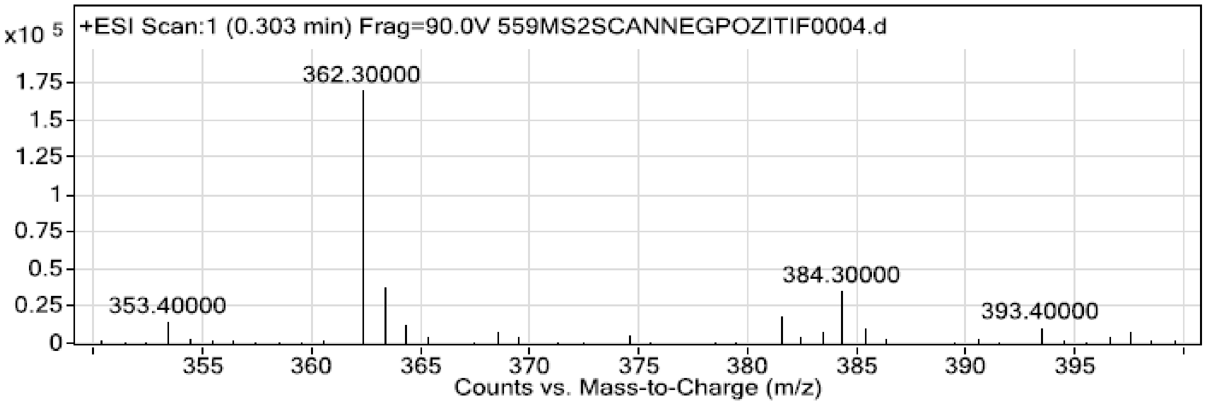

x10 5
+ESI Scan:1 (0.303 min) Frag=90.0V 559MS2SCANNEGPOZITIF0004.d
362.30000
353.40000
384.30000
393.40000
Counts vs. Mass-to-Charge (m/z)

S15: [1] H NMR ($CDCl_3$), ^{13}C NMR ($CDCl_3$) and MS spectra of **e13**

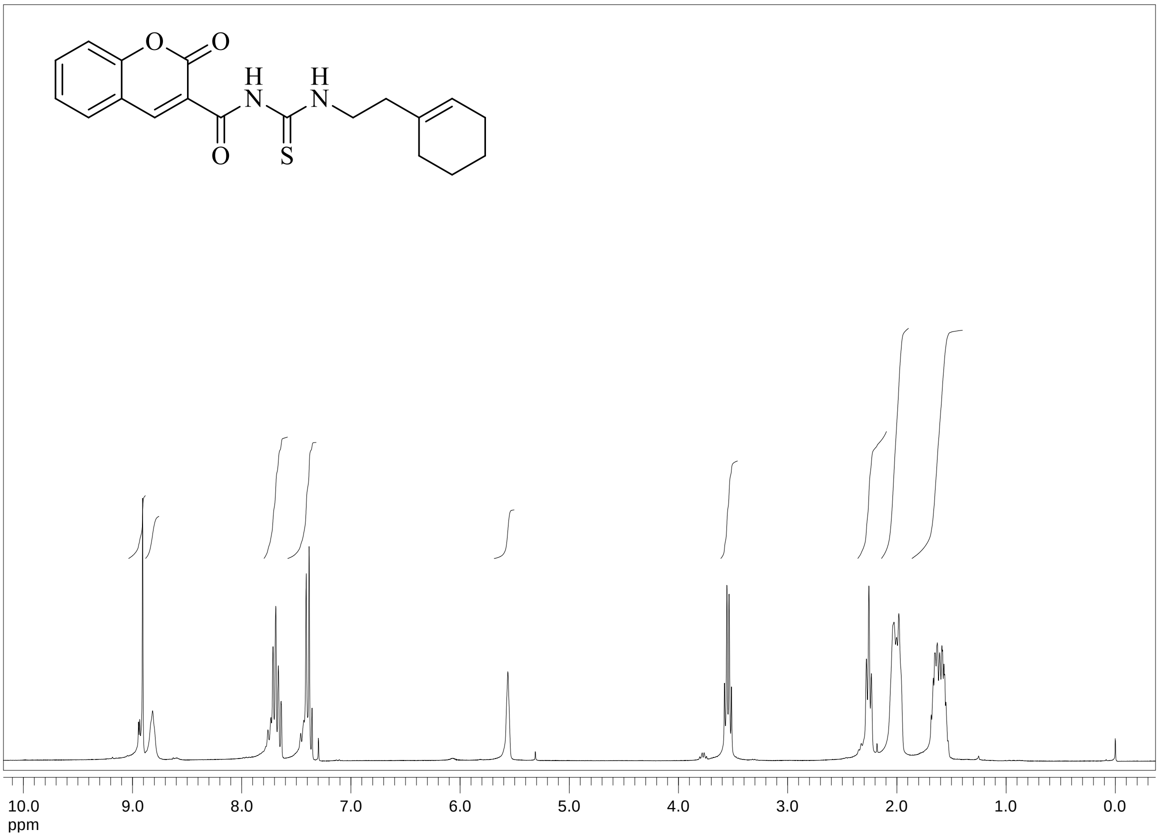

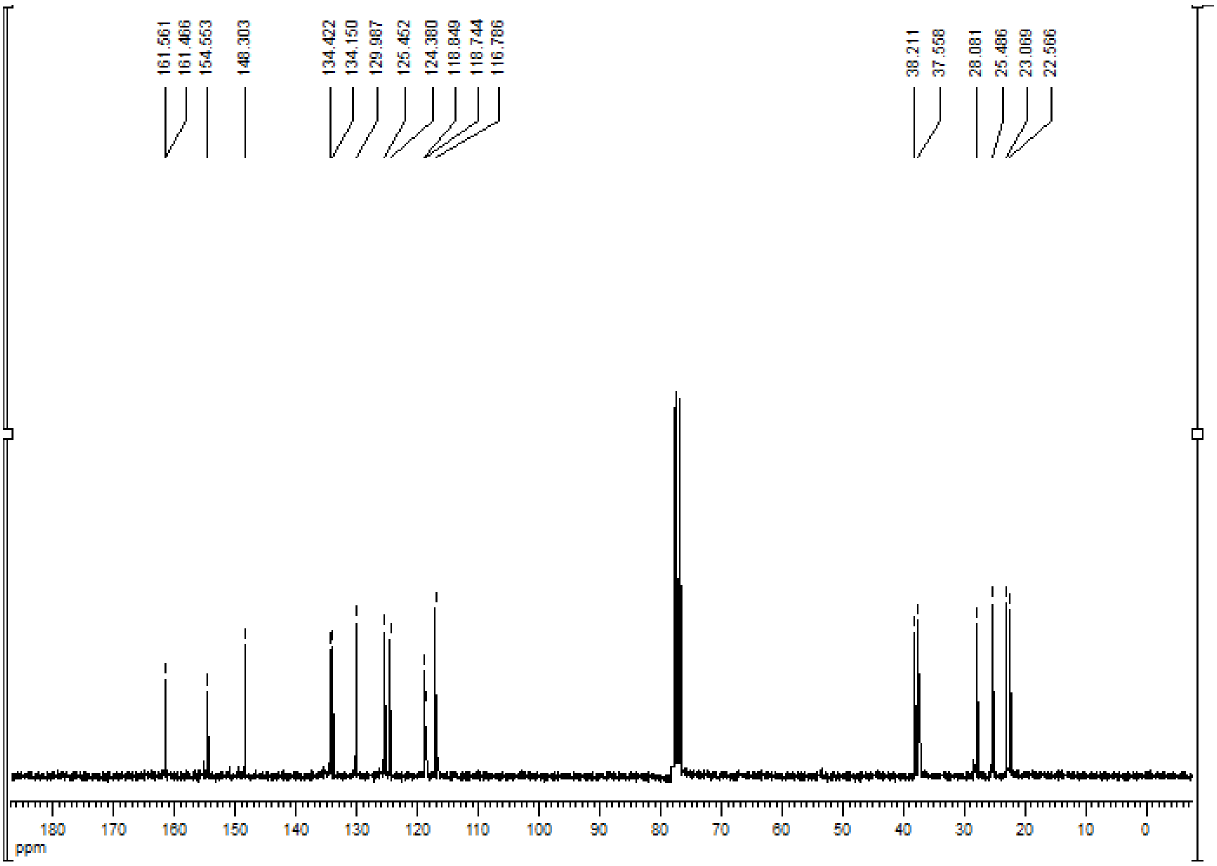

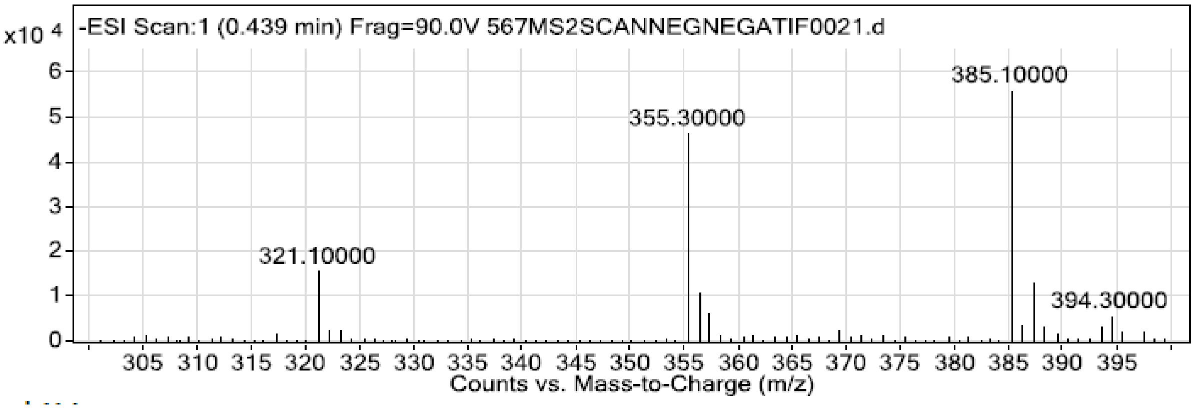

S16: [1] H NMR ($CDCl_3$), ^{13}C NMR ($CDCl_3$) and MS spectra of **e14**

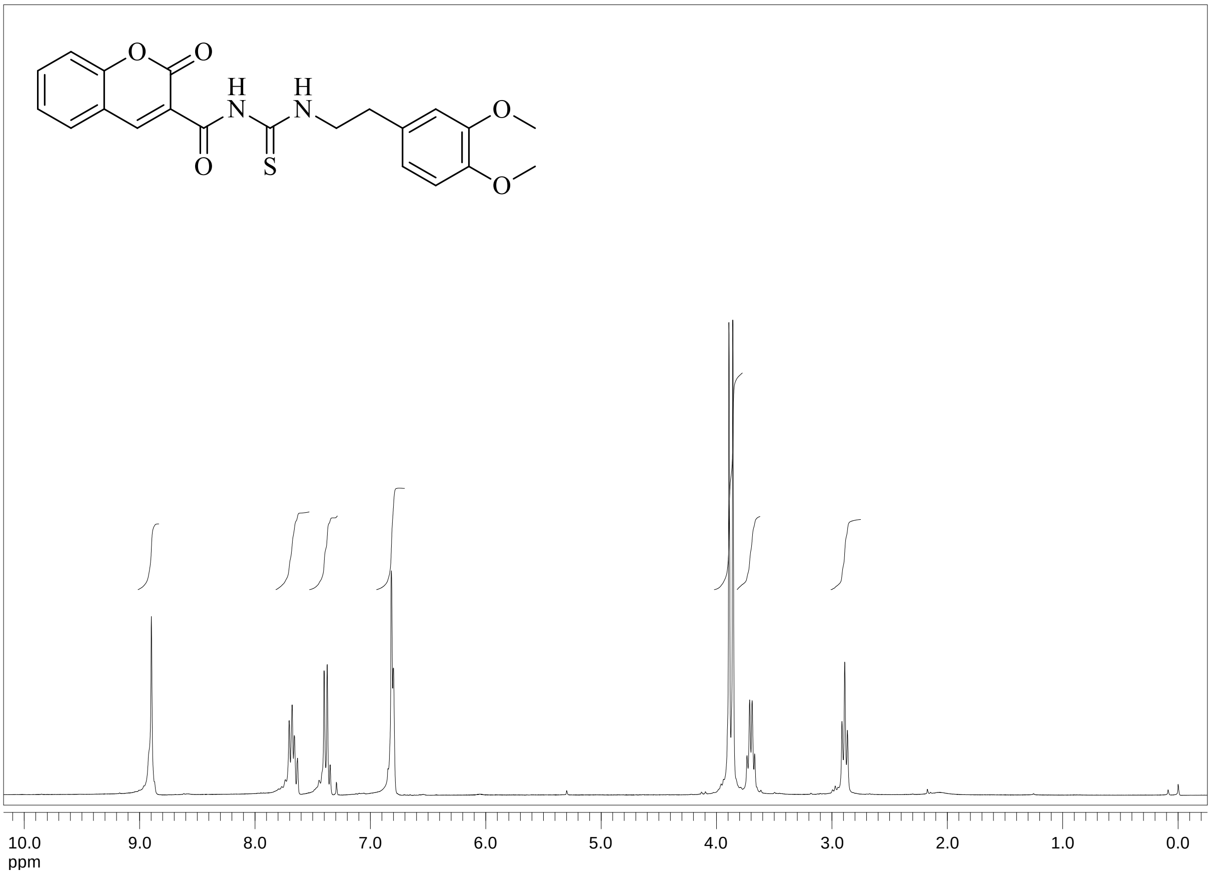

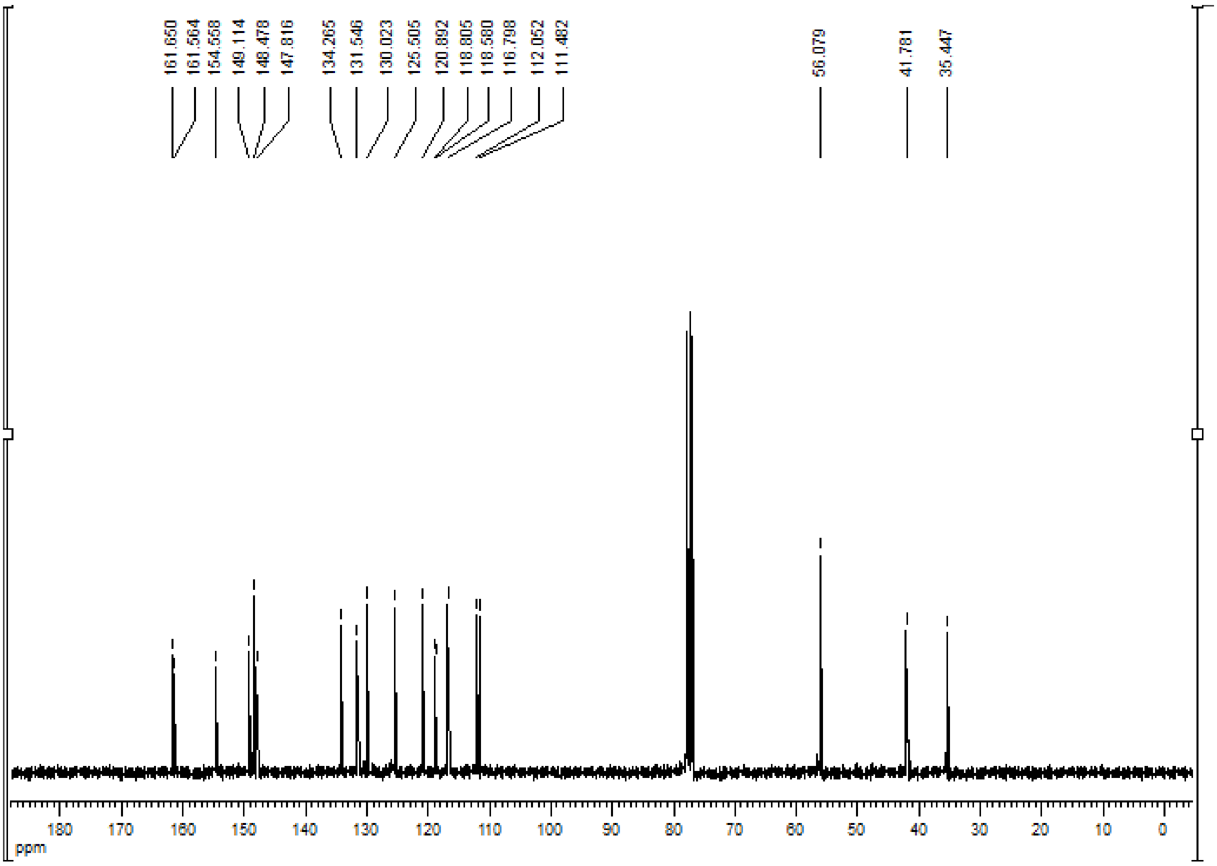
161.650
161.564
154.558
149.114
148.478
147.816
134.265
131.546
130.023
125.505
120.892
118.805
118.580
116.798
112.052
111.482
56.079
41.781
35.447
180 170 160 150 140 130 120 110 100 90 80 70 60 50 40 30 20 10 0
ppm

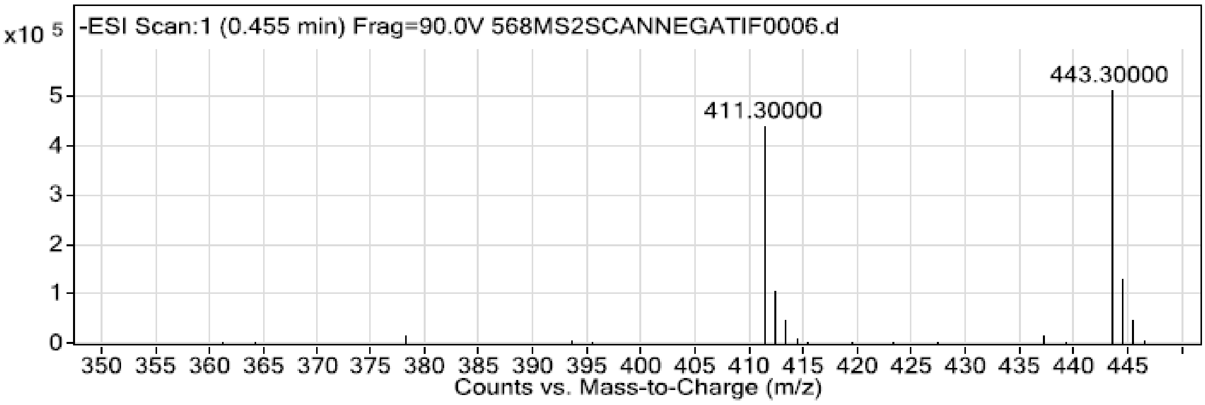
-ESI Scan:1 (0.455 min) Frag=90.0V 568MS2SCANNEGATIF0006.d
x10 5
443.30000
411.30000
350 355 360 365 370 375 380 385 390 395 400 405 410 415 420 425 430 435 440 445
Counts vs. Mass-to-Charge (m/z)

S17: [1] H NMR ($CDCl_3$), ^{13}C NMR ($CDCl_3$) and MS spectra of **e15**

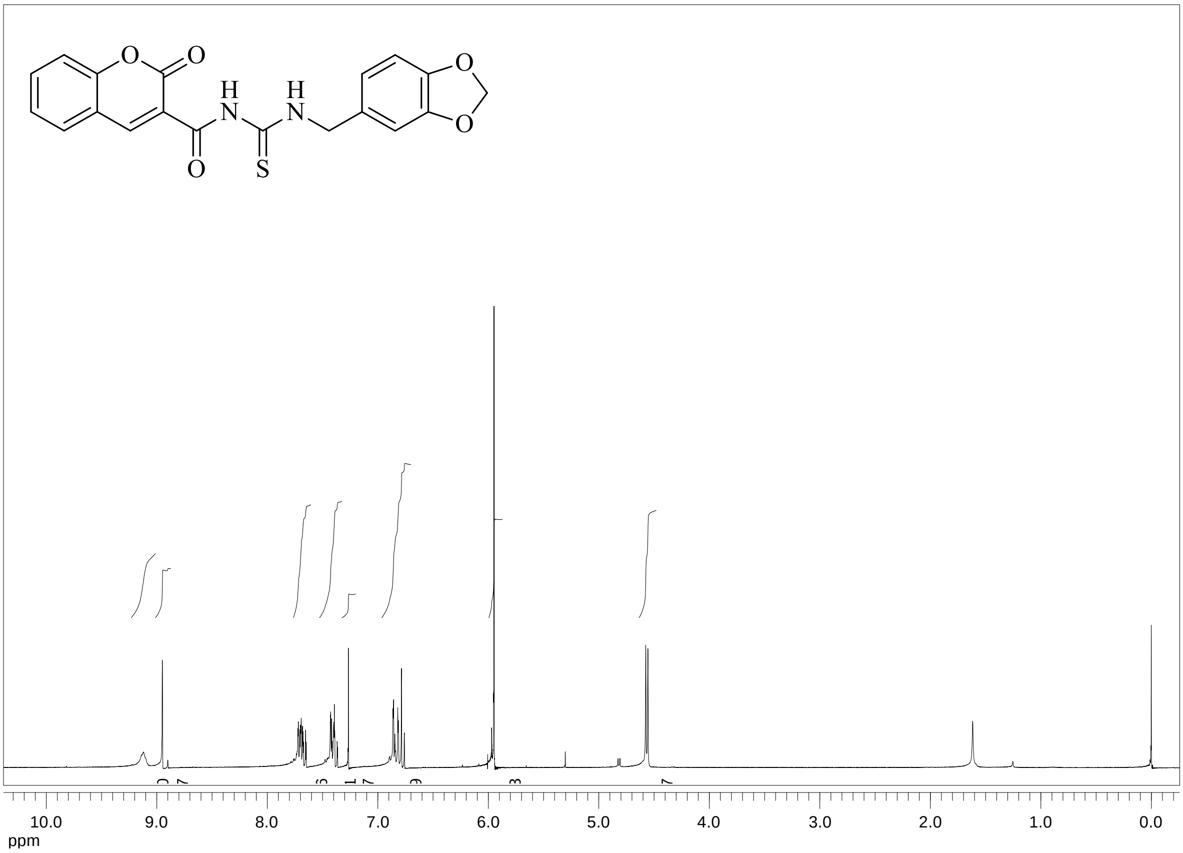

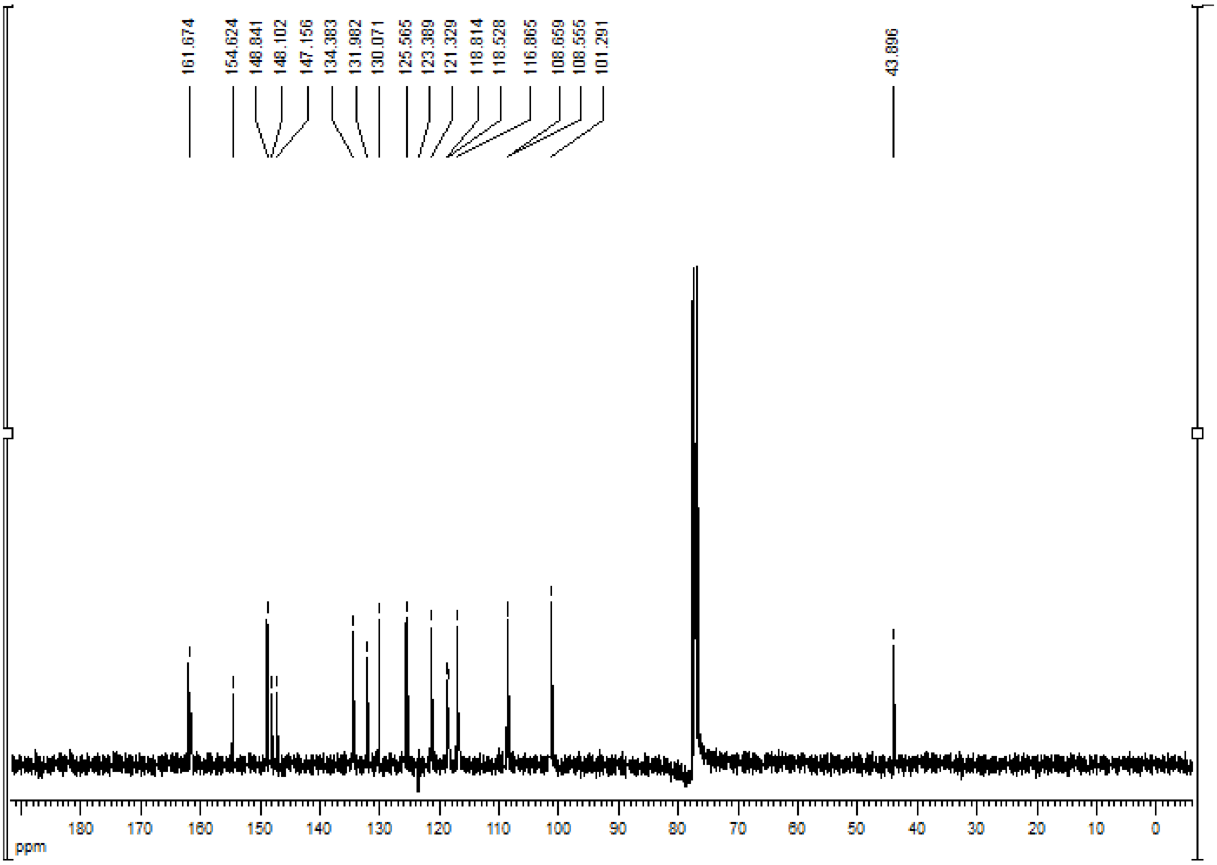

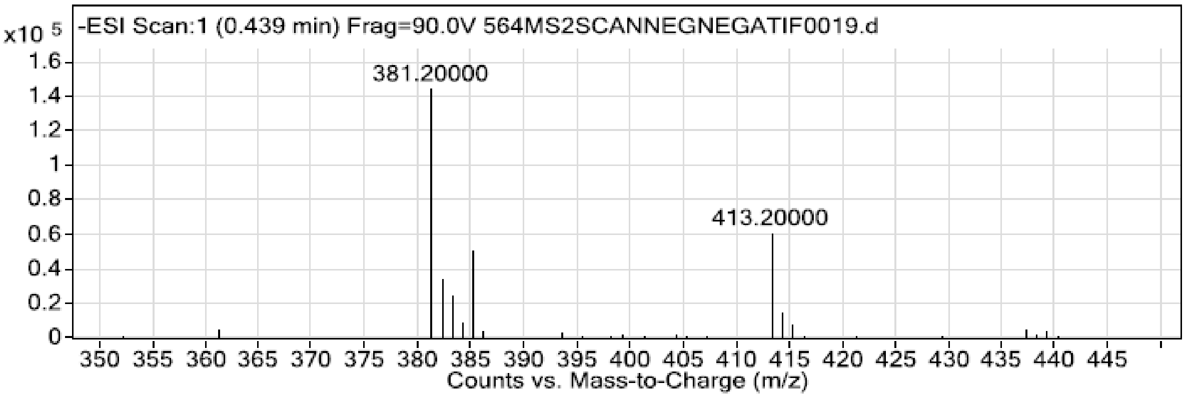

S18: [1] H NMR ($CDCl_3$), ^{13}C NMR ($CDCl_3$) and MS spectra of **e16**

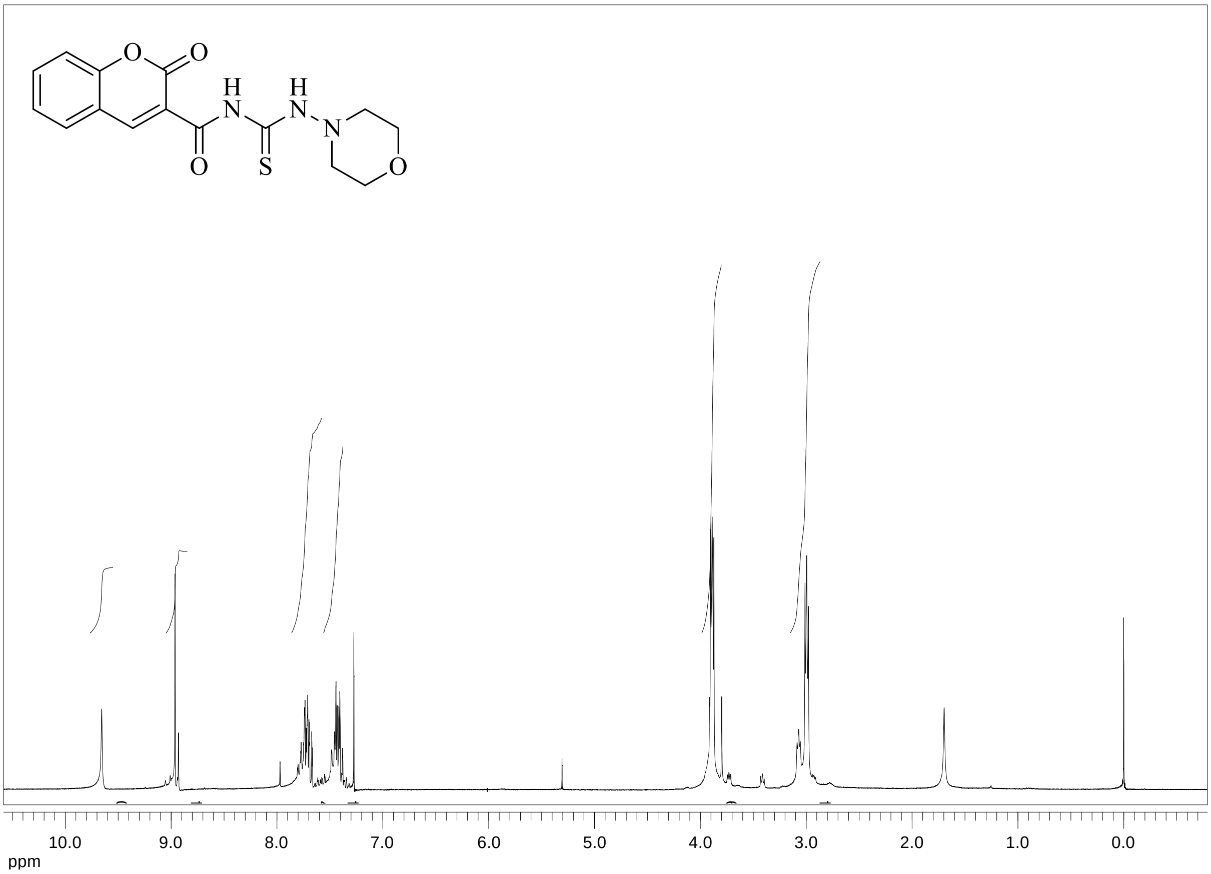

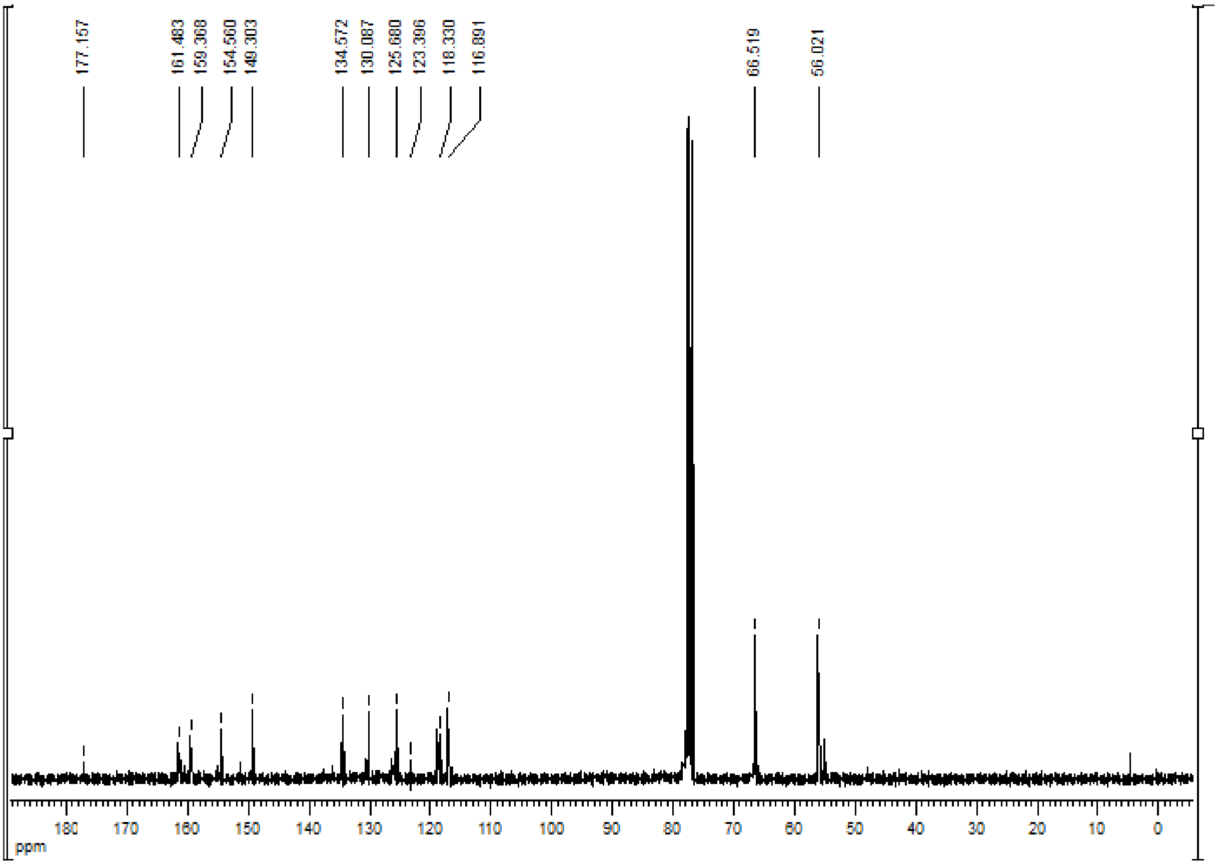
177.157
161.483
159.368
154.560
149.303
134.572
130.087
125.680
123.396
118.330
116.891
66.519
56.021
180 170 160 150 140 130 120 110 100 90 80 70 60 50 40 30 20 10 0
ppm

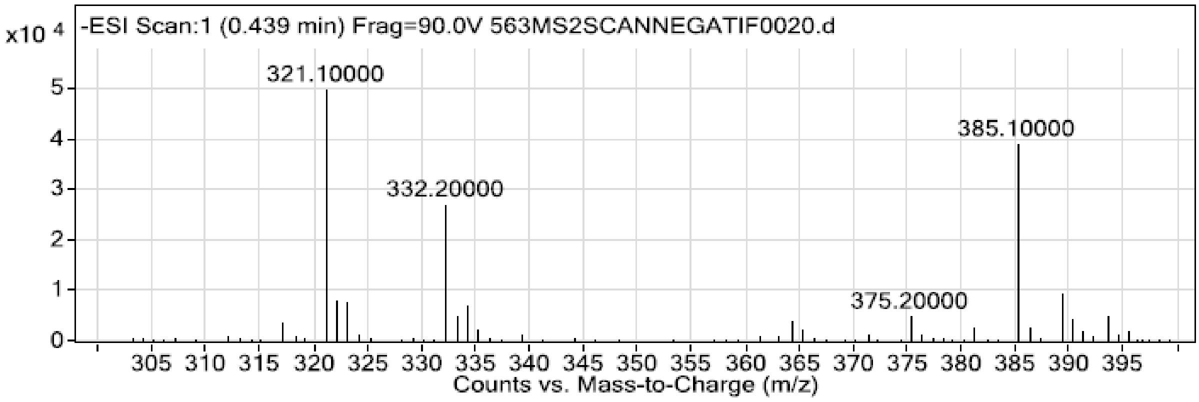
x10 4
-ESI Scan:1 (0.439 min) Frag=90.0V 563MS2SCANNEGATIF0020.d
321.10000
332.20000
385.10000
375.20000
0 1 2 3 4 5
305 310 315 320 325 330 335 340 345 350 355 360 365 370 375 380 385 390 395
Counts vs. Mass-to-Charge (m/z)

S19: ^{1}H NMR ($CDCl_3$), ^{13}C NMR ($CDCl_3$) and MS spectra of **e17**

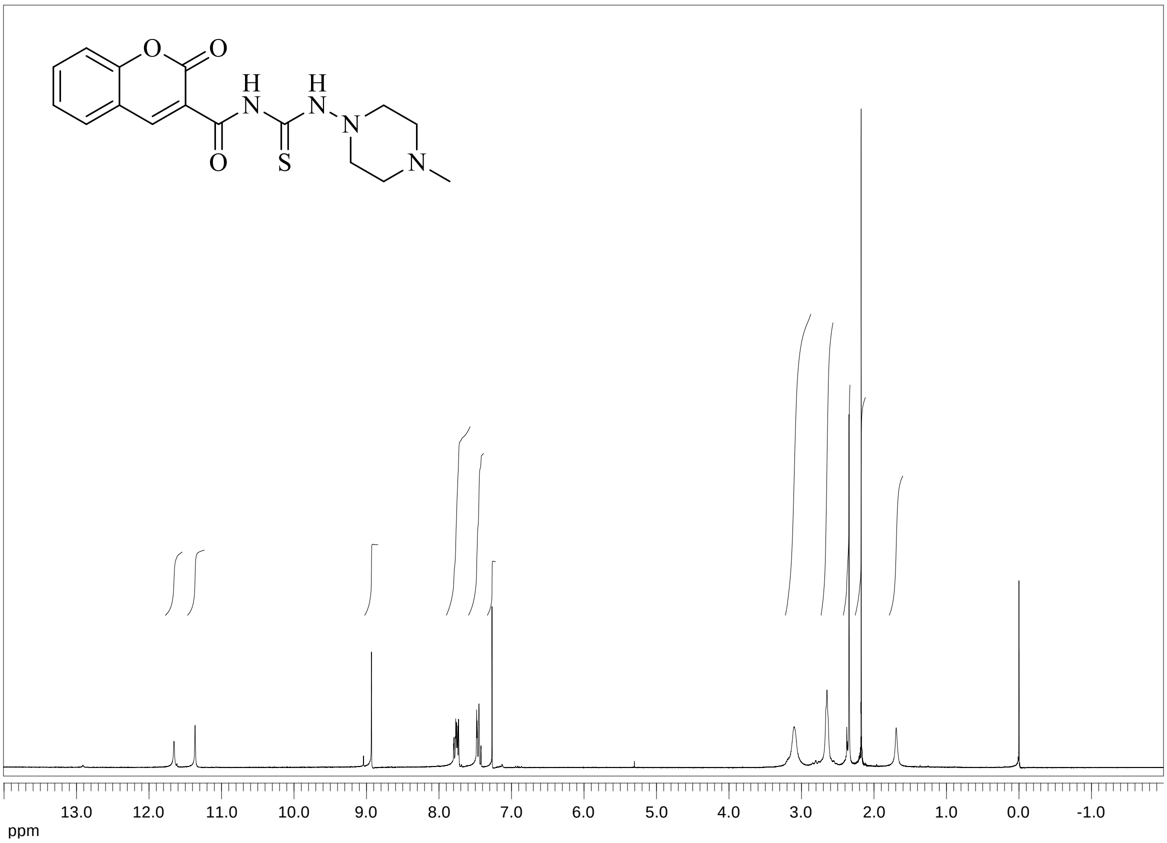

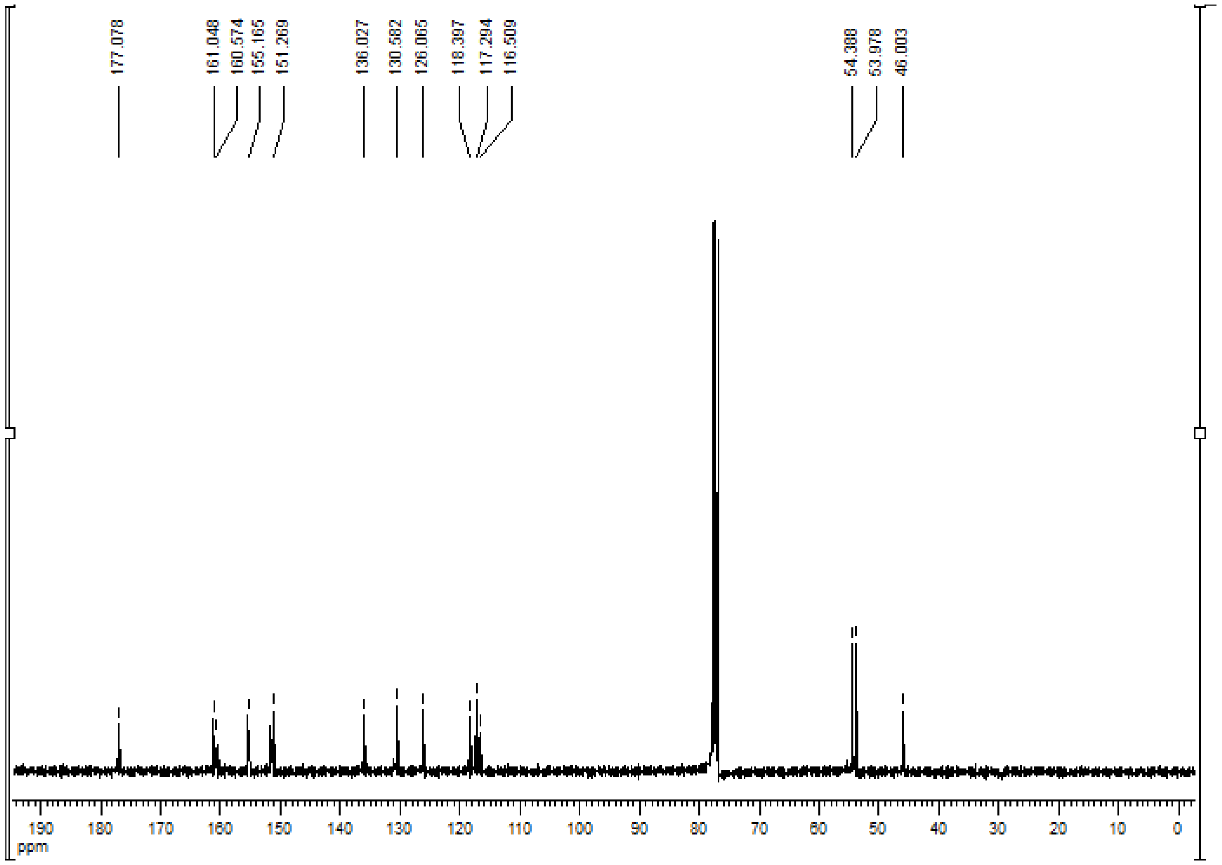

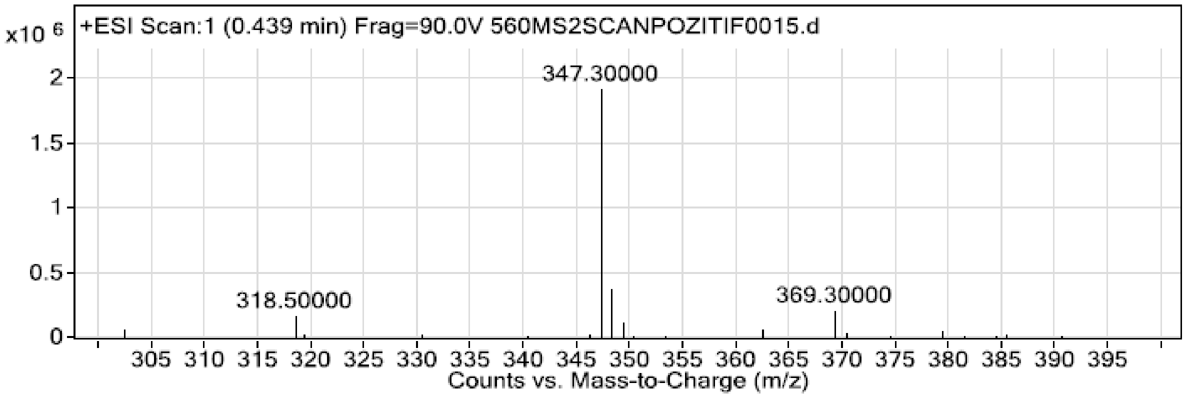

S20: ^{1}H NMR ($CDCl_3$), ^{13}C NMR ($CDCl_3$) and MS spectra of **e18**

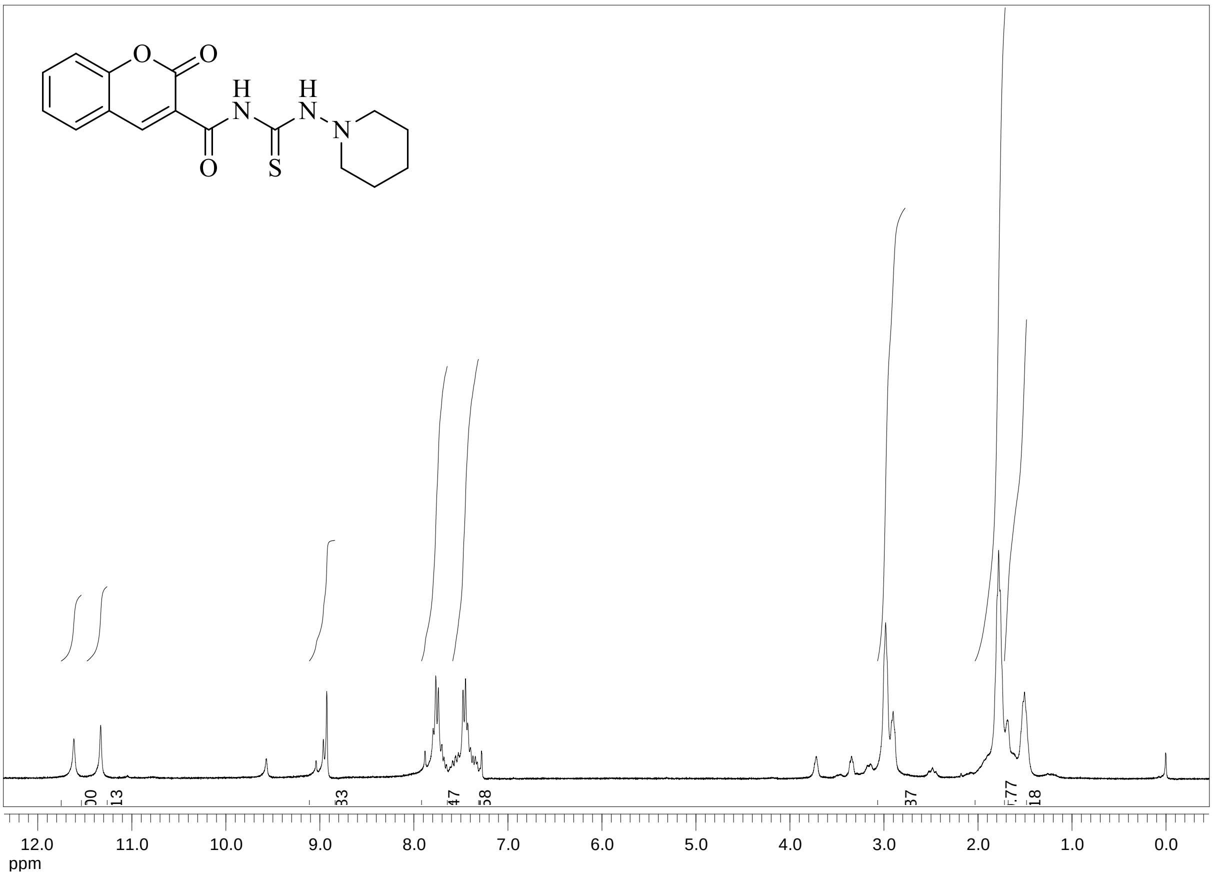

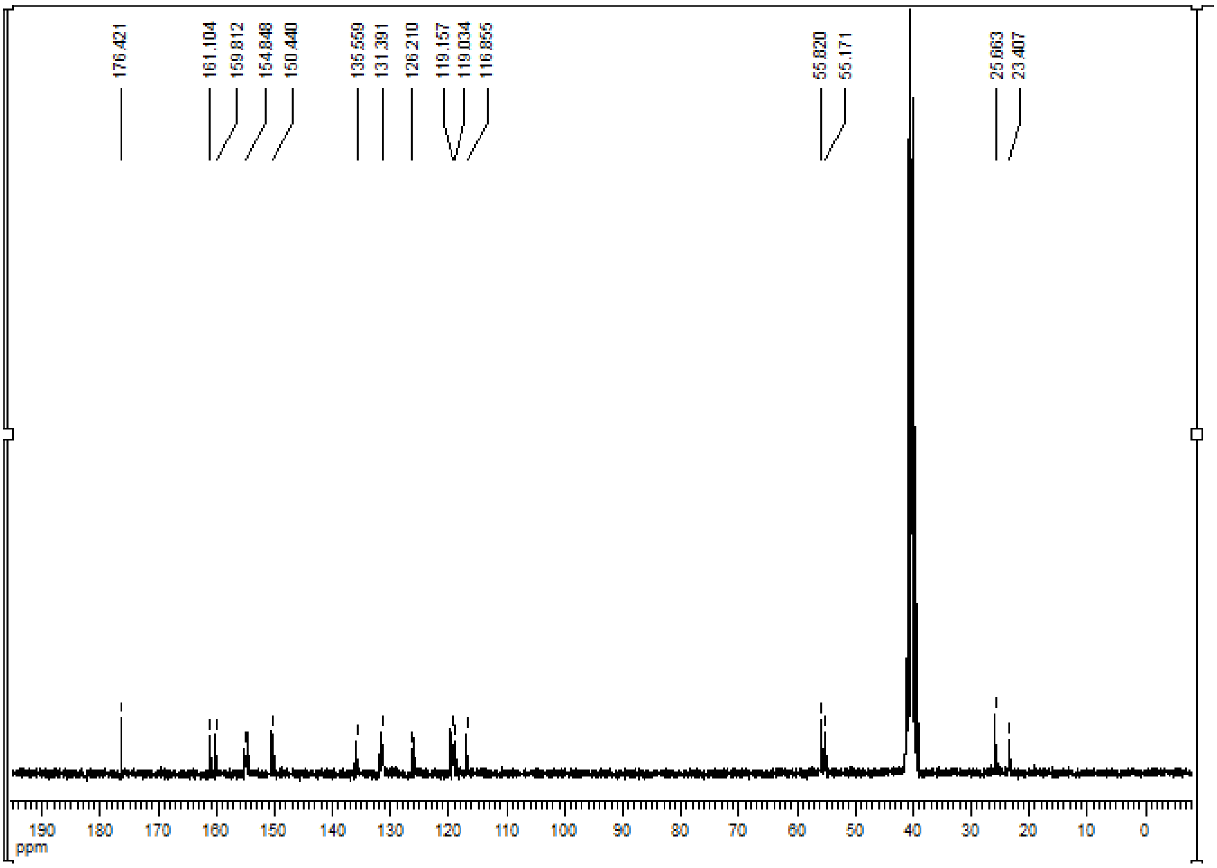
176.421
161.104
159.812
154.848
150.440
135.559
131.391
126.210
119.157
119.034
116.855
55.820
55.171
25.663
23.407
190 180 170 160 150 140 130 120 110 100 90 80 70 60 50 40 30 20 10 0
ppm

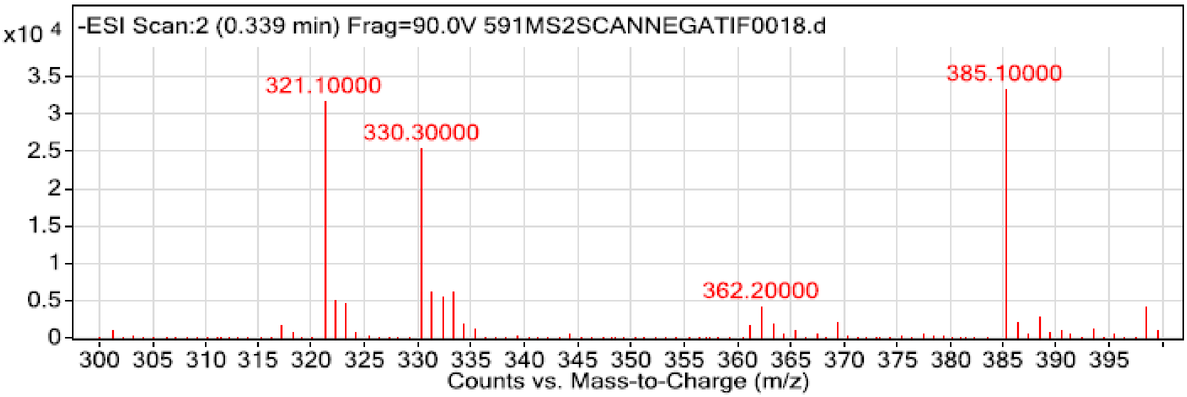
x10 4
-ESI Scan:2 (0.339 min) Frag=90.0V 591MS2SCANNEGATIF0018.d
321.10000
330.30000
385.10000
362.20000
3.5
3
2.5
2
1.5
1
0.5
0
300 305 310 315 320 325 330 335 340 345 350 355 360 365 370 375 380 385 390 395
Counts vs. Mass-to-Charge (m/z)

S21: [1] H NMR ($CDCl_3$), ^{13}C NMR ($CDCl_3$) and MS spectra of **e19**

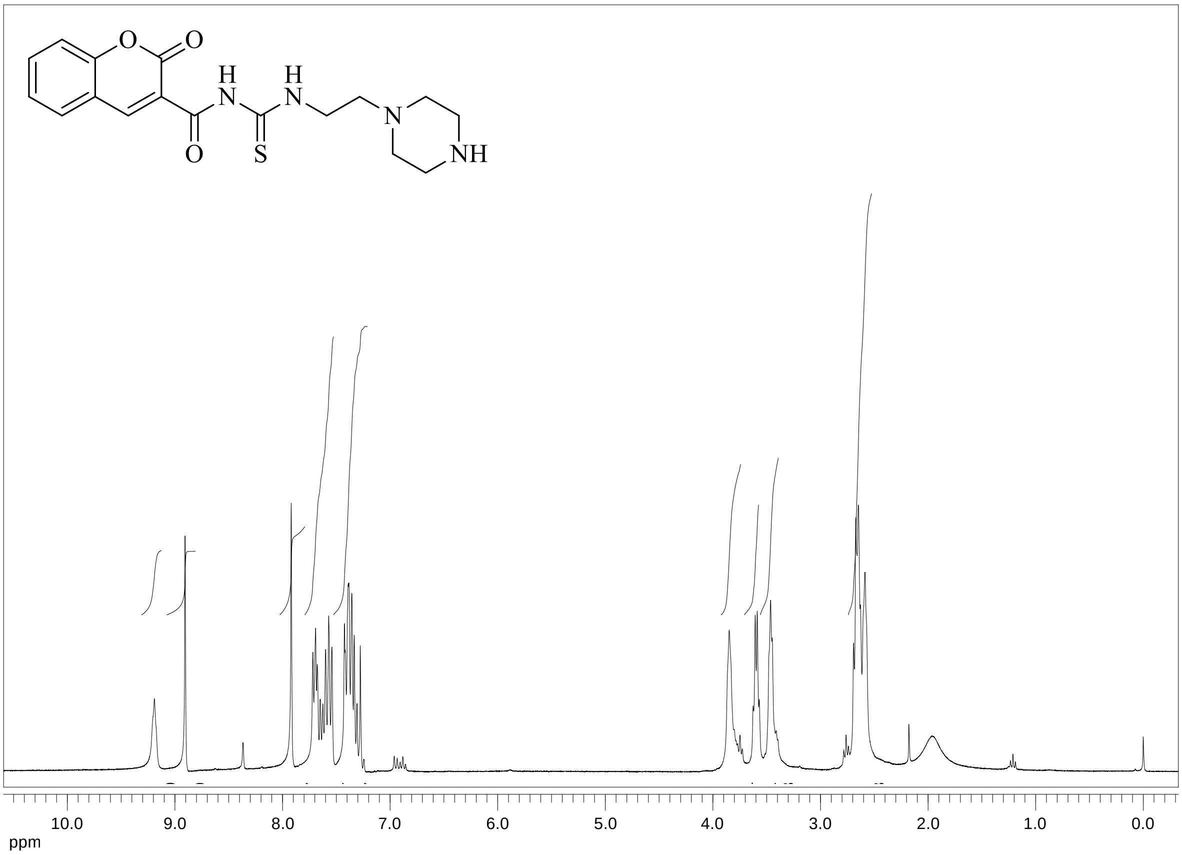

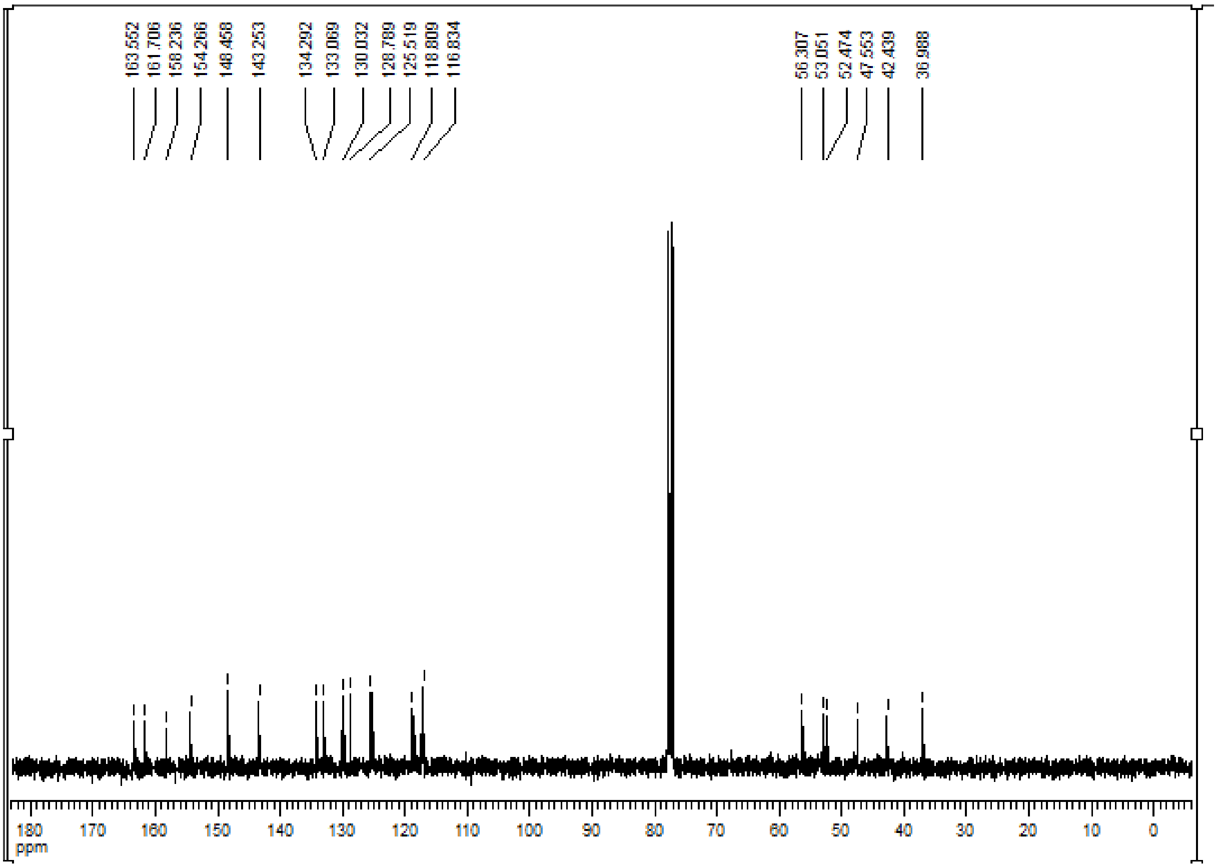

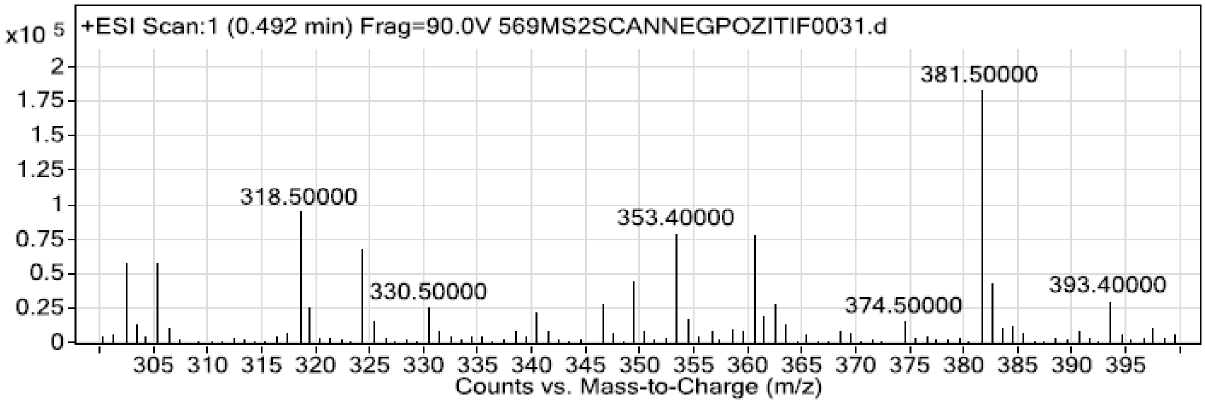

S22: [1] H NMR ($CDCl_3$), ^{13}C NMR ($CDCl_3$) and MS spectra of **e20**

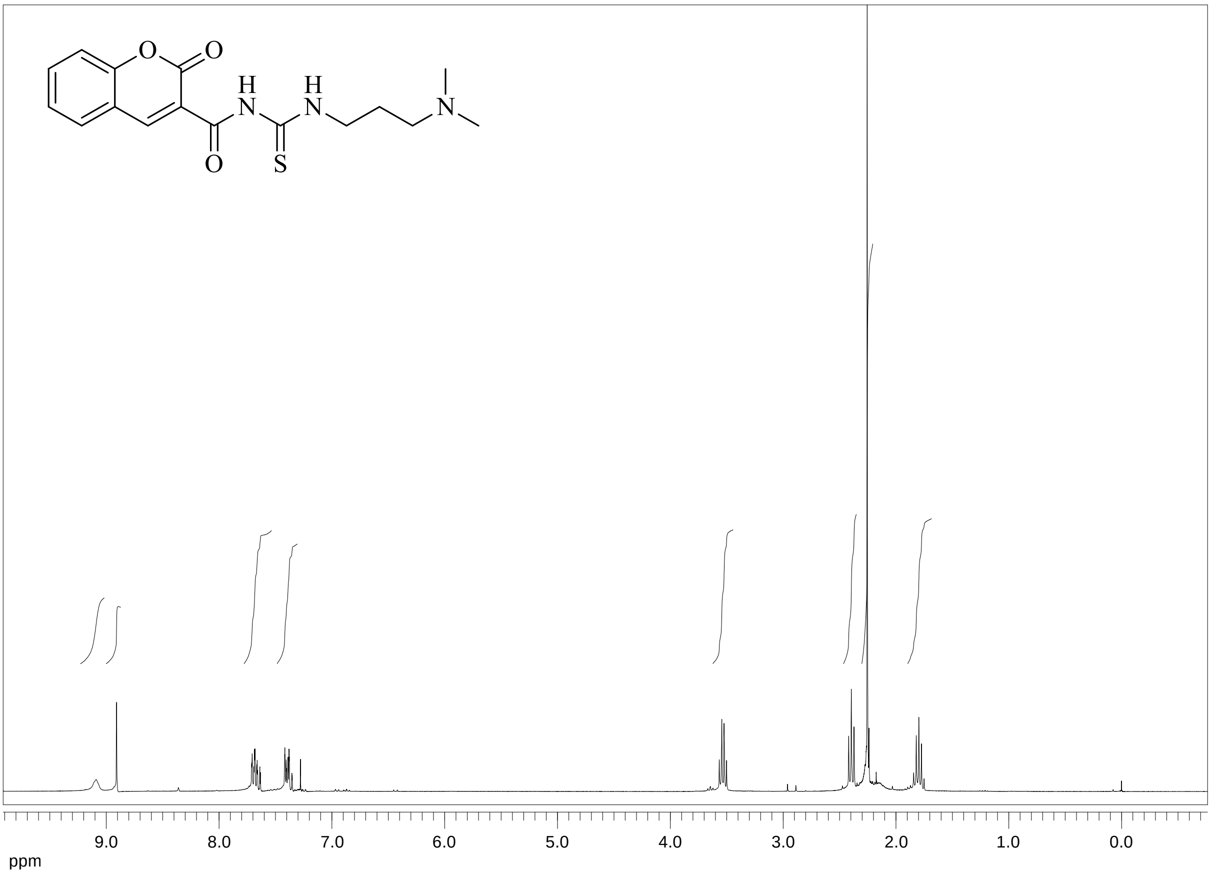

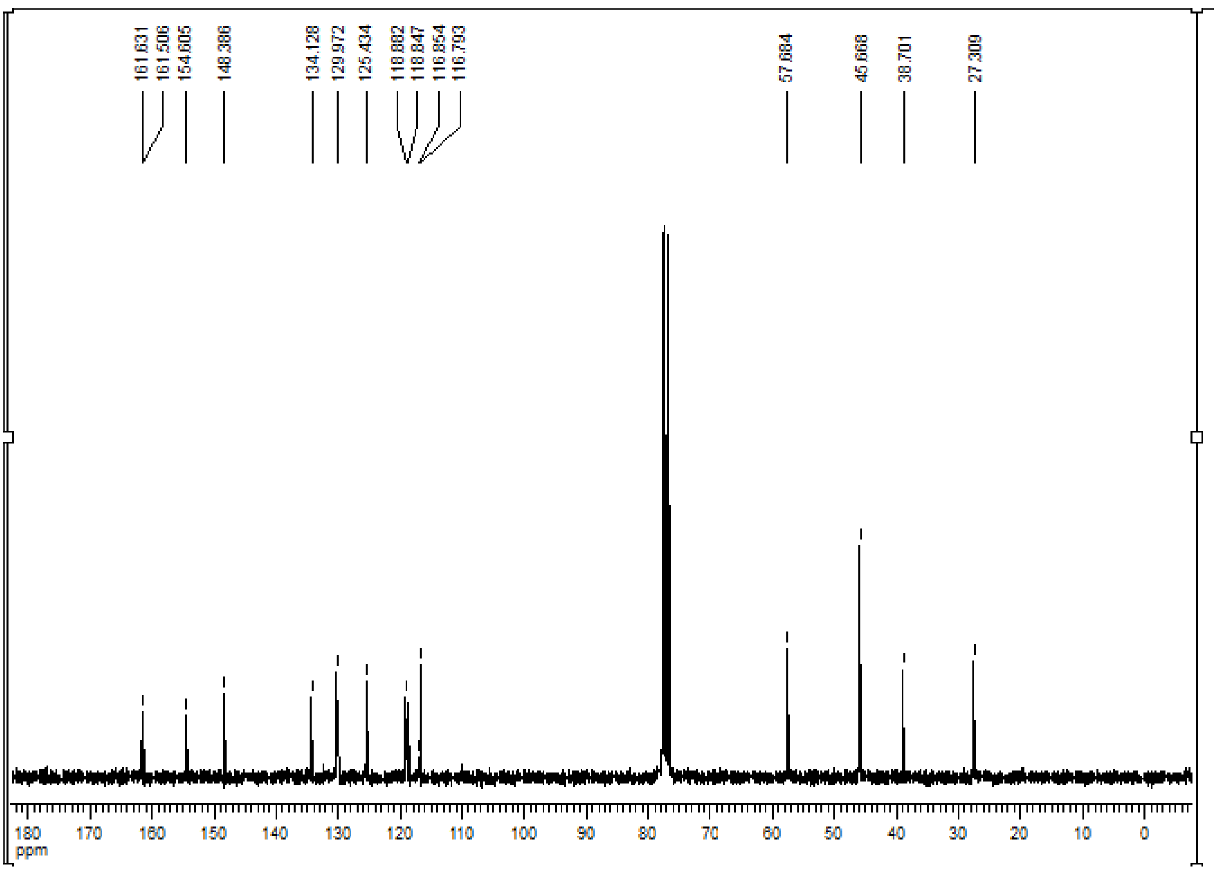
161.631
161.506
154.605
148.386
134.128
129.972
125.434
118.882
118.847
116.854
116.793
57.684
45.668
38.701
27.309
180 170 160 150 140 130 120 110 100 90 80 70 60 50 40 30 20 10 0
ppm

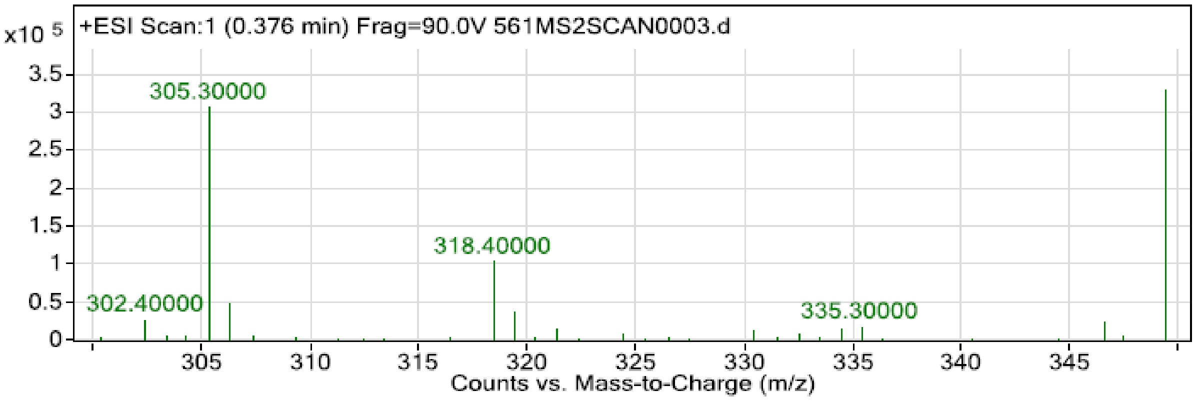
x10 5
+ESI Scan:1 (0.376 min) Frag=90.0V 561MS2SCAN0003.d
3.5
3
2.5
2
1.5
1
0.5
0
305.30000
318.40000
302.40000
335.30000
305 310 315 320 325 330 335 340 345
Counts vs. Mass-to-Charge (m/z)
